# CRISPR-based kinome-screening revealed MINK1 as a druggable player to rewire 5FU-resistance in OSCC through AKT/MDM2/p53 axis

**DOI:** 10.1038/s41388-022-02475-8

**Published:** 2022-10-01

**Authors:** Sibasish Mohanty, Pallavi Mohapatra, Omprakash Shriwas, Shamima Azma Ansari, Manashi Priyadarshini, Swatismita Priyadarsini, Rachna Rath, Mahesh Sultania, Saroj Kumar Das Majumdar, Rajeeb K. Swain, Rupesh Dash

**Affiliations:** 1 Institute of Life Sciences, Bhubaneswar, Odisha 751023 India; 2 Regional Center for Biotechnology, Faridabad, India; 3 KIIT School of Biotechnology, KIIT University, Bhubaneswar, India; 4 Department of Oral & Maxillofacial Pathology, SCB Dental College & Hospital, Cuttack, Odisha 753007 India; 5 Department of Surgical Oncology, All India Institute of Medical Sciences, Bhubaneswar, Odisha 751019 India; 6 Department of Radiotherapy, All India Institute of Medical Sciences, Bhubaneswar, Odisha 751019 India

**Keywords:** Cancer therapeutic resistance, Cell signalling

## Abstract

Cisplatin, 5FU and docetaxel (TPF) are the most common chemotherapy regimen used for advanced OSCC. However, many cancer patients experience relapse, continued tumor growth, and spread due to drug resistance, which leads to treatment failure and metastatic disease. Here, using a CRISPR/Cas9 based kinome knockout screening, Misshapen-like kinase 1 (MINK1) is identified as an important mediator of 5FU resistance in OSCC. Analysis of clinical samples demonstrated significantly higher MINK1 expression in the tumor tissues of chemotherapy non-responders as compared to chemotherapy responders. The nude mice and zebrafish xenograft experiments indicate that knocking out MINK1 restores 5FU mediated cell death in chemoresistant OSCC. An antibody based phosphorylation array screen revealed MINK1 as a negative regulator of p53. Mechanistically, MINK1 modulates AKT phosphorylation at Ser473, which enables p-MDM2 (Ser 166) mediated degradation of p53. We also identified lestaurtinib as a potent inhibitor of MINK1 kinase activity. The patient derived TPF resistant cell based xenograft data suggest that lestaurtinib restores 5FU sensitivity and facilitates a significant reduction of tumor burden. Overall, our study suggests that MINK1 is a major driver of 5FU resistance in OSCC. The novel combination of MINK1 inhibitor lestaurtinib and 5FU needs further clinical investigation in advanced OSCC.

## Introduction

Majority of head and neck cancer is originated from mucosal epithelium collectively termed as Oral squamous cell carcinomas (OSCC) [1]. It is the most prevalent neoplasm in developing country like India with approximately 80,000 new cases diagnosed every year [2]. Unfortunately, most of the patients with advanced OSCC are without having any preclinical history of pre malignant lesions. The treatment modalities for advanced OSCC include surgical removal of tumor followed by concomitant chemoradiotherapy. Neoadjuvant chemotherapy is frequently prescribed for surgically unresectable OSCC tumor [3]. However, despite of having all these treatment modalities, the 5-year survival rate of advanced tongue OSCC is less than 50%. Chemoresistance is one of the major causes of treatment failure in OSCC [4]. The chemotherapeutic regimen used for OSCC are cisplatin, 5FU and Docetaxel (TPF) [3]. Though chemotherapy drugs show initial positive response, tumor acquires resistance gradually and patients experience continued tumor growth and metastatic disease.

Reprogramming resistant cells to undergo drug induced cell death is a feasible approach to overcome drug resistance. This can be achieved by identifying the causative factors for acquired chemoresistance and discovering novel agents to target critical causative factors, which will restore drug-induced cell death in chemoresistant OSCC. Kinases, which transfer a reversible phosphate group to proteins, play important role in regulating several phenotypes of carcinogenesis including growth, proliferation, angiogenesis, metastasis and evasion of antitumor immune responses [5]. There are approximately 538 kinases in human that are known to regulate different kinase signaling. A few of them are also known to regulate drug resistance in HNSCC. A kinome study revealed microtubule-associated serine/threonine kinase 1 (MAST1) as a major driver of cisplatin resistance in HNSCC. MAST1 inhibitor lestaurtinib efficiently sensitized chemoresistant cells to cisplatin. Overall, the study suggests that MAST1 is a viable target to overcome cisplatin resistance [6]. However, studies relating kinase mediators of 5FU resistance in OSCC are limited.

The goal of this study is to find out the potential kinase(s) those are major driver(s) of 5FU resistance in OSCC, for which a CRISPR based kinome screening was employed on 5FU resistant OSCC lines. The top ranked protein MINK1 was selected for validation in multiple cell lines and patient derived cells. MINK1 belongs to germinal center kinase (GCK) family and it is involved in regulation of several important signaling cascades [7]. Recently, it is reported that MINK1 can regulate the planner cell polarity, which is essential for spreading of cancer cells. The PRICKLE1 encodes the planner cell polarity protein that binds to MINK1 and RICTOR (a member in mTOR2 complex) and this complex regulates the AKT meditated cell migration. Selectively targeting either of MINK1, PRICKLE1 or RICTOR can significantly decrease the migration of cancer cell in breast carcinomas [8]. Ste20-related kinase, misshapen (msn), a Drosophila homolog of MINK1 regulates embryonic dorsal closure through activation of c-jun amino-terminal kinase (JNK) [9]. In this study, following the identification of MINK1 as a major driver of 5FU resistance, lestaurtinib was found to inhibit MINK1 kinase activity, which can reverse 5FU mediated cell death in chemoresistant OSCC lines. Ultimately, we demonstrated that MINK1 regulates p53 in 5FU resistant OSCC. MINK1 activates AKT by phosphorylation at Ser473, which phosphorylates MDM2 at Ser166, the later in turn triggers degradation of p53.

## Results

### Establishment and characterization of 5FU resistant OSCC lines

The 5FU resistant OSCC lines were established by prolonged treatment of 5FU to OSCC cell lines as described in supplementary materials and methods. Monitoring the cell viability of 5FU sensitive (5FUS) and resistant (5FUR) pattern of H357, SCC4 and SCC9 cell lines by MTT assay suggest that 5FUR cells achieved acquired resistance (Fig. S1A, B). Further, reduced expression of γ-H2AX in 5FU treated resistant cells in contrast with 5FU treated sensitive cells also confirmed the acquired resistance to 5FU in 5FUR cells (Fig. S1C). Enhanced cancer like stem cells (CSCs) and elevated expression of ATP-binding cassette (ABC) transporters are the hallmarks of chemoresistant cells. qRT-PCR data suggest that CSC markers (SOX2, OCT4 and NANOG) as well as majority of ABC transporters’ expression were elevated in 5FUR cells as compared to 5FUS cells (Fig. S1D, E). The flow cytometry data suggests that the percentage of thymidylate synthase positive cells (a hall mark of 5FU resistance) is significantly higher in 5FU resistant cells as compared to 5FU sensitive OSCC lines (Fig. S1F, G). Similarly, the expression of thymidylate synthase is elevated in 5FU resistant cells as compared to sensitive cells (Fig. S1H).

### Kinome wide screening identifies MINK1 as a potential driver of 5FU resistance in OSCC

To understand the role of potential kinase signaling in 5FU resistance in OSCC, a CRISPR based kinome-wide screening was performed using a lentiviral sgRNA library knocking out 840 kinases individually with a total number of 3214 sgRNA constructs. To target the individual kinase, up to 4 sgRNA lentiviral constructs were pooled together. For kinome screening, Cas9 stable overexpressing 5FU resistant OSCC lines were established (Fig. S2), which showed similar drug resistant pattern with parental 5FUR OSCC lines (Fig. S3A). We also determined the polybrene and puromycin tolerance concentration in Cas9 overexpressing clones (Fig. S3B, C). The 5FUS and 5FUR lines were treated with 5FU and cell death was measured in high content analyzer using a fluorescent cell viability dye. The data suggest significantly lower cell death in 5FUR cells as compared to 5FUS cells, which is in harmony with the previously measured 5FU tolerance in both lines (Fig. S4A–C). The screening protocol was optimized using appropriate positive and negative control. When 6TG (6-Thioguanine) was treated to HPRT1 (Hypoxanthine Phosphoribosyltransferase 1) KO lines, which is used as positive control for screening, it showed resistance to cell death, whereas HPRT1 WT cells were sensitive to 6TG, suggesting optimized screening protocol (Fig. S4D, E). As a negative control, lentivirus expressing scrambled sgRNA was used.

**Fig. 1:**
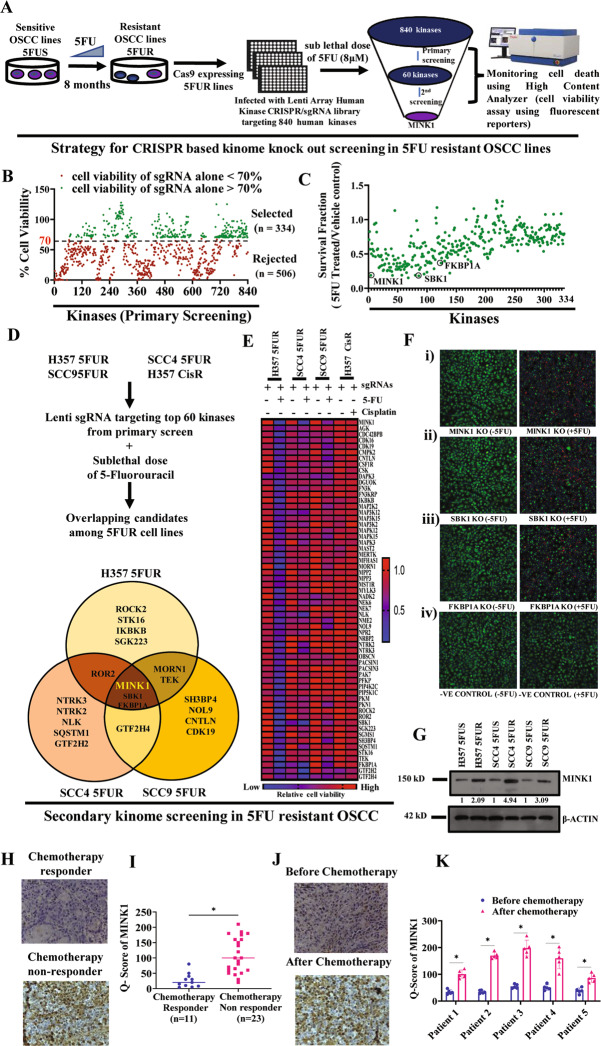
CRISPR based Kinome screening revealed MINK1 as a potential mediator for 5FU resistance in OSCC. **A** Schematic presentation of approach for CRSPR/Cas9 based kinome knockout screening to discover the potential kinase responsible for 5FU resistance in OSCC. **B**, **C** Primary screening of 840 kinases was performed in H357 5FUR line with sublethal dose of 5FU (8 μM). The kinases (*n* = 506 nos) whose knockout alone induced significantly higher cell death (>30%) depicted in red were excluded. From the rest of the kinases (*n* = 334 nos) depicted in green, the survival fraction (5FU treated/Vehicle Control) was determined and top 60 candidates having lowest survival fraction were considered for secondary screening described in panel (**C**). **D** For secondary screening with top 60 kinases, four cell lines were considered i.e., H357 5FUR, SCC4 5FUR, SCC9 5FUR and H357CisR. After overlapping all three 5FUR cell lines, MINK1, SBK1, FKBP1A were found to be the common kinases among them. MINK1 was selected as a potential kinase target purely based on having the lowest survival fraction among all common candidates. **E** Heat map based on % of cell viability with or without treatment of 5FU or cisplatin followed by knocking out 60 individual kinases in H357 5FUR, SCC4 5FUR, SCC9 5FUR and H357 CisR cells. **F** The fluorescent images acquired from high content analyzer with indicated treated group in H357 5FUR lines during kinome screening. **G** Lysates were collected from indicated cells and immunoblotting (*n* = 3) was performed with indicated antibodies. **H** Protein expression of MINK1 was analyzed by IHC in chemotherapy- responder and chemotherapy-non-responder OSCC tumors. Scale bars: 50 μm. **I** IHC scoring for MINK1 from panel (**H**) (Q Score = Staining Intensity × % of Staining), (Median, *n* = 11 for chemotherapy-responder and *n* = 23 for chemotherapy-non-responder) *P < 0.05 by 2-tailed Student’s *t* test. **J** Protein expression of MINK1 was analyzed by immunohistochemistry (IHC) in pre- and post-TPF treated paired tumor samples from chemotherapy-non-responder patients. Scale bars: 50 μm. **K** IHC scoring for MINK1 from panel (**J**) (Q Score = Staining Intensity × % of IHC Staining). *P < 0.05 by 2-tailed Student’s *t* test.

For primary screening, the 5FU resistant line (H357 5FUR) was transduced with a lentivirus based library containing sgRNAs targeting each of the 840 individual kinases, after which sub lethal dose of 5FU was treated for 48 h followed by measuring cell death in high content analyzer using a fluorescent cell viability dye (Fig. 1A). From primary screening, 334 kinases out of 840 were selected for further consideration by rejecting rest of sgRNA clones which alone induced cell death more than 30% (Fig. 1B, C and Table S1). The 60 candidate kinases having lowest survival fraction score were evaluated in the secondary screening using three more chemoresistant lines i.e. SCC4 5FUR, SCC9 5FUR and H357 CisR along with H357 5FUR. From the primary and secondary screening, MINK1, SBK1 and FKBP1A emerged as the only three common kinases among the 5FUR lines with MINK1 having the lowest survival fraction score, which sensitized the chemoresistant cells to 5FU the most (Fig. 1C–F and Table S2). In secondary screening, MINK1 knock out showed minimal efficacy in sensitizing cisplatin resistant cell lines to cisplatin (Fig. 1E and Table S2), indicating the specific role of MINK1 towards acquired 5FU resistance. Next, we found the expression of MINK1 is significantly higher in 5FUR lines as compared to 5FUS lines of OSCC (Fig. 1G). With the evaluation of clinical samples, the expression of MINK1 was found to be elevated in tumor tissues of chemotherapy non-responders as compared to chemotherapy responders (Fig. 1H, I and Table S3). We also evaluated the MINK1 expression in drug-naive and post-CT non-responder paired tumor samples from the same patient and observed that the post–CT-treated tumor samples showed higher MINK1 expression (Fig. 1J, K and Table S3).

### MINK1 is an important target to overcome 5FU resistance in OSCC

**Fig. 2:**
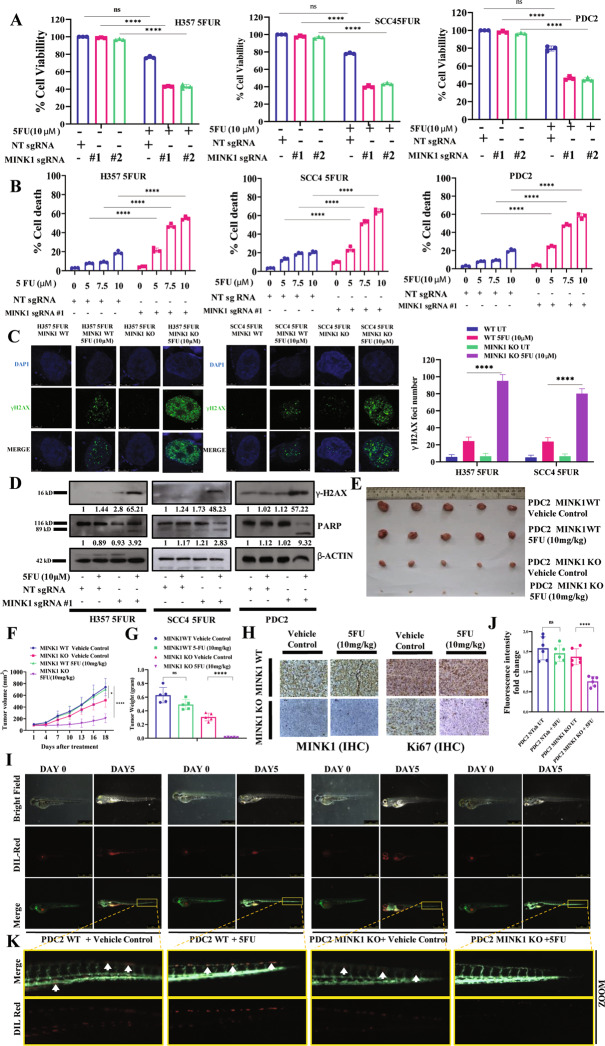
Selectively targeting MINK1 restores 5FU induced cell death in chemoresistant OSCC. **A** 5FU resistant cells stably expressing MINK1sgRNA (#1 and #2) and NTsgRNA were treated with 5FU for 48 h and cell viability was determined by MTT assay (*n* = 3 and 2-way ANOVA, ****P < 0.0001). **B** Indicated cells were treated with 5FU for 48 h, after which cell death was determined by annexin V/7AAD assay using flow cytometer. Bar diagrams indicate the percentage of cell death (early and late apoptotic) with respective treated groups (Mean ± SEM, *n* = 3, Two-way ANOVA, ****P < 0.0001). **C** Left panel: Indicated cells were treated with 10 μM of 5FU for 48 h, after which immunostaining was performed for γ-H2AX. Right panel: The number of foci from panel C are indicated as bar diagram. Two-way ANOVA, ****P < 0.0001. **D** Indicated cells were treated with 5FU for 48 h and immunoblotting (*n* = 3) was performed with indicated antibodies. **E** PDC2 MINK1WT cells were implanted in right upper flank of athymic male nude mice and PDC2 MINK1KO cells were implanted in left upper flank, after which they were treated with 5FU at indicated concentration. At the end of the experiment mice were euthanized, tumors were isolated and photographed (*n* = 5). **F** Tumor growth was measured in indicated time points using digital slide caliper and plotted as a graph (mean ± SEM, *n* = 5, Two-way ANOVA, *P < 0.05). **G** Bar diagram indicates the tumor weight measured at the end of the experiment (mean ± SEM, *n* = 5, Two-way ANOVA, ****P < 0.0001). **H** After completion of treatment, tumors were isolated and paraffin-embedded sections were prepared as described in materials and methods to perform immunohistochemistry with indicated antibodies. Scale bars: 50 μm. **I** Lateral view of fluorescent transgenic [Tg(fli1:EGFP)] zebrafish embryos at Day 0 and Day 5 injected with Dil-Red stained PDC2 control and MINK1 KO cells with and without treatment of 5FU. **J** The tumor growth and migration were assessed by an increase in fluorescence intensity on the 5th day compared to the day of injection. The quantitation of fluorescence intensity (**I**) was performed using ImageJ software and represented as fold change of fluorescence intensity where day 0 reading was taken as baseline (mean ± SEM, *n* = 6, Two-way ANOVA, ****P < 0.0001). **K** Zoomed image of distal part of embryo (5days post injection) to monitor migration of tumor cells.

To confirm the finding from the kinome screening, MINK1KO (knock out) clones were generated, using lentivirus expressing two different sgRNAs (sgRNA#1 and sgRNA#2), in Cas9 overexpressing 5FUR OSCC lines and patient derived line 2 (PDC2) (Fig. S5A). PDC2 was isolated and characterized earlier from tumor of chemotherapy-non-responder patient, who was treated with neoadjuvant TPF without any response [10]. The colony forming, MTT and spheroid assay data suggest that knocking out MINK1 significantly reduced the cell viability of chemoresistant cells treated with 5FU (Fig. S5B, C and Fig. 2A). For rest of the experiments sgRNA1 was used to knock out MINK1. Similarly, knocking out MINK1 induced 5FU mediated cell death in chemoresistant cells (Fig. 2B). Enhanced p-H2AX and cleaved PARP was observed in MINK1KO cells followed by treatment with 5FU indicating the potential role of MINK1 in mediating 5FU resistance (Fig. 2C, D). Further, to test the in vivo efficacy of the kinome screening data, we implanted PDC2 MINK1WT cells into right upper flank and PDC2 MINK1KO cells into the left upper flank of nude mice followed by treatment with 5FU. Treatment with 5FU (10 mg/kg) significantly reduced the tumor burden in the MINK1KO but not in MINK1WT group (Fig. 2E–G). Immunohistochemistry data suggest markedly decreased cell proliferation signal (Ki67) in 5FU-treated MINK1KO tumors (Fig. 2H). Earlier it was proven that selective knockdown of MINK1 decreases the migration of human breast cancer lines [8]. To evaluate whether depletion of MINK1 also reduces migration of chemoresistant OSCC lines and PDC2, Boyden chamber assays and scratch/wound healing assays were performed. The data suggest that knock out of MINK1 followed by treatment with 5FU significantly reduces the relative number of migrated cells (Fig. S6A). Similarly, scratch area analysis suggest that percentage of scratch area is significantly higher when 5FU is treated to MINK1KO drug resistant cells (Fig. S6B, C). Further, our qPCR and immunoblotting data suggests that expression level of ABC transporters and thymidylate synthase are significantly lower in MINK1KO drug resistant cells. On the other hand, when MINK1 was ectopically overexpressed in sensitive OSCC lines, we found increased expression of thymidylate synthase (Fig. S7A–C). Next, we used Zebrafish (*Danio rerio*) [Tg(fli1:EGFP)] tumor xenograft model to further validate our findings. Equal number of the PDC2WT and PDC2MINK1 KO cells were stained with Dil (1,1′-Dioctadecyl-3,3,3′,3′-Tetramethylindocarbocyanine Perchlorate) and injected into perivitelline space of 48-h post fertilized zebrafish embryos. After 3 days of injection, embryos were treated with vehicle control or 5FU (500 µM). After 5 days of injection, the primary tumors and metastatic distribution of cancer cells were documented using a fluorescence microscope. The tumor growth, as measured by fluorescence intensity of primary tumors, was found to be significantly reduced in the MINK1 KO group with the treatment of 5FU (Fig. 2I, J). Also, cancer cells showed reduced distal migration from the primary site in the case of MINK1 KO 5FU treated group (Fig. 2K). These data indicate MINK1 dependency of 5FU resistant OSCC. To explore potential role of MINK1 in 5FU sensitive cells, we knocked out MINK1 in 5FU sensitive cells and measured the cell viability by MTT assay, which suggest that knock out of MINK1 results in higher sensitivity to 5FU as compared to MINK1 WT cells (Fig. S8A, B).

### Ectopic expression of MINK1 promotes 5FU resistance in OSCC

**Fig. 3:**
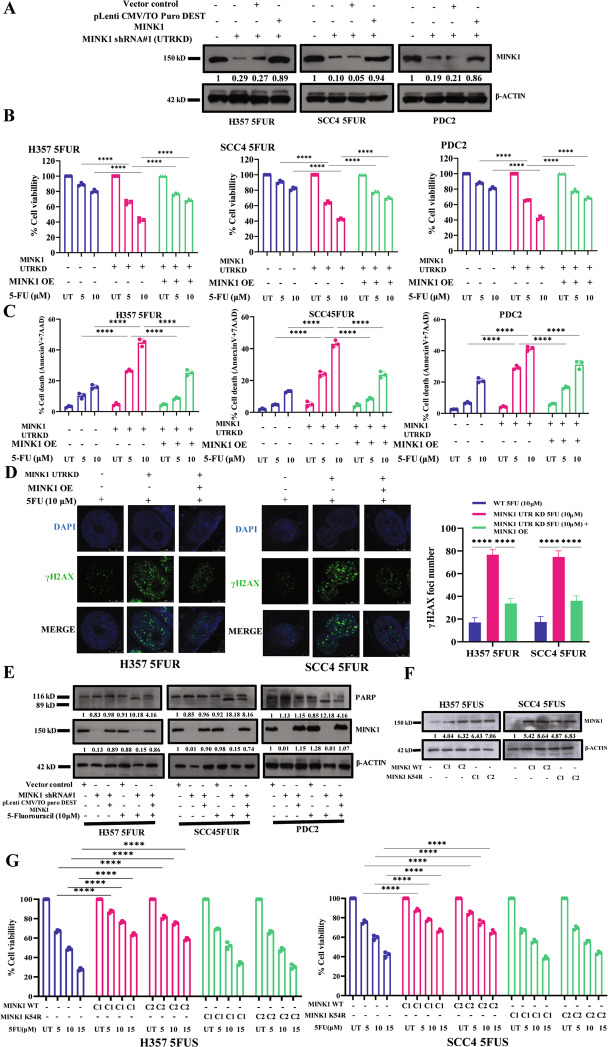
Ectopic overexpression of MINK1 rescued the drug resistant phenotype in MINK1KD drug resistant OSCC. **A** Using a lentiviral approach, 5FU resistant OSCC lines and PDC2 were stably transfected with ShRNA which targets 3′UTR of MINK1 mRNA (MINK1 UTRKD). For ectopic overexpression, pLenti CMV/TO Puro DEST MINK1 and control vector were transiently transfected to indicated MINK1 UTRKD cells and immunoblotting (*n* = 3) was performed with indicated antibodies. **B** MINK1 was ectopically overexpressed in 5FUR cells stably expressing MINK1ShRNA targeting 3′UTR and treated with 5FU at indicated concentration for 48 h, after which cell viability was determined by MTT assay (*n* = 3), 2-way ANOVA, ****P < 0.0001. **C** Cells were treated as indicated in (**B**) panel and cell death (early and late apoptotic) was determined by annexin V/7AAD assay using flow cytometer. Bar diagrams indicate the percentage of cell death with respective treated groups (Mean ± SEM, *n* = 3), 2-way ANOVA, ****P < 0.0001. **D** Left panel: MINK1 was overexpressed in 5FUR cells stably expressing MINK1ShRNA targeting 3′UTR and treated with 5FU for 48 h, after which immunostaining was performed for γ-H2AX as described in materials and methods. Right panel: The number of foci from panel (**D**) are indicated as bar diagram. Two-way ANOVA, ****P < 0.0001. **E** MINK1 was overexpressed in chemoresistant cells stably expressing MINK1ShRNA targeting 3′ UTR, followed by 5FU treatment for 48 h, and immunoblotting (*n* = 3) was performed with indicated antibodies. **F** 5FU sensitive cells were stably transfected with either pSilencer™ 4.1-CMV puro-MINK1WT or pSilencer™ 4.1-CMV puro-MINK1 K54R (kinase dead) followed by puromycin selection (up to 5 µg/ml). Two clones (C1 and C2) from each line were selected and immunoblotting was performed with indicated antibodies. **G** 5FU sensitive OSCC lines stably expressing MINK1 WT or MINK1 K54R (two clones C1 and C2 from each line) were treated with 5FU at indicated concentrations for 48 h, after which cell viability was determined by MTT assay (*n* = 3), 2-way ANOVA, ****P < 0.0001.

To confirm the potential role of MINK1 in 5FU resistance, we performed gain of function study. For this, using a lentiviral approach we generated MINK1ShRNA stable clones in 5FUR lines and PDC2 (MINK1UTRKD), where the shRNA targets the 3′UTR of MINK1 mRNA. For ectopic overexpression of MINK1, the MINK1UTRKD cells were transfected with pLenti CMV/TO Puro DEST MINK1 vector (Fig. 3A). The cell viability and cell death data suggest that knocking down MINK1 in 5FUR cells results in sensitizing the resistant cells to 5FU, however ectopic overexpression of MINK1 rescues the 5FU resistant phenotype (Fig. 3B, C). Similarly, immunostaining data suggest enhanced p-H2AX signal in MINK1UTRKD cells, whereas ectopic overexpression of MINK1 reduces the p-H2AX signal indicating rescue of 5FU resistance in OSCC cells (Fig. 3D). We also observed the rescue of cleaved PARP with ectopic expression of MINK1 suggesting reduced cell death (Fig. 3E). Further, when MINK1 was stably overexpressed in OSCC sensitive lines, cells showed resistance to 5FU induced cell death (Fig. 3F, G). On the other hand, when kinase dead form of MINK1 (K54R) was stably overexpressed in OSCC sensitive lines, cells did not show resistance to 5FU induced cell death. These data suggest that MINK1 kinase activity is required for 5FU resistance in OSCC (Fig. 3G). Kinase dead MINK1 was generated by mutating lysine (K) at 54 to Arginine (R).

### MINK1 downregulates the expression of p53 in chemoresistant OSCC through activation of AKT and MDM2

**Fig. 4:**
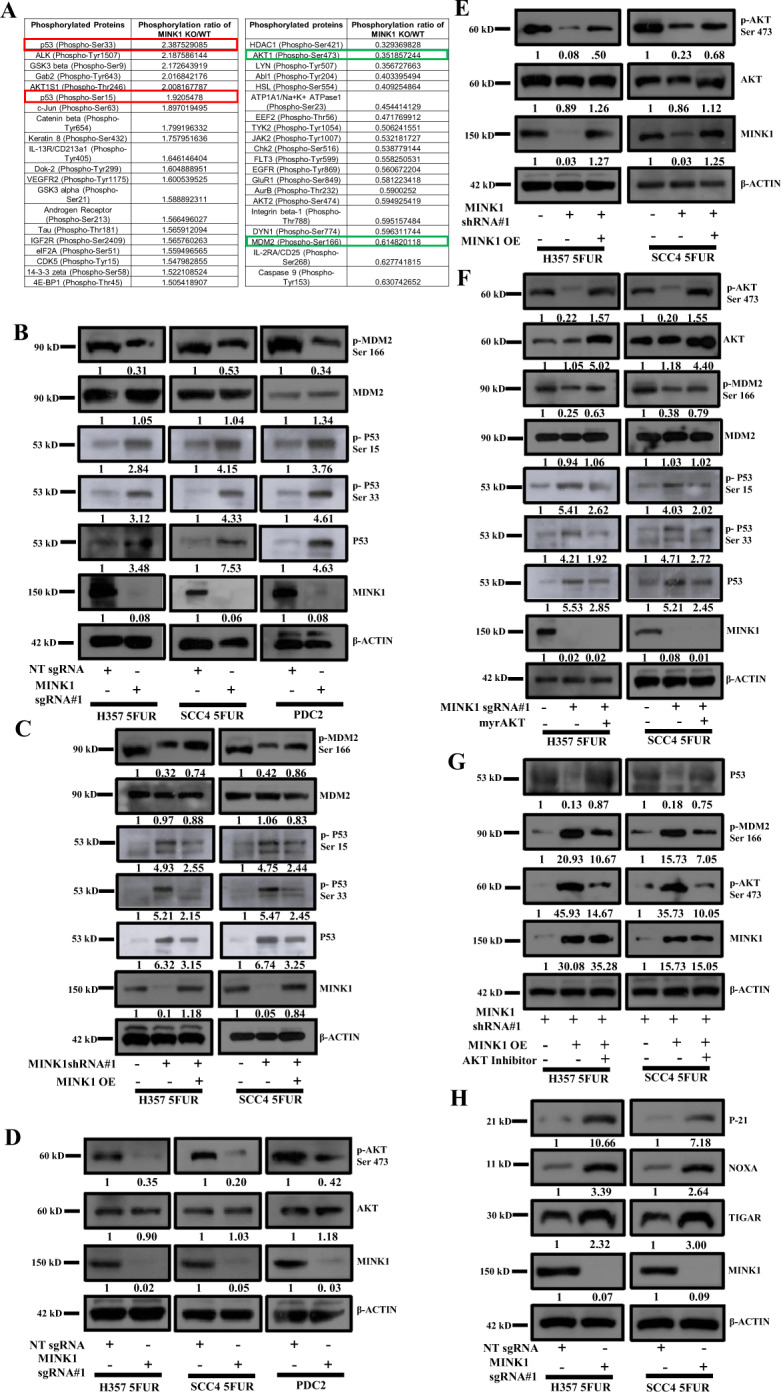
MINK1 regulates the expression of p53 in 5FU resistant OSCC lines through AKT/MDM2. **A** High-throughput phosphorylation profiling with 1318 site-specific antibodies from over 30 signaling pathways was performed in the lysates of MINK1KO and MINK1WT clones of H357 5FUR cells as described in methods. The top 20 upregulated phosphoproteins (MINK1 KO/MINK1WT) are represented in left panel, whereas top 20 downregulated phosphorylated proteins are represented in right panel. The upregulated targets considered in the study is marked in red box, whereas downregulated targets in green box. **B** Lysates were collected from indicated cells and immunoblotting (*n* = 3) was performed with indicated antibodies. **C** pLenti CMV/TO Puro DEST MINK1 (ectopic overexpression of MINK1) was transiently transfected in 5FUR lines stably expressing MINK1 ShRNA (targeting 3′UTR) and immunoblotting (*n* = 3) was performed with indicated antibodies. **D** Lysates were collected from indicated cells and immunoblotting (*n* = 3) was performed with indicated antibodies. **E** MINK1 was ectopically overexpressed in 5FUR lines stably expressing MINK1 ShRNA (targeting 3′UTR) and immunoblotting (*n* = 3) was performed with indicated antibodies. **F** pLNCX myr HA Akt1 (ectopic overexpression of myr AKT) was transiently transfected in indicated MINK1KO cells and immunoblotting (*n* = 3) was performed with indicated antibodies. **G** MINK1 was ectopically overexpressed in 5FUR lines stably expressing MINK1ShRNA (UTRKD) as described in panel C and treated with AKT inhibitor (Akti-1/2) for 24 h and immunoblotting (*n* = 3) was performed with indicated antibodies. **H** Lysates were collected from indicated cells and immunoblotting (*n* = 3) was performed with indicated antibodies.

To understand the specific role of MINK1 in 5FU resistant OSCC, we performed high-throughput phosphorylation profiling with 1318 site-specific antibodies from over 30 signaling pathways in 5FUR cells stably expressing MINK1KO and MINK1WT. From this study, phosphorylation of p53 at Ser33 and Ser15 were found to be significantly up regulated in MINK1KO cells as compared to MINK1WT cells. In addition to this, phosphorylation of AKT at Ser473 and phosphorylation of MDM2 at Ser166 were found to be down regulated in MINK1KO cells as compared to MINK1WT (Fig. 4A). Further, immunoblotting was performed to validate the finding of phospho-antibody array. The data suggest that phosphorylation of p53 at Ser15 and Ser33 is significantly upregulated and phosphorylation of MDM2 at Ser166 is profoundly downregulated in MINK1KO cells as compared to MINK1WT chemoresistant cells (Fig. 4B). Further, p53 expression was also found to be inversely correlated with MINK1 irrespective of its mutation status (Figs. 4B, S9A). Though p53 in SCC4 and H357 has missense mutations (codon 151 in SCC4 and codon 110 in H357), the p53 expression can be detectable both at m-RNA and protein level. While in case of SCC9, p53 has a deletion mutation (codon 274–285) with no detectable expression [11, 12]. MCF7, HEK 293 and HCT116 have wild type p53 expression [13]. Selective knock out of MINK1 in SCC45FUR, H3575FUR cells resulted in having significantly higher degree of sensitivity to 5FU as compared to SCC9 5FUR (Figs. 2A and S9B). Similarly, selective knock down of MINK1 in HCT116 cells showed higher sensitivity to 5FU as compared to NtShRNA cells (Fig. S9C). Next, when MINK1 was ectopically overexpressed in MINK1KD (shRNA targeting 3′UTR) clones, downregulation of p53, p-p53 (Ser33) and p-p53 (Ser15) were observed in chemoresistant OSCC lines (Fig. 4C). To explore if MINK1 kinase activity is required for regulation of P53, we stably overexpressed WT MINK1 and kinase dead MINK1 (K54R) in OSCC sensitive cells. The immunoblotting data suggest very little down regulation of p53 or p-p53 (Ser15) in cells stably expressing kinase dead domain form of MINK1 (Fig. S10). In harmony to our finding of phosphorylation array, p-AKT(Ser473) was found to be down regulated in MINK1 depleted cells, which was rescued with ectopic overexpression of MINK1 (Fig. 4D, E). To confirm the potential role of AKT in modulating MINK1 mediated p53 regulation, we ectopically overexpressed constitutively active AKT (myrAKT) in MINK1KO cells. The immunoblotting data suggest that expression of p53, p-p53 (Ser33) and p-p53 (Ser15) were downregulated when MyrAKT was overexpressed in MINK1KO cells (Fig. 4F). Similarly, when MINK1 over expressing cells were treated with AKT inhibitor (Akti-1/2), the p53 expression was rescued along with downregulation of p-MDM2 (Ser166) (Fig. 4G). p53 target genes were also evaluated and the immunoblotting data suggest that expression of p21, NOXA and TIGAR in MINK1KO clones were upregulated as compared to MINK1WT clones (Fig. 4H).

### Evaluation of lestaurtinib as MINK1 inhibitor to reverse 5FU resistance in OSCC

**Fig. 5:**
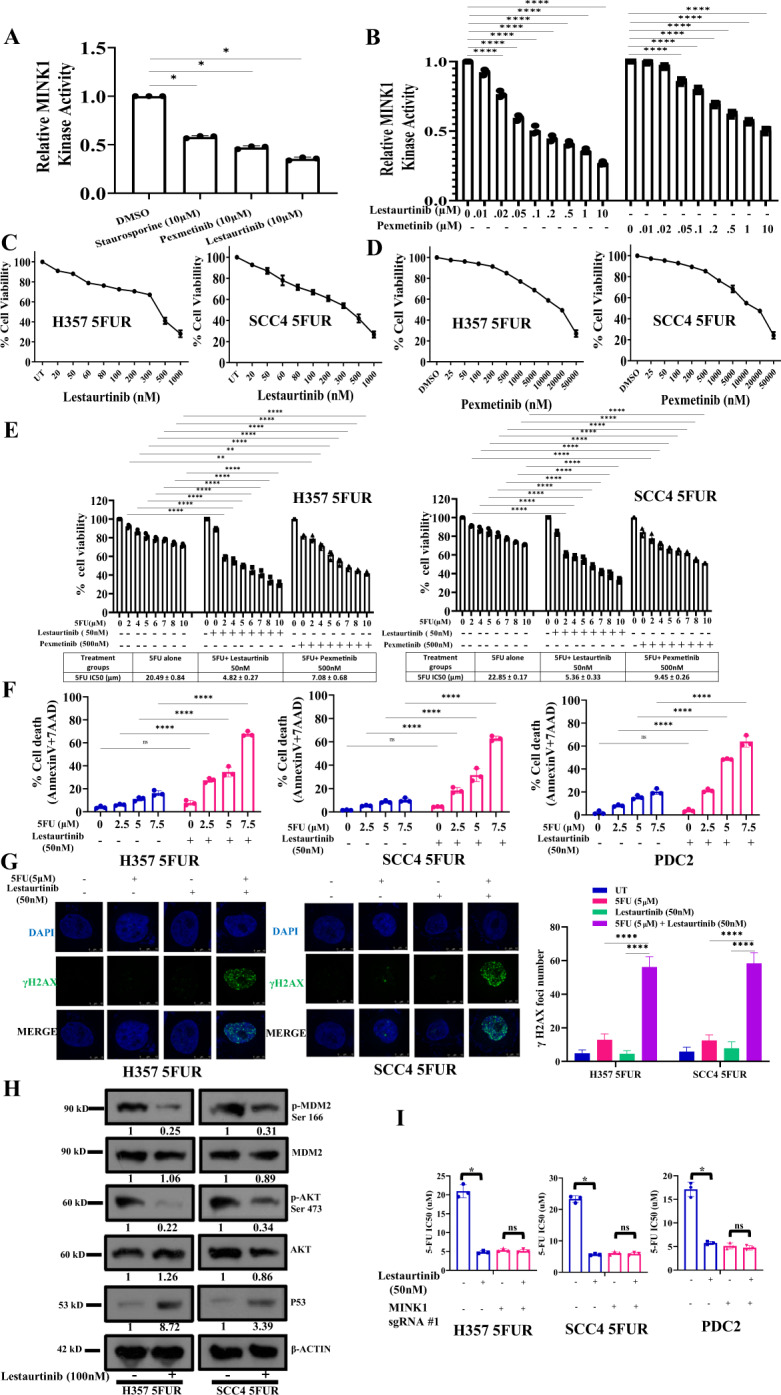
Evaluation of lestaurtinib as a MINK1 inhibitor to restore 5FU sensitivity in drug resistant OSCC. **A** In vitro MINK1 kinase assay was performed using three compounds potentially binding to MINK1 (based on IUPHAR database). All compounds (10 µM) were incubated with recombinant human MINK1 along with substrate MBP and ATP and further subjected to ADP-Glo™ Kinase Assay as described in materials and methods section (*n* = 3), one-way ANOVA, *P < 0.05. **B** Determination of EC50 value for kinase activity of top two MINK1 inhibitors selected from panel (**A**) (*n* = 3), one-way ANOVA, ****P < 0.0001. **C**, **D** The indicated cells were treated with indicated concentration of MINK1 inhibitors and cell viability was determined by MTT assay. Selection of highest dose of Lestaurtinib and Pexmetinib that does not affect cell viability when treated alone (viability > 80%) in 5FU resistant OSCC lines (*n* = 3). **E** 5FU resistant cells were treated with indicated doses of MINK1 inhibitors (50 nM Lestaurtinib, 500 nM Pexmetinib) in combination with increasing concentrations of 5FU for 48 h, after which cell viability was determined by MTT assay (*n* = 3), 2-way ANOVA, ****P < 0.0001. **F** 5FU resistant OSCC lines and PDC2 cells were treated with indicated doses of MINK1 inhibitors (50 nM Lestaurtinib, 500 nM Pexmetinib) in combination with increasing concentrations of 5FU for 48 h, after which cell death (early and late apoptotic) was determined by annexin V/7AAD assay using flow cytometer. Bar diagrams indicate the percentage of cell death with respective treated groups (Mean ± SEM, *n* = 3), Two-way ANOVA, ****P < 0.0001. **G** Left panel: Indicated 5FU resistant OSCC lines and PDC2 cells were treated with 5 μM of 5FU and/or 50 nM of Lestaurtinib for 48 h, after which immunostaining was performed for γ-H2AX as described in materials and methods. Right panel: The number of foci from panel (**G**) are indicated as bar diagram. Two-way ANOVA, ****P < 0.0001. **H** Indicated 5FU resistant OSCC lines and *P*DC2 cells were treated with Lestaurtinib for 48 h, after which immunoblotting (*n* = 3) was performed with indicated antibodies. **I** Effect on 5FU IC50 upon Lestaurtinib treatment in cells with or without MINK1 knockout in indicated 5FU resistant OSCC lines and PDC2 cells (*n* = 3), *P < 0.05 by 2-way ANOVA.

**Fig. 6:**
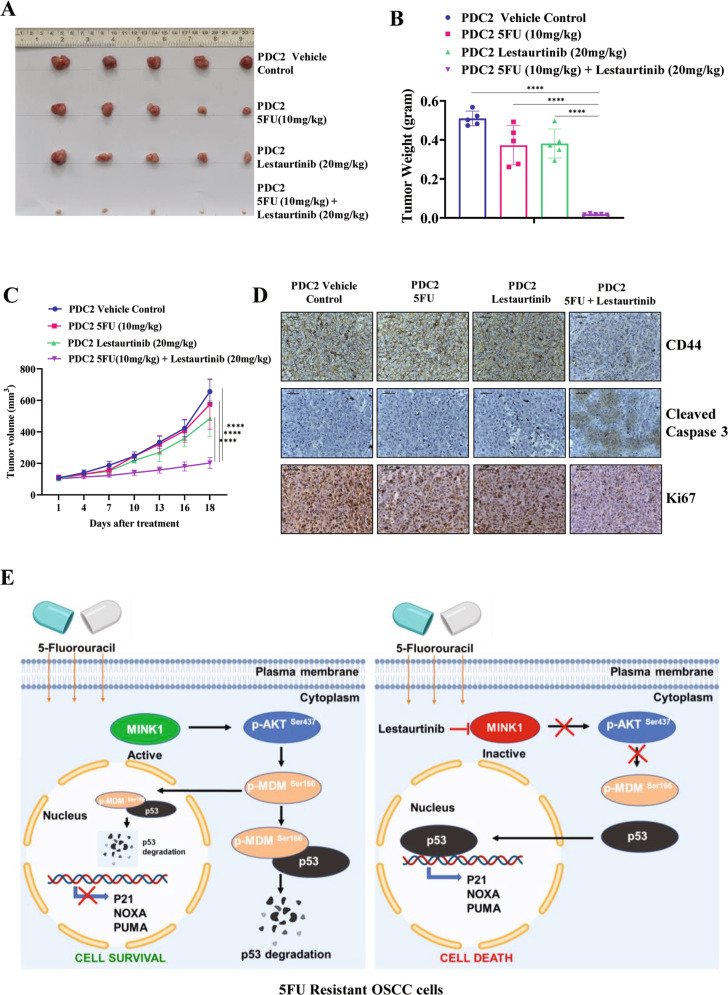
Lestaurtinib and 5FU synergistically reduced tumor burden in vivo in drug resistant OSCC. **A** Patient-derived cells (PDC2) were earlier established from tumor of chemotherapy (TPF) non-responder patient. PDC2 were implanted in the right upper flank of athymic male nude mice, after which they were treated (i.p) with 5FU and/or Lestaurtinib at indicated concentrations. At the end of the experiment mice were euthanized, and tumors were isolated and photographed (*n* = 5). **B** Bar diagram indicates the tumor weight measured at the end of the experiment (mean ± SEM, *n* = 5). Two-way ANOVA, ****P < 0.0001. **C** Tumor growth was measured at the indicated time points using digital slide caliper and plotted as a graph (mean ± SEM, *n* = 5). Two-way ANOVA, ****P < 0.0001. **D** After completion of treatment, tumors were isolated, and paraffin-embedded sections were prepared as described in Methods to perform IHC with indicated antibodies. Scale bars: 50 μm. **E** Schematic presentation of the mechanism by which MINK1 regulates p53 expression through AKT/MDM2 axis.

**Fig. 7:**
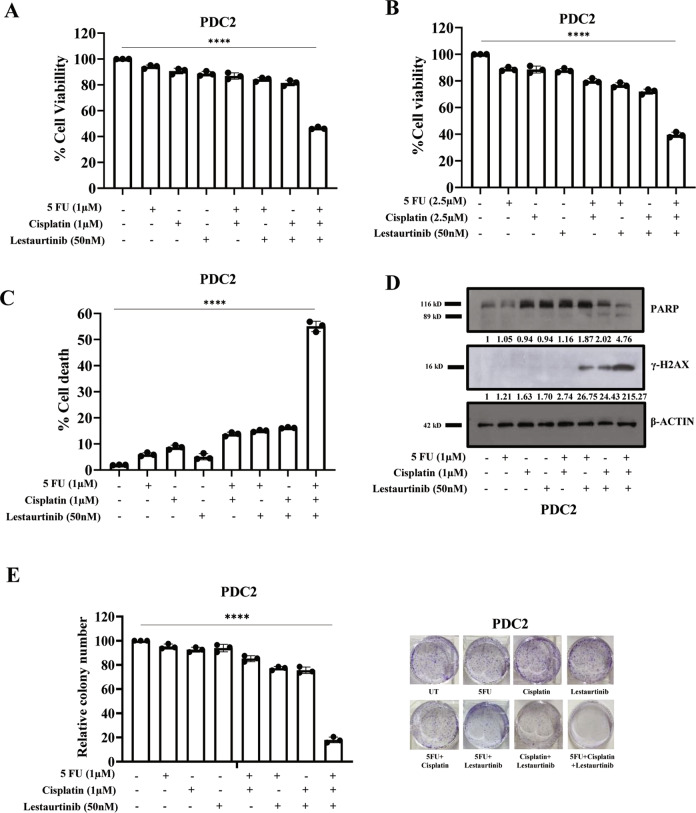
Evaluation of combinatorial anti-tumor effect of low dose of cisplatin, 5FU and lestaurtinb in TPF resistant patient derived cells (PDC2). **A**, **B** PDC2 cells were treated with indicated concentrations of cisplatin, 5FU and lestaurtinib for 48 h and cell viability was measured by MTT assay (*n* = 3 and, ****P < 0.0001 by 2-way ANOVA). **C** PDC2 cells were treated with indicated concentrations of cisplatin, 5FU and lestaurtinib for 48 h after which cell death was determined by annexin V/7AAD assay using flow cytometer. Bar diagrams indicate the percentage of cell death (early and late apoptotic) with respective treated groups (Mean ± SEM, *n* = 3 by Two-way ANOVA, ****P < 0.0001). **D** PDC2 cells were treated with indicated concentrations of cisplatin, 5FU and lestaurtinib for 48 h and immunoblotting (*n* = 3) was performed with indicated antibodies. **E** PDC2 cells were treated with indicated concentrations of cisplatin, 5FU, lestaurtinib for 12 days and colony forming assays were performed as described in method section. Left panel: Bar diagram indicate the relative colony number (*n* = 3 and, ****P < 0.0001 by 2-way ANOVA). Right panel: representative photographs of colony forming assay in each group.

From the screening data, we observed that MINK1 expression is elevated in chemoresistant OSCC and genetic inhibition of the same sensitizes drug resistant lines to 5FU induced cell death. Hence, MINK1 can be a potential therapeutic target to overcome chemoresistance in OSCC. Very limited information on the inhibitors of MINK1 is available in the literature. Hence, we looked for the potential MINK1 inhibitors in the international union of basic and clinical pharmacology (IUPHAR) database, where a screen of 72 inhibitors against 456 human kinases binding activity is provided. Among the potential twelve MINK1 inhibitors, we tested the MINK1 inhibitory activity of three inhibitors i.e., staurosporine, pexmetinib and lestaurtinib. The kinase assay data suggest that lestaurtinib and pexametinib have highest inhibitory activity for MINK1 (Fig. 5A). The 50% MINK1 inhibitory activity was observed at concertation of 100 nM in case of lestaurtinib and 10 µM for pexmetinib (Fig. 5B). Next, cell viability assay was performed to select a dose of lestaurtinib and pexmetinib that does not affect cell viability when treated alone (viability >80%) in 5FU resistant OSCC lines (Fig. 5C, D). Further, the cell viability, spheroid assay and cell death data suggest that the selected sub lethal dose of lestaurtinib (50 nM) and pexmetinib (500 nM) can efficiently restore 5FU mediated cell death in chemoresistant OSCC lines and PDC2 (Figs. 5E, F and S11A). The IC50 value of 5FU in H3575FUR is 20.49 μM, however combination of lestaurtinib (50 nM) decreases the IC50 value to 4.82 μM and combination of pexmetinib (500 nM) lowers the IC50 value of 5FU to 7.08 µM (Fig. 5E). As lestaurtinib, with a much lower concentration (50 nM) as compared to pexmetinib (500 nM) sensitizes 5FU to chemoresistant cells, from here on lestaurtinib was considered for rest of the study. Enhanced expression of p-H2AX and cleaved PARP was observed only in combination group with lestaurtinib and 5FU indicating programmed cell death (Figs. 5G and S11B). Boyden chamber assays data suggest that combinatorial treatment of lestaurtinib and 5FU significantly reduces the relative number of migrated cells (Fig. S11C). In harmony to the observation made by knockout of MINK1 in chemoresistant cells, we also found that lestaurtinib significantly decreased the phosphorylation of MDM2(Ser166) and AKT(Ser473) and elevated the expression of p53 in chemoresistant cells (Fig. 5H). Further, we found that lestaurtinib failed to sensitize 5FU mediated cell death in MINK1 knocked out 5FUR lines (Fig. 5I), which suggests that lestaurtinib conferred 5FU sensitivity by inhibiting MINK1 kinase activity. To check the in vivo efficacy of this novel combination, nude mice xenograft model was performed using patient derived cells (PDC2). The in vivo data suggest that the combination of lestaurtinib (20 mg/kg) and 5FU (10 mg/kg) profoundly reduced the tumor burden as compared to treatment with either of the single agents (Fig. 6A–C). Immunohistochemistry data suggest significant reduction in CD44 and Ki67 expression along with increased expression of cleaved caspase 3 in combination group (Fig. 6D). Finally, we performed combinatorial anti-tumor effect of non-cytotoxic extremely low dose of cisplatin (1 μM), 5FU (1 μM) and lestaurtinib (50 nM) in PDC2. The cell viability, cell death, western blotting and colony forming assay data suggest significantly higher cell death in cisplatin, 5FU and lestaurtinib combinatorial group, as compared to any other possible combinatorial group, i.e. 5FU and lestaurtinib or cisplatin and lestaurtinib or cisplatin and 5FU (Fig. 7).

## Discussion

It is well known that kinases play key role in various processes of carcinogenesis and kinase inhibitors are established as potential anti-tumor agents. In this study, for the first time, we have performed a kinome screening in drug resistant cancer cells to explore the potential kinase(s) those mediate 5FU resistance in OSCC. From the primary and secondary kinome screening, MINK1 was found to be the top ranked kinase that can be targeted to re-sensitize drug resistant cells to 5FU. Overall, MINK1 is known to regulate cell senescence, cell motility and migration. However, the potential role of MINK1 in modulating chemoresistanace is still unknown. Here in this study, we found the novel function of MINK1 by which it regulates 5FU resistance in OSCC.

To understand the mechanism by which MINK1 regulates 5FU resistance, we performed a high-throughput phosphorylation profiling in 5FUR cells stably expressing MINK1sgRNA. From this study, we found p- p53 (Ser33) and p-53(Ser15) to be significantly up-regulated in MINK1KO cells and p-AKT (Ser473) and p-MDM2 (Ser166) were found to be down-regulated in MINK1KO cells as compared to MINK1WT cells. The tumor suppressor p53 is phosphorylated at various amino acids by different kinases, which tightly regulates its stability [14]. It is well known that MDM2 (a E3 ubiquitin ligase) acts as a negative regulator of p53. MDM2 forms a complex with p53 and facilitates the recruitment of ubiquitin molecules for its degradation [15]. Earlier, it was established that insulin induced activated AKT (Ser473) phosphorylates MDM2 at Ser 166 and Ser 186, which can lead to MDM2 mediated proteasomal degradation of p53 in cytoplasm as well as in nucleus [16, 17]. These events lead to blocking of p53-mediated transcription of genes, those generally involve in apoptosis, cell cycle regulation and senescence. Further, p53 is phosphorylated at Ser15 by ATM, DNA-PK and ATR in response to DNA damage [18–20]. Hence, phosphorylation of Ser15 and Ser33 leads to activation and stabilization of p53 as they attenuate the MDM2 mediated degradation of p53 [21, 22]. Overall, in this study we found that MINK1 regulates the expression of p53 through activation of AKT which in turns triggers p-MDM2 (Ser 166) (Fig. 6E).

Jin et al. 2018 performed a kinome screening in cisplatin resistant cells to explore the potential kinases those confer cisplatin resistance in HNSCC. The data suggests that microtubule-associated serine/threonine-protein kinase 1 (MAST1) mediates cisplatin resistance in HNSCC by phosphorylating MEK1, triggering cRaf-independent activation of MEK1, which led to down regulation of BH3 only protein BIM. Jin et al. 2018 also found that lestaurtinib to be a potent inhibitor of MAST1. Lestaurtinib successfully restores the cisplatin induced cell death in cisplatin resistant cells [6]. Lestaurtinib not only inhibits MAST1 activity but also known as an inhibitor of JAK2, Trk and FLT3 [23, 24].

The most common chemotherapy regimen for OSCC is the combination of cisplatin, 5FU and Docetaxel (TPF). To achieve a better clinical outcome in OSCC, it is prerequisite that single agent targets both cisplatin and 5FU drug resistance. Earlier, it is known that lestaurtinib can inhibit MAST1 activity and can rewire cisplatin resistance in HNSCC. In this study we found that lestaurtinib can inhibit MINK1 and can rewire 5FU resistance. Hence, lestaurtinib is a potential kinase inhibitor which may have better outcome in overcoming TPF resistance in OSCC. In harmony to our hypothesis, we found that combination of extremely low dose of cisplatin (1 μM), 5FU (1 μM) and lestaurtinib (50 nM) can overcome chemoresistance in OSCC (Fig. 7). Currently lestaurtinib alone or in combination with other chemotherapy drug is under clinical investigation (phase II) for patients having AML with acceptable tolerance level [25].

Overall, our data suggests that MINK1 is a mediator of 5FU resistance in OSCC. Besides this, though we have demonstrated that MINK1 negatively regulates P53 through AKT/MDM2 axis in 5FU resistant OSCC, the 5FU specificity of this MINK1 driven signaling cascade remains to be fully elucidated. Further, genetic or pharmacological (Lestaurtinib) inhibition of MINK1 successfully resensitized chemo resistant lines to 5FU. This novel combination of 5FU and Lestaurtinib needs further clinical investigation.

## Materials and methods

### High content screening

1000 cells/ well were seeded with 3 replicates in black flat bottom 96 well plate (Thermo Scientific™ Nunc) and divided into two experimental groups, one without 5FU treatment and the other with 5FU treatment. A CRISPR based kinome-wide screening was performed using a lentiviral sgRNA library (LentiArray™ Human Kinase CRISPR Library, Thermo Fisher Scientific, Cat # M3775) that knocks out 840 kinase and kinase related genes individually with total number of 3214 sgRNA constructs. Transduction of lentiviruses (MOI:2) containing pooled sg RNAs (up to 4) targeting each of 840 genes along with positive and negative control lentiviruses into individual wells was carried out in presence of polybrene (8 µg/ml). At 48 h post transduction, selection with puromycin (0.5 µg/ml) was performed for next 2-3 days, followed by treatment of vehicle control or 5FU at sub lethal dose for 48 h. Finally, cells were stained with LIVE/DEAD™ Viability/Cytotoxicity Kit (Thermo Fisher Scientific Cat # L3224) and high content screening was performed using CellInsight CX7 High-Content Screening (HCS) Platform. The green fluorescence indicates the living cells and red fluorescence indicates dead cells. Images from 20 fields per well were acquired using 10X objective lens. Two different fluorescent channels (excitation wavelengths −488 nm and 561 nm) were used for acquiring images. Image analysis was performed using the HCS Studio software. A threshold value for each channel was set once and used for the entire screening. To identify the cells, segmentation was done. Some of the clumped and poorly segmented cells were excluded from further analysis on the basis of area, shape and intensity. On the basis of intensity, number of live and dead cells were counted and an objective mask (blue lines in the images) was created around each cell. For positive control, Cas9 over expressing cells were transduced with lentiviruses expressing sgRNA targeting human hypoxanthine phosphoribosyltransferase 1 (HPRT1) (LentiArray™ CRISPR Positive Control Lentivirus, human HPRT, Thermo Fisher Scientific Cat # A32829). HPRT1 knockout cells showed resistance to 6-thioguanine (6TG) induced cell death. For negative control, Cas9 over expressing cells were transduced with lentiviruses expressing gRNA with no sequence homology to any region of the human genome (LentiArray™ CRISPR Negative Control Lentivirus, Thermo Fisher Scientific, Cat # A32327). Both set of plates (treated with vehicle control or 5FU) were normalized to negative control sgRNA to have the % cell viability. Further, survival fraction was calculated by dividing the number of live cells in individual kinase knockout well treated with 5FU with the number of live cells in each kinase knockout well treated with vehicle control.

### In vitro MINK1 kinase activity assays

MINK1 Kinase Enzyme System (Promega Cat No# V3911) and ADP-Glo™ Kinase Assay (Promega Cat No#V9101) were procured to perform the in vitro kinase assay. Selected compounds (10 µM) were incubated at room temperature for 1 h with recombinant human MINK1 kinase along with substrate MBP and ATP to perform the kinase reaction using 1X kinase buffer. Further ADP-Glo™ Reagent was added at room temperature for 40 min to stop the kinase reaction and deplete the unconsumed ATP, hence leaving only ADP. Then Kinase detection reagent was added and incubated at room temperature for 30 min to convert ADP to ATP and introduce luciferase and luciferin to detect ATP. Finally, the luminescence was measured using VICTOR® Nivo™ Multimode Plate Reader (Perkin Elmer).

### Statistical analysis

All data points are presented as mean and standard deviation and Graph Pad Prism 9.0 was used for calculation. The statistical significance was calculated by 2-tailed Student’s *t* test, one-way variance (one-way ANOVA), Two-Way ANOVA and considered significant at *P* ≤ 0.05.

Detailed methods are provided in the Supplementary materials and methods section.

### Study approval

This study was approved by the Institute review Board and Human Ethics committees (HEC) of Institute of Life Sciences, Bhubaneswar (110/HEC/21) and All India Institute of Medical Sciences (AIIMS), Bhubaneswar (T/EMF/Surg.Onco/19/03). The animal related experiments were performed in accordance to the protocol approved by Institutional Animal Ethics Committee of Institute of Life Sciences, Bhubaneswar (ILS/IAEC-204-AH/DEC-20). Approved procedures were followed for patient recruitment and after receiving written informed consent from each patient, tissues samples were collected. Institutional biosafety committee (IBSC) approved all related experiments.

## Supplementary information

SUPPLEMENTARY MATERIALS
