# Supplementary material for: CRISPR-based kinome-screening revealed MINK1 as a druggable player to rewire 5FU-resistance in OSCC through AKT/MDM2/p53 axis
Source: Oncogene. 2022 Oct 1;41(45):4929–40. doi: 10.1038/s41388-022-02475-8 (PMC9630125; doi:10.1038/s41388-022-02475-8)
Supplement: Supplementary file 1 — SUPPLEMENTARY MATERIALS [file 41388_2022_2475_MOESM1_ESM.pdf]

Supplementary Materials and Methods

Cell culture: The human tongue OSCC lines **(**H357, SCC4 and SCC9) were obtained from Sigma Aldrich, sourced from European collection of authenticated cell culture. All OSCC cell lines were cultured and maintained in DMEM F12 supplemented with 10% FBS (Thermo Fisher Scientific), penicillin–streptomycin (Pan Biotech) and 0.5 ug/ml sodium hydrocortisone succinate. HEK 293T, HEK 293, MCF7 and HCT116 cells were obtained from ATCC and maintained in DMEM supplemented with 10% FBS and penicillin–streptomycin (Pan Biotech).

Generation of 5-Fluorouracil resistant cell lines: For establishment of 5FU resistant cell lines, human OSCC cell lines (H357, SCC4 and SCC9) were initially treated with 1µM (lower dose) of 5-Fluorouracil for a week and then the concentration of 5FU was gradually increased up to the IC50 value, i.e. 10 µM for H357, 15 µM for SCC4 and 7.5 µM for SCC9 within a span of 3 months. Parental cells were grouped as sensitive (H357 5FUS, SCC4 5FUS and SCC9 5FUS) and after a period of 8 months of 5FU treatment, they were termed as 5FU Resistant (H357 5FUR, SCC4 5FUR and SCC9 5FUR) cells.

Genomic Cleavage Detection Assay: The genomic cleavage efficiency was measured using the GeneArt® Genomic Cleavage Detection kit (Thermo Fisher Scientific, Cat # A24372). For this, cells were lysed and DNA was extracted, followed by PCR amplification of the region on genome, where Cas9 endonuclease introduced a cleavage, using specifically designed primers as described in the protocol of the kit. Further, the PCR products were denatured and allowed for random reannealing, so that mismatches are generated as a result of genomic insertions or deletions (indels) created by the cellular repair mechanisms following the cleavage induced by Cas9. These mismatches were subsequently detected and cleaved by Detection Enzyme and

then the resultant bands were analyzed by agarose gel electrophoresis. Primers used in this study for cleavage detection assay are mentioned in Table S4.

Lentivirus production and generation of stable MINK1 KO cell lines : LentiCas9-Blast was obtained from addgene (#52962) which is kindly deposited by Feng Zhang lab [1]. sgRNAs targeting MINK1 were cloned into pKLV2-U6gRNA5(BbsI)-PGKpuro2ABFP-W (Addgene #67974) vector as per the protocol mentioned in addgene, which is kindly deposited by Kosuke Yusa lab [2]. Respective lentiviruses were produced by transfection of LentiCas9-Blast or pKLV2-U6gRNA5(BbsI)-PGKpuro2ABFP-W plasmid along with packaging plasmid psPAX2 and envelop plasmid pMD2G into HEK293T cells as described in Shriwas et al [3]. Further 5FUR cells were infected with MINK1 sgRNA lentivirus using polybrene (8µg/ml) followed by puromycin (up to 5µg/ml) selection. After a week cells were picked up and seeded in 96 well plates with 1 cell/well dilution. Confirmations were done by western analysis. All sgRNA sequences used in this study are mentioned in Table S4.

Lentivirus production and generation of stable MINK1 KD cell lines: pLKO.1vector was obtained from addgene (Cat #10878), which is kindly deposited by David Root lab [4]. shRNAs targeting MINK1 were cloned into pLKO.1vector as per the protocol mentioned in addgene. Lentivirus was produced by transfection of pLKO.1 plasmid along with packaging plasmid psPAX2 and envelop plasmid pMD2G into HEK293T cells. Further 5FUR cells were infected with MINK1 shRNA lentivirus using polybrene (8µg/ml) followed by puromycin (up to 5µg/ml) selection. After 15 days colonies were picked up and confirmations were done by western analysis. All shRNA sequences used in this study are mentioned in Table S4.

Phospho-Protein Profiling: For performing the Phospho Explorer Antibody Array (Full Moon Biosystems Cat# PEX10), 5X106 number of H3575FUR MINK1WT and H3575FUR MINK1KO cells were seeded and lysates were isolated, after which the protein samples were

labelled with biotin as described by the manufacturer. Biotinylated protein samples were further blocked in skim milk and were subjected to coupling with 1318 number of antibodies present on the array slides. Then for the detection of expression of phospho proteins, Cy3-streptavidin (Sigma, Cat#S6402) was added. Array slides were scanned at Fullmoon Biosystems array scanning service and the image analysis was done using ImageJ software.

RT-PCR and Real Time Quantitative PCR: RNA mini kit (Himedia, Cat# MB602) was used to isolate total RNA as per manufacturer's instruction and quantified by Nanodrop. Verso cDNA synthesis kit (ThermoFisher Scientific, Cat # AB1453A) was used to synthesize c-DNA by reverse transcription PCR using 300 ng of RNA. qRT-PCR was carried out using SYBR Green master mix (Thermo Fisher scientific Cat # 4367659). GAPDH was used as a loading control. The primers (oligos) sequence used for qRT-PCR in this study are listed in Table S4.

Immunoblotting: Immunoblotting was performed by loading equal amounts of cell lysates as described earlier [5]. In this study, primary antibodies used were against β-actin (Sigma, Cat#A2066), Cas9 (CST, Cat #14697S), MINK1 (Sigma, Cat# HPA056296, Invitrogen, Cat# PA5-28901), P53 (CST, Cat #2527T), p-P53(Ser15) (CST, Cat #9284T), p-P53(Ser33) (CST, Cat #2526), PARP (CST, Cat #9542L), p^{s-139}-H2AX (CST, Cat # 9718S), AKT (CST, Cat #9272S), pAKT(Ser473) (CST, Cat #4058S), MDM2 (Santa Cruz, Cat #sc965), pMDM2(ser166) (Abcam, Cat # ab131355), TIGAR (Abcam, Cat # Ab37910), P21(CST, Cat # 2947S), NOXA (Imgenex, Cat # IMG-349A), Thymidylate synthase (Novus, Cat # NBP2-34441H). The quantification of band intensity in all immunoblots (n = 3) was performed using ImageJ software (NIH). The mean value of band intensity is indicated under the immunoblots. The intensity for each blot was calculated by normalizing with the intensity of β-actin blot (internal control).

Assessment of cell viability: Cell viability was measured by 3-(4, 5-dimethylthiazol-2-yl)-2, 5-diphenyltetrazolium bromide (MTT; Sigma-Aldrich) assay as per manufacturer's instruction.

Colony formation assay: Colony formation assay was performed as described in Shriwas et al [6].

Annexin-V PE/7-AAD Assay: Apoptosis and cell death assay was performed by using Annexin V Apoptosis Detection Kit PE (eBioscience™, USA, Cat # 88-8102-74) as described earlier [6] and cell death was monitored using a flow cytometer (BD FACS Fortessa, USA).

Tumorsphere formation assay: Tumorsphere assay was performed as described in Mohapatra et al [7]

Immunofluorescence: The cells were seeded on lysine coated coverslip and cultured for overnight. On next day, cells were treated with 5FU for 48 hrs followed by 4% formaldehyde fixation for 15 mins. Next, cells were permeabilized with 1 × permeabilization buffer (eBioscience 00-8333-56) for 45 mins, followed by blocking with 3% BSA for 1 h at room temperature. After which, the cells were incubated with primary antibody overnight at 4 °C, washed three times with PBST pH 8.0 followed by 1hr incubation with Goat anti–Rabbit IgG(H+L) secondary Antibody, Alexa Fluor® 488 conjugate (Invitrogen, Cat #A -11008). After washing three times with PBST pH 8.0, cells were mounted with DAPI (Slow Fade ® GOLD Antifade, Thermo Fisher Scientific, Cat # S36938). Images were captured using a confocal microscopy (LEICA TCS-SP8). Anti $p^{s\text{-}139}$-H2AX (CST, Cat # 9718S) primary antibody was used for this study.

Immunohistochemistry: Immunohistochemistry of formalin fixed paraffin-embedded samples (OSCC patients' tumors and Xenograft tumors from mice) were performed as described previously [7]. Antibodies against MINK1 (Sigma, Cat# HPA056296, Invitrogen, Cat# PA5-28901), CD44 (NOVUS, Cat# NBP1-31488), Cleaved Caspase-3 (CST, Cat#

9661S), Ki67 (Vector, Cat #VPRM04) were used for IHC. Images were obtained using Leica DM500 microscope. Q-score was calculated by multiplying percentage of positive cells with staining (P) and intensity of staining (I). P was determined by the percentage of positively stained cells in the section and I was determined by the intensity of the staining in the section i.e. strong (value=3), intermediate (value=2), weak (value=1) and negative (value=0).

Transient transfection and overexpression of MINK1 and myr-AKT in MINK1 KD cell lines: pDONR223-MINK1 (Plasmid #23522) was procured from addgene followed by transfer of the insert into pLenti CMV/TO Puro DEST (670-1) (Addgene, Cat#17293) destination vector by gateway cloning method using Gateway LR Clonase II Plus Enzyme mix (Invitrogen, Cat# 1756069). These plasmids were kindly deposited by William Hahn, David Root labs [8] and Eric Campeau, Paul Kaufman labs [9] in addgene. Further, MINK1 knockdown cells, stably expressing shRNA#1 targeting 3' UTR of MINK1 mRNA, were transiently transfected with pLenti CMV/TO Puro DEST-MINK1 using the ViaFect transfection reagent (Promega Cat# E4982). The transfection efficiency was confirmed by immunoblotting against Anti-MINK1. pLNCX myr HA Akt1 (Addgene, Cat #9005) was used for transient overexpression of constitutively activated AKT. The myr HA Akt1 vector was kindly deposited to Addgene by Sellers WR lab [10].

Generation of stable cell lines overexpressing MINK1 WT and MINK1 K54R kinase mutant: MINK1 WT and MINK1 K54R (kinase dead mutant) genes were synthesized, cloned into puc19 vector and procured from GenScript Biotech Corporation, further subcloned into pSilencer™ 4.1-CMV puro vector. Next, 5FU sensitive cells were stably transfected with either pSilencer™ 4.1-CMV puro-MINK1WT or pSilencer™ 4.1-CMV puro-MINK1 K54R followed by puromycin selection (up to 5µg/ml). After 15 days colonies were picked up and over expression was confirmed by western analysis.

OSCC patient sample: Loco regionally advanced OSCC samples were collected. Neoadjuvant chemotherapy has been prescribed before surgery and/or radiotherapy. The three-drug combination of TPF is having highest response (TAX 324). After chemotherapy (CT) the response is evaluated as per RECIST criteria (Response evaluation criteria in solid tumors) by clinical and radiological evaluation. After chemotherapy the patient can be grouped as complete response (CR), Partial Response (PR), stable disease (SD) or Progressive Disease (PD). If there was no evidence of malignancy, then it was diagnosed as complete response (CR). If the target lesions had decreased more than equal to 30% of the sum of the longest diameter, then it was diagnosed as partial response (PR). If there was no sign of either CR or PR, then it was called stable disease, and if the target lesions had increased more than or equal to 20% of the sum of the longest diameter, then it was called PD (progressive disease). As the patients showing CR and PR have responded to the CT they are categorized as Responders and the patients with stable disease or PD with almost no response to CT are categorized as Non-Responders. Human Ethics Committee (HEC) of the Institution of Life Sciences approved all patient-related studies, and informed consent was obtained from all patients. Study subject details with treatment modalities are presented in Table S3a and b.

Patient Derived Xenograft: BALB/C-nude mice (6-8 weeks, male, NCr-Foxn1nu athymic) were purchased from Hylasco Bio-Technology Pvt. Ltd. For xenograft model early passage of patient-derived cells (PDC2) established from chemo non-responder patient (treated with TPF without having any response) was considered. Two million cells were suspended in phosphate-buffered solution-Matrigel (1:1, 100 μl) and transplanted into upper flank of mice. The PDC MINK1WT cells were injected in right upper flank and PDC MINK1KO cells were injected in the left upper flank of same mice. These mice were randomly divided into 2 groups (n=5) once the tumors reached a volume of 50 mm^3 and injected with vehicle control or 5FU (10mg/kg)

intraperitonially twice a week. In another experimental set up, PDC2 WT cells were injected in right upper flank of mice. These mice were randomly divided into 4 groups (n=5) after the tumors have reached a volume of 50 mm^3 and injected with vehicle control, 5FU (10mg/kg), Lestaurtinib (20mg/kg) or 5FU (10mg/kg) and Lestaurtinib (20mg/kg) respectively in each individual group, intraperitonially twice a week. Tumor size was measured using digital Vernier caliper twice a week until the completion of experiments. Tumor volume was determined using the following formula: Tumor volume (mm^3) = (minimum diameter)^2 × (maximum diameter)/2.

Zebrafish xenograft: The experimental protocols used for this work were approved by the institutional animal ethical review committee (ILS/IAEC-214-AH/APR-21). PDC2 control and MINK1 knockdown stable cells were suspended in individual tubes at a density of 1x 10^6 cells/ml in normal media followed by addition of 5µl of the cell-labeling solution (Vybrant™ DiI Cell-Labeling Solution Catalog number: V22885) and mixed well by gentle pipetting. The cells were incubated for 20 minutes at 37°C to obtain uniform labeling and then centrifuged at 1100 rpm for 5 minutes at RT. The supernatant was removed and cells were resuspended gently in 1ml of warm (37°C) media and centrifuged at 1100 rpm for 2 minutes for the removal of extra dye and the wash step was repeated twice. The Dil stained cells were resuspended at final density 200 cells/nl and ~400 cells were microinjected (Femtojet microinjector) into perivitelline space of 48 hpf embryos of zebrafish (*Danio rerio*) [Tg(fli1:EGFP)] for the development of tumor. The images of zebrafish embryos were captured using a fluorescence stereomicroscope (Leica MZ16) on the day of injection (Day 0), followed by 5FU treatment (500µM) 3 days post injection (Day 3) and then final imaging 5 days after injection (Day 5). The tumor growth was assessed by an increase or decrease in fluorescence intensity on the 5th day compared to the day of injection. The quantitation of fluorescence intensity was performed

using ImageJ software and represented as mean fluorescent intensity where day 0 readings was taken as baseline.

Flowcytometric analysis of Thymidylate synthase: Percentage of TS positive cells were analyzed using a flow cytometer (Beckman Coulter CytoFLEX S). Briefly, 5×10^4 cells were fixed in 4% formaldehyde followed by permeabilization and blocking in 3% BSA. Then cells were incubated with IgG isotype antibody or TS primary antibody (Novus, Cat # NBP2-34441H),followed by incubation with Goat anti–Rabbit IgG(H+L) secondary Antibody, Alexa Fluor® 488 conjugate (Invitrogen, Cat #A -11008). Data were acquired using FITC-A (X-axis) verses side scatter SSC-A (Y-axis) and analyzed using CytExpert software tool.

Trans-well migration assay: For trans-well migration assay, MINK1WT and MINK1 KO chemoresistant cells were treated with vehicle control or 5FU (10 μM, 48hrs). In another experimental set up, 5FU resistant cells were treated with vehicle control, 5FU (7.5 μM), Lestaurtinib (50 nM), or 5FU (7.5 μM) and Lestaurtinib (50 nM). After treatment, cells were seeded at a density of 1×10^4 cells in the upper chamber of 24-well trans-well system in 250 μL of serum free DMEM-F12 medium. 750 μL of DMEM-F12 Medium supplemented with 10% serum, used as a chemo-attractant, was added in the lower chamber. After 24 hrs of incubation, the cells inside the upper chamber were scrubbed with a cotton swab, whereas the migrated cells were fixed and stained with crystal violet, followed by quantification of migration by manual counting using a microscope.

***In vitro* scratch assay:** *In vitro* scratch assay was performed as described earlier [5].

References

1 Sanjana NE, Shalem O, Zhang F. Improved vectors and genome-wide libraries for CRISPR screening. *Nat Methods* 2014; 11: 783-784.

2 Tzelepis K, Koike-Yusa H, De Braekeleer E, Li Y, Metzakopian E, Dovey OM *et al*. A CRISPR Dropout Screen Identifies Genetic Vulnerabilities and Therapeutic Targets in Acute Myeloid Leukemia. *Cell Rep* 2016; 17: 1193-1205.

3 Shriwas O, Arya R, Mohanty S, Mohapatra P, Kumar S, Rath R *et al*. RRBP1 rewires cisplatin resistance in oral squamous cell carcinoma by regulating Hippo pathway. *Br J Cancer* 2021; 124: 2004-2016.

4 Moffat J, Grueneberg DA, Yang X, Kim SY, Kloepfer AM, Hinkle G *et al*. A lentiviral RNAi library for human and mouse genes applied to an arrayed viral high-content screen. *Cell* 2006; 124: 1283-1298.

5 Samal SK, Routray S, Veeramachaneni GK, Dash R, Botlagunta M. Ketorolac salt is a newly discovered DDX3 inhibitor to treat oral cancer. *Scientific reports* 2015; 5: 9982.

6 Shriwas O, Priyadarshini M, Samal SK, Rath R, Panda S, Das Majumdar SK *et al*. DDX3 modulates cisplatin resistance in OSCC through ALKBH5-mediated m(6)A-demethylation of FOXM1 and NANOG. *Apoptosis* 2020; 25: 233-246.

7 Mohapatra P, Shriwas O, Mohanty S, Ghosh A, Smita S, Kaushik SR *et al*. CMTM6 drives cisplatin resistance by regulating Wnt signaling through the ENO-1/AKT/GSK3beta axis. *JCI Insight* 2021; 6.

8 Johannessen CM, Boehm JS, Kim SY, Thomas SR, Wardwell L, Johnson LA *et al*. COT drives resistance to RAF inhibition through MAP kinase pathway reactivation. *Nature* 2010; 468: 968-972.

9 Campeau E, Ruhl VE, Rodier F, Smith CL, Rahmberg BL, Fuss JO *et al*. A versatile viral system for expression and depletion of proteins in mammalian cells. *PloS one* 2009; 4: e6529.

10 Ramaswamy S, Nakamura N, Vazquez F, Batt DB, Perera S, Roberts TM *et al*. Regulation of G1 progression by the PTEN tumor suppressor protein is linked to inhibition of the phosphatidylinositol 3-kinase/Akt pathway. *Proc Natl Acad Sci U S A* 1999; 96: 2110-2115.

Supplementary Figure 01

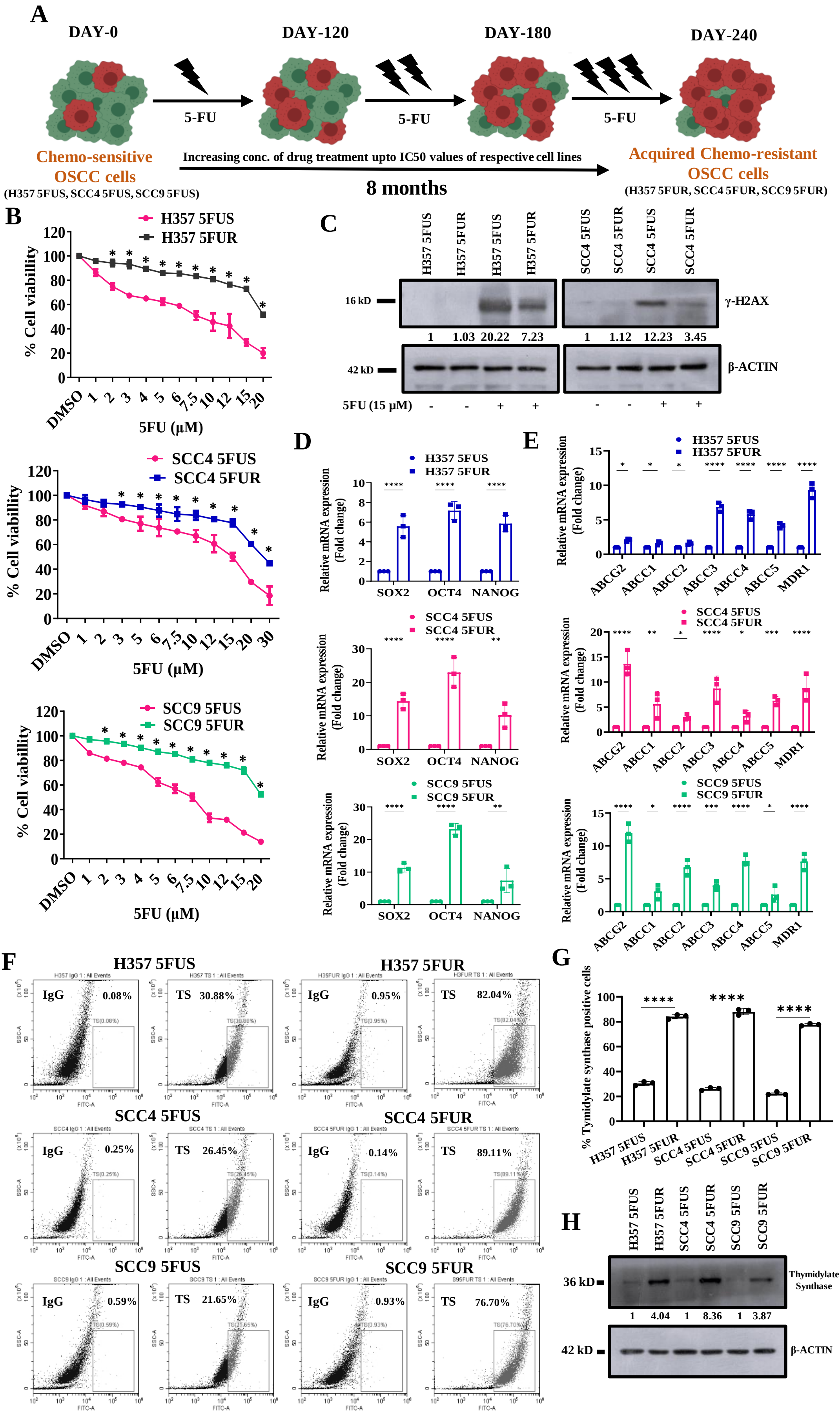

Supplementary Figure 02

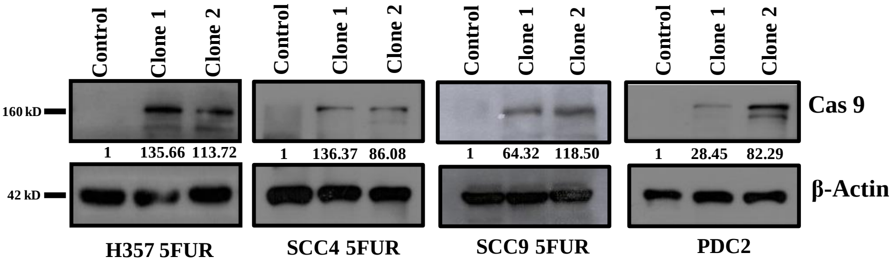

Supplementary Figure 03

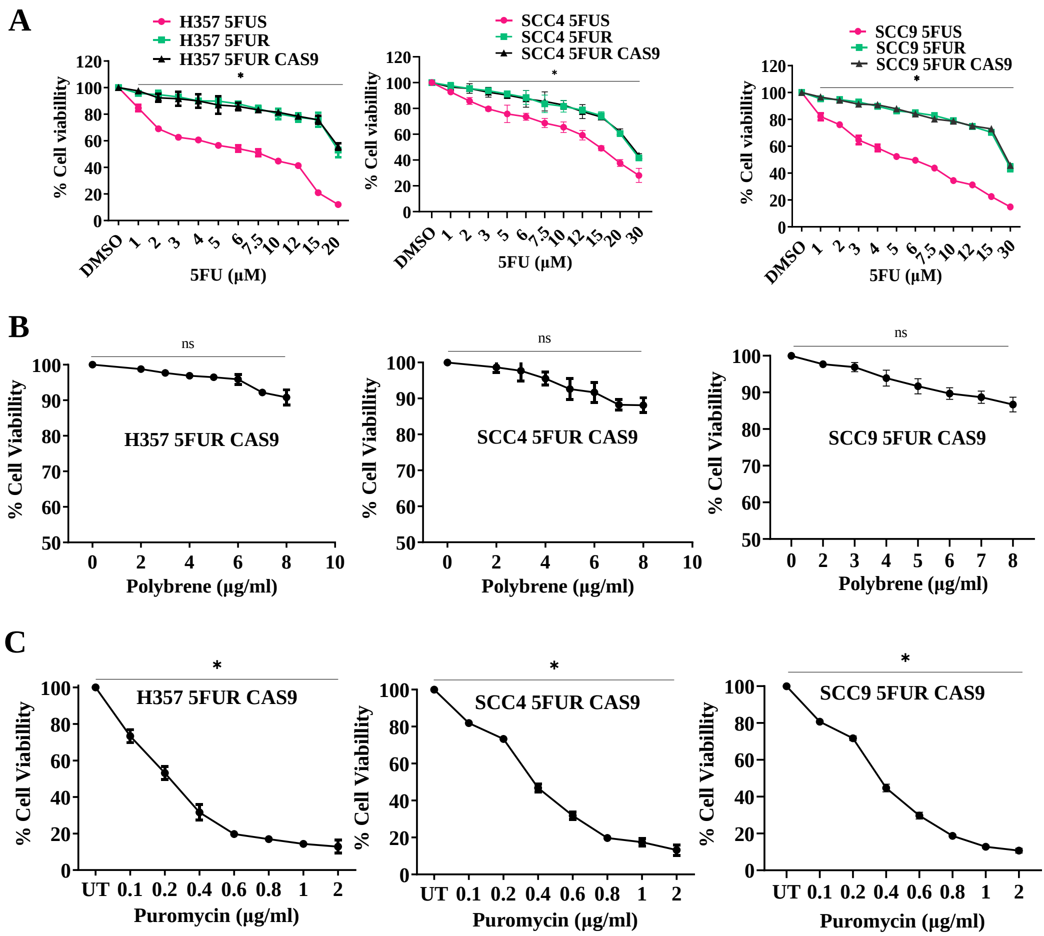

Supplementary Figure 04

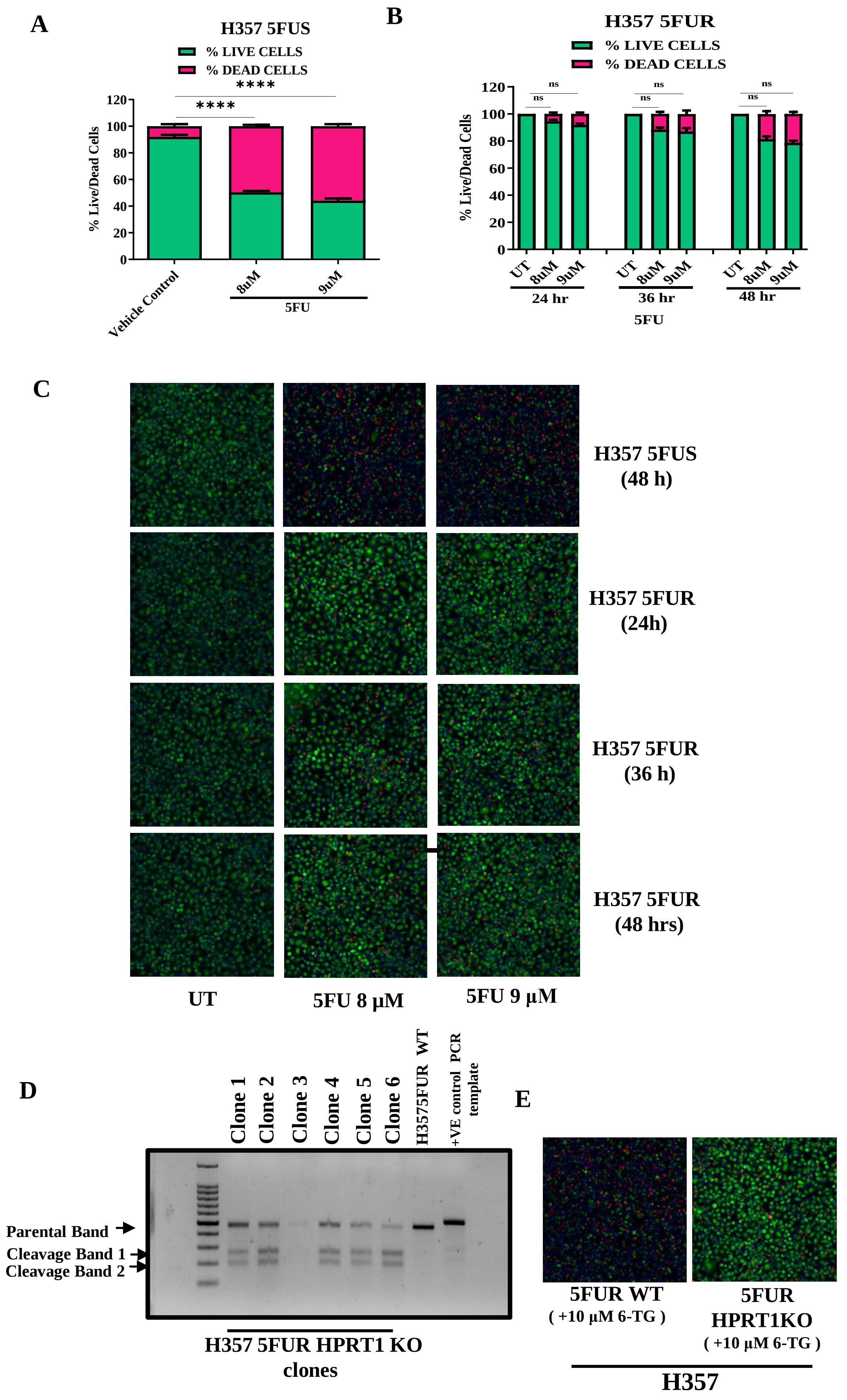

Supplementary Figure 05

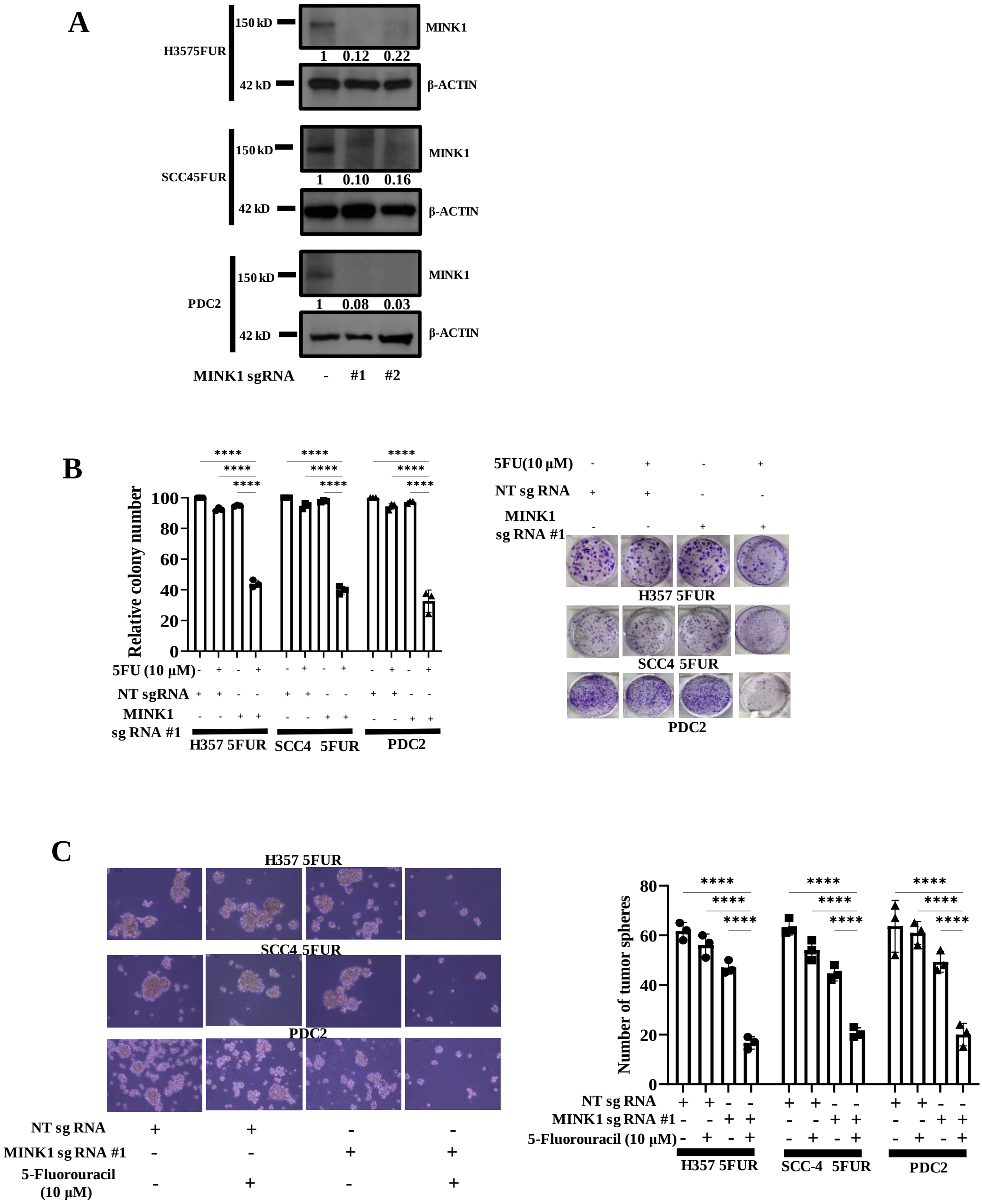

Supplementary Figure 06

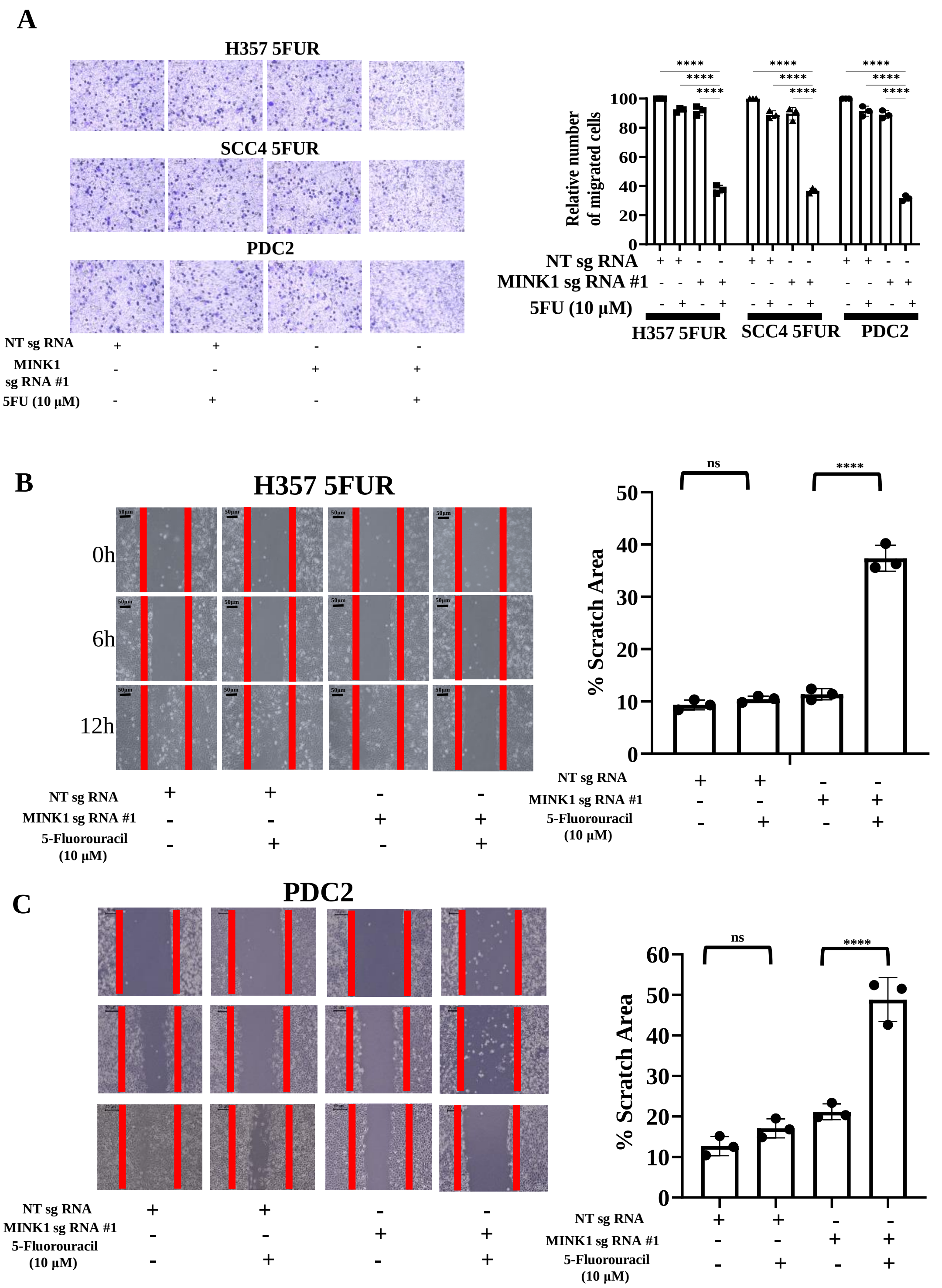

Supplementary Figure 07

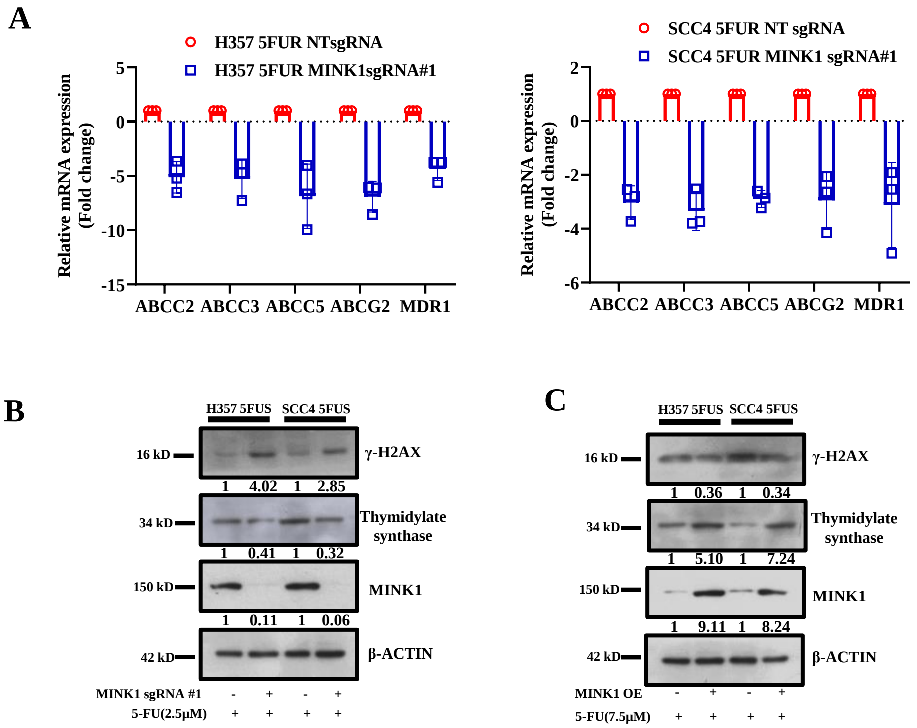

Supplementary Figure 08

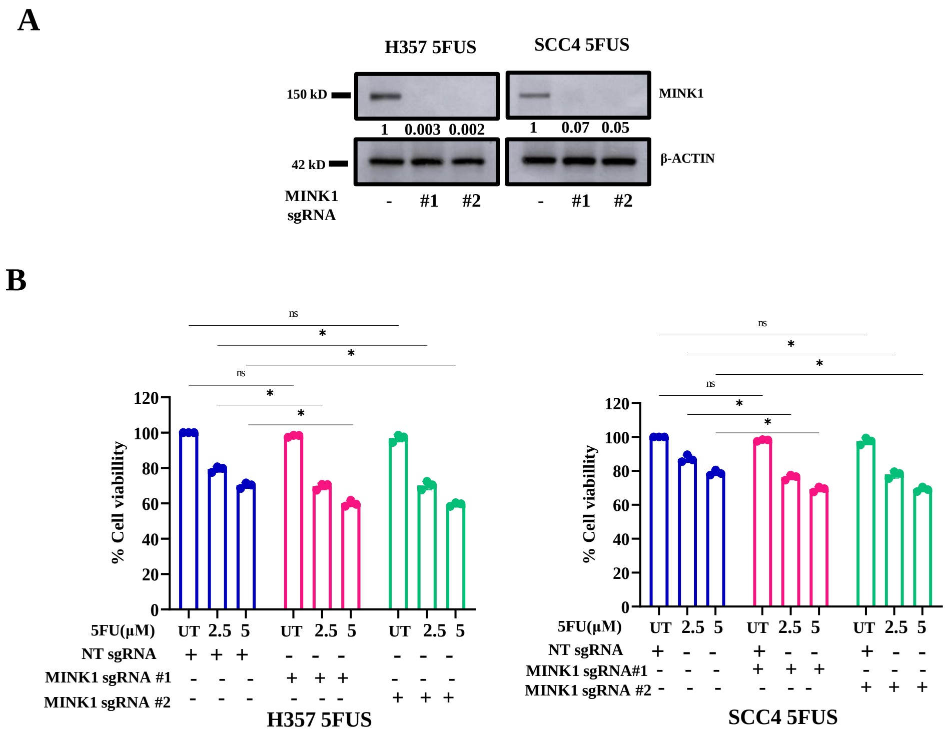

Supplementary Figure 09

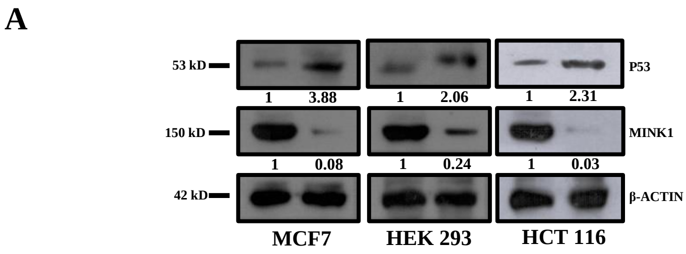

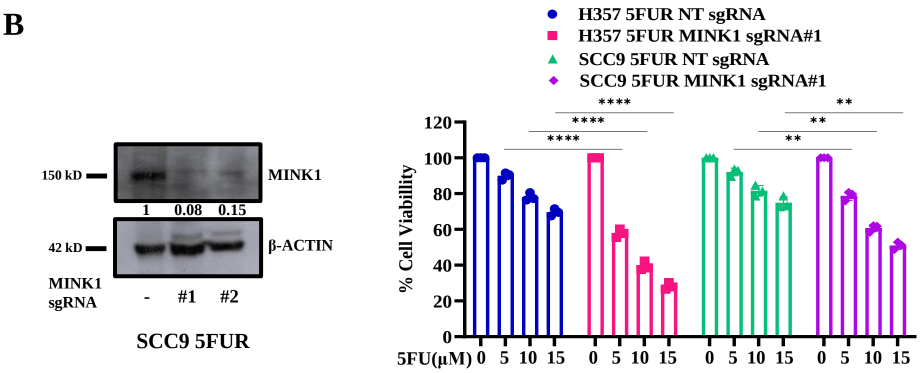

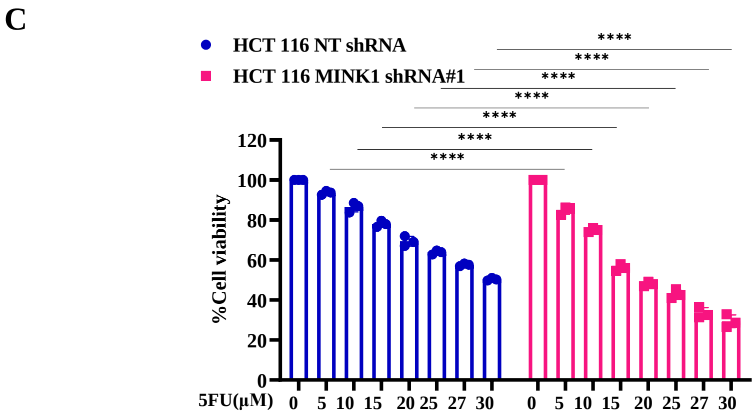

Supplementary Figure 10

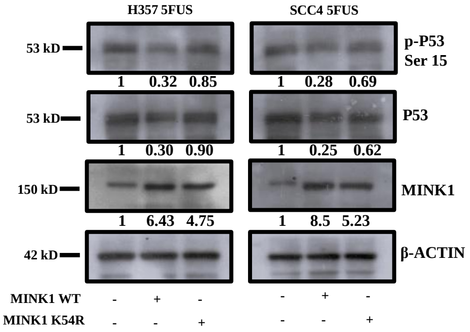

Supplementary Figure 11

A

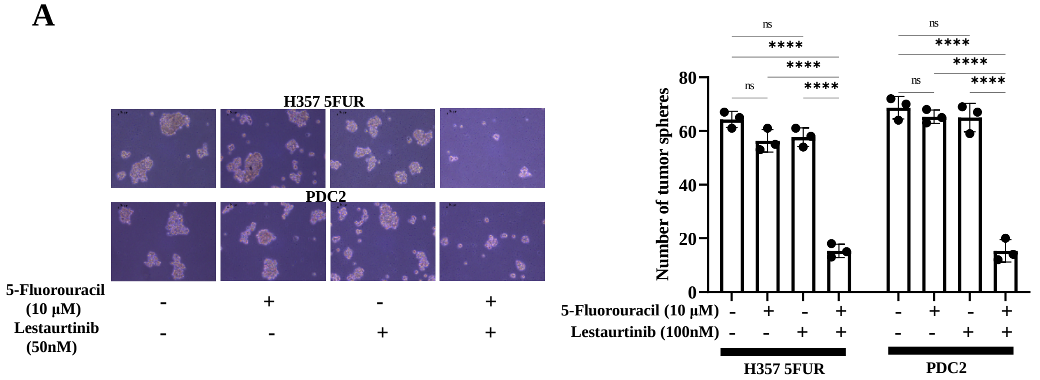

B

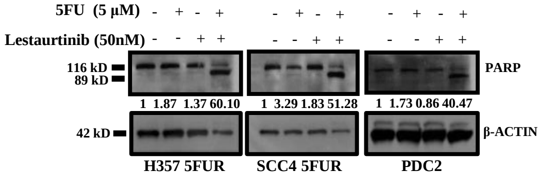

C

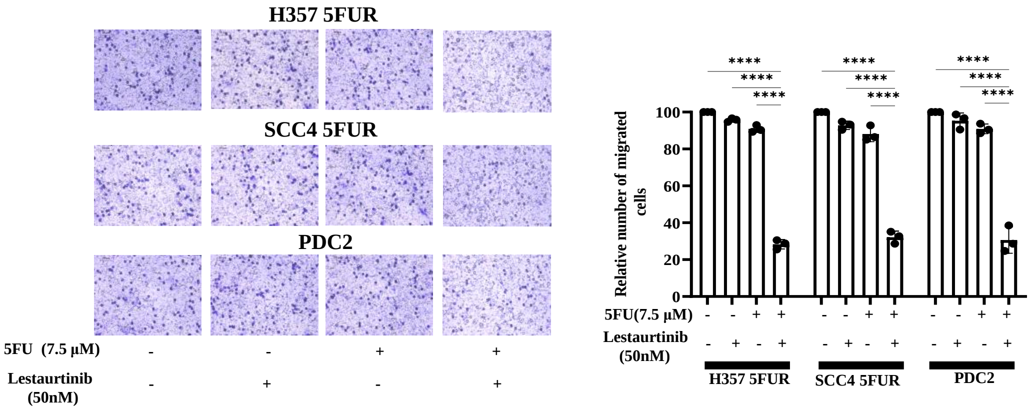

Figure S1: Characterization of sensitive and 5FU resistant OSCC lines: A) Schematic presentation of approach for establishing 5FU resistant OSCC lines **B)** Sensitive and 5FU resistant pattern (5FUS and 5FUR) of H357, SCC4 and SCC9 cells were treated with indicated concentrations of 5FU for 48h and cell viability was determined by MTT assay (n=3, *: $P < 0.05$). 2-way ANOVA. **C)** Indicated 5FU sensitive and resistant cells were treated with 5FU (15µM) for 48 hrs, after which lysates were collected and immunoblotting (n=3) was performed with indicated antibodies. **D-E)** RNA was isolated from Sensitive and 5FU resistant pattern (5FUS and 5FUR) of H357, SCC4 and SCC9 cells and relative mRNA (fold change) expression of indicatedgenes was analyzed by qRT-PCR (mean ± SEM, n = 3), 2-way ANOVA, *$P < 0.05$, **$P < 0.01$, ***$P < 0.001$, ****$P < 0.0001$. **F-G)** 5FU sensitive and resistant OSCC lines were immunostained with thymidylate synthase antibody and flow cytometry was performed to analyze thymidylate synthase positive cells from indicated lines as described in method section. Bar diagrams indicate the percentage of TS positive cells from indicated lines (Mean ±SEM, n=3, Two-way ANOVA, ****$P < 0.0001$) **H)** Lysates were collected from indicated cells and immunoblotting (n=3) was performed with indicated antibodies.

Figure S2: Overexpression of Cas9 in 5FU resistant OSCC lines and PDC: Indicated cells were stably transfected with LentiCas9-Blast and 2 clones were selected from indicated cell lines. Lysates were collected from indicated cells and immunoblotting (n=3) was performed with indicated antibodies.

Figure S3: Characterization of Cas9 overexpressing OSCC lines regarding 5FU resistance, polybrene tolerance and puromycin sensitivity: A) Indicated sensitive, 5FU resistant, 5FU resistant Cas9 overexpressing cells were treated with indicated concentrations of 5FU for 48h and cell viability was determined by MTT assay (n=3 and *$P < 0.05$ by 2-way ANOVA). **B)** Indicated 5FU resistant Cas9 overexpressing cells were treated with indicated concentrations of polybrene

for 48h and cell viability was determined by MTT assay (n=3 and *$P < 0.05$ by 2-tailed Student's t test). **C)** Indicated 5FU resistant Cas9 overexpressing cells were treated with indicated concentrations of puromycin for 48h and cell viability was determined by MTT assay (n=3 and *$P < 0.05$ by 2-tailed Student's t test).

Figure S4: Validation of 5FU resistance and optimization of kinome screening conditions using high content analyzer: A-B) 5FU sensitive and resistant patterns of H357 cells were treated with indicated concentrations of 5FU for the indicated time points, after which cell viability was measured in high content analyzer using a live/dead cell imaging kit (n=3 and ****$P < 0.0001$ by 2- way ANOVA). **C)** The representative fluorescent images acquired from high content analyzer with indicated treated groups in indicated cells. **D)** Different HPRT1 KO clones in H357 5FUR cells were subjected to genomic cleavage detection assay as described in materials and methods section. **E)** The representative fluorescent images acquired from high content analyzer with indicated treated groups in indicated cells.

Figure S5: Targeting MINK1 reduced cell proliferation and stemness in chemoresistant OSCC: A) MINK1 knock out clones were generated using a lentiviral approach expressing 2 different sgRNAs (#1 and #2) in Cas9 overexpressing 5FU resistant lines and PDC2. Immunoblotting (n=3) was performed with indicated antibodies in indicated cells. Patient derived cells (PDC2) was established from tumor of TPF treated chemo-nonresponder patient. **B)** MINK1 KO and MINK1WT cells were treated with 5FU for 12 days and colony forming assays were performed as described in method section. Left panel: Bar diagram indicates the relative colony number (n=3 and **** $P < 0.0001$ by 2-way ANOVA). Right panel: representative photographs of colonyforming assay in each group. **C)** Left panel: Tumor spheroid assay was performed as described inMethods with indicated cells stably expressing NTSgRNA and MINK1sgRNA#1 followed by treatment with indicated concentration of 5FU for 5 days. At the end of the

experiment, spheroid photographs were captured using Leica DMIL microscope. Scale bar, 200 μm. Right panel: Number of tumor spheres formed from the experiment in C was counted. (n = 3), 2-way ANOVA, ****$P < 0.0001$.

Figure S6: MINK1 genomic ablation negatively affects tumor cell migration in chemoresistant OSCC: A) Indicated MINK1 WT and KO cells were treated with vehicle control or 5FU (10 μM) for 48h and were subjected to Boyden chamber assay as described in materials and methods section. Left panel: representative photographs of Boyden chamber assay in each group. Right panel: Bar diagrams indicate the relative number of migrated cells (n=3 and ****$P < 0.0001$ by 2-way ANOVA). Scale bars: 50 μm. **B-C)** Indicated MINK1 WT and KO cells were treatedwith vehicle control or 5FU (10 μM) for 48h and were subjected to scratch assay as described in materials and methods section. Left panel: representative photographs of scratch assay in each group. Right panel: Bar diagrams indicate the percentage scratch area (n=3 and ****$P < 0.0001$by 2-way ANOVA). Scale bars: 50 μm.

Figure S7 MINK1 regulates ABC transporters and Thymidylate synthase: A-B) RNA was isolated from indicated cells and q-PCR has been performed with indicated genes. Relative gene expression in MINK1sgRNA was determined as compared to NTsgRNA. **C)** OSCC lines were stably transfected with MINK1 sgRNA and 5FU was treated for 48h, after which immunoblotting was performed with indicated antibodies. **D)** MINK1 was stably overexpressed in OSCC sensitive lines and indicated concentration of 5FU was treated for 48h after which immunoblotting was performed with indicated antibodies.

Figure S8: MINK1 deletion sensitizes OSCC lines to 5FU: A) MINK1 knock out clones were generated using a lentiviral approach expressing two different sgRNAs (sgRNA#1 and #2) in Cas9

overexpressing 5FU sensitive lines and Immunoblotting (n=3) was performed with indicated antibodies in indicated cells. **B)** 5FU sensitive cells stably expressing MINK1sgRNA (#1 and #2) and NTsgRNA were treated with 5FU for 48h and cell viability was determined by MTT assay (n=3 and 2-way ANOVA, $*P < 0.05$).

Figure S9: MINK1 regulates the expression of p53 and thereby modulates 5FU sensitivity: A) Lysates were collected from indicated cells and immunoblotting (n=3) was performed with indicated antibodies. **B)** Left panel: MINK1 knock out clones were generated using a lentiviral approach expressing 2 different sgRNAs (#1 and #2) in Cas9 overexpressing SCC9 5FUR line. Immunoblotting (n=3) was performed with indicated antibodies in indicated cells. Right panel: Indicated 5FU resistant cells stably expressing MINK1sgRNA#1 and NTsgRNA were treated with 5FU for 48h and cell viability was determined by MTT assay (n=3 and 2-way ANOVA, $**P < 0.01$, $****P < 0.0001$). **C)** Indicated cells stably expressing MINK1shRNA#1 and NTshRNA were treated with 5FU for 48h and cell viability was determined by MTT assay (n=3 and 2-way ANOVA, $****P < 0.0001$).

Figure S10: MINK1 kinase activity is required for regulation of P53: 5FU sensitive cells were stably transfected with either pSilencer™ 4.1-CMV puro-MINK1WT or pSilencer™ 4.1-CMV puro-MINK1 K54R (kinase dead) followed by puromycin selection (up to 5µg/ml). Lysates were collected from indicated cells and immunoblotting (n=3) was performed with indicated antibodies.

Figure S11: Lestaurtinib negatively affects cancer stemness and tumor cell migration in chemoresistant OSCC: A) Left panel: Tumor spheroid assay was performed as described in methods with indicated cells with indicated concentration of 5FU and or lestaurtinib for 5 days.At the end of the experiment, spheroid photographs were captured using Leica DMIL microscope.Scale

bar, 200 μm. Right panel: Number of tumor spheres formed from the experiment in A was counted. (n = 3), 2-way ANOVA, $****P < 0.0001$. **B)** Indicated 5FU resistant OSCC lines and PDC2 cells were treated with 5FU and or Lestaurtinib for 48h, after which immunoblotting (n = 3) was performed with indicated antibodies. **C)** Indicated 5FU resistant OSCC lines and PDC cells were treated with vehicle control, 5FU (7.5 μM) and/or lestaurtinb (50 nM) for 48h, followed by subjecting to Boyden chamber assay. Left panel: representative photographs of Boyden chamber assay in each group. Right panel: Bar diagrams indicate the relative number of migrated cells (n=3 and $****P < 0.0001$ by 2-way ANOVA). Scale bars: 50 μm.

Table S1: Quantification score (raw data) of individual kinases in primary screening from high content analyser. This is in reference to figure 1B and 1C.

SL NO.	Kinases	Number of live cells in Kinase KO treated with vehicle control			Average	% cell viability	Number of live cells in Kinase KO treated with 5FU			Average	% cell viability	Survival fraction
1	**Negative Control sgRNA**	3176	3410	3218	3268	100	3565	3272	3387	3408	100	NA
2	**AATK**	1	0	5	2	0.06119951	12	31	26	23	0.674882629	11.02758216
3	**BCR**	493	494	495	494	15.11627907	692	507	1474	891	26.1443662	1.729550379
4	**CDK6**	91	94	94	93	2.845777234	398	416	1295	703	20.62793427	7.248611742
5	**DGKE**	45	46	47	46	1.407588739	39	22	38	33	0.968309859	0.687921004
6	**FAM20C**	1	10	10	7	0.214198286	6	9	0	5	0.146713615	0.684942991
7	**HYKK**	0	0	0	0	0	523	1032	1586	1047	30.72183099	0
8	**ADCK2**	4	5	6	5	0.152998776	15	33	27	25	0.733568075	4.794600939
9	**BRSK2**	348	349	350	349	10.67931457	570	882	921	791	23.2100939	2.173369251
10	**CDKN1A**	216	222	222	220	6.731946144	913	619	1486	1006	29.51877934	4.384880495
11	**DGKQ**	226	239	240	235	7.190942472	225	123	300	216	6.338028169	0.88139047
12	**FASTKD5**	0	12	12	8	0.244798042	465	557	544	522	15.31690141	62.56954225
13	**IKBKG**	0	0	0	0	0	29	10	21	20	0.58685446	0
14	**AK4**	7	6	14	9	0.275397797	8	2	11	7	0.205399061	0.745826813
15	**C19orf35**	21	24	24	23	0.70379437	319	413	516	416	12.20657277	17.34394774
16	**CKB**	18	24	30	24	0.734394125	484	170	978	544	15.96244131	21.73552426
17	**DLG2**	24	40	41	35	1.070991432	36	24	51	37	1.085680751	1.013715627
18	**FGFR4**	0	0	0	0	0	4	2	18	8	0.234741784	0
19	**ITPKC**	0	1	2	1	0.030599755	1	1	4	2	0.058685446	1.917840376
20	**AK8**	32	38	38	36	1.101591187	32	34	21	29	0.850938967	0.772463485
21	**CAMK1G**	33	46	47	42	1.285189718	923	723	1366	1004	29.4600939	22.92275877
22	**CLK1**	62	64	72	66	2.019583843	81	71	139	97	2.846244131	1.409322094
23	**EEF2K**	22	24	32	26	0.795593635	417	1079	928	808	23.70892019	29.80028891
24	**FGFRL1**	271	273	281	275	8.414932681	66	56	49	57	1.672535211	0.198758003
25	**KNDC1**	14	14	17	15	0.458996328	462	446	1819	909	26.67253521	58.11056338
26	**AKT1**	38	40	48	42	1.285189718	5	12	7	8	0.234741784	0.182651464
27	**CAMKK2**	408	408	411	409	12.51529988	925	607	991	841	24.67723005	1.971764983
28	**CMPK1**	312	314	322	316	9.669522644	808	1128	1967	1301	38.17488263	3.947959381
29	**EFEMP1**	209	209	212	210	6.425948592	830	1112	1211	1051	30.83920188	4.799167226
30	**GRIP2**	201	203	211	205	6.272949816	610	524	714	616	18.07511737	2.881438223
31	**KSR1**	678	680	688	682	20.86903305	904	1282	1195	1127	33.06924883	1.58460858
32	**ALDH18A1**	10	12	20	14	0.428396573	1025	1381	705	1037	30.42840376	71.0285882
33	**CASS4**	480	482	490	484	14.81028152	1654	1769	2052	1825	53.55046948	3.615763105
34	**COASY**	626	600	655	627	19.18604651	1567	1754	1203	1508	44.24882629	2.306302461
35	**EFNA3**	495	503	538	512	15.66707466	960	1396	1871	1409	41.34389671	2.638903407
36	**GSG2**	548	556	591	565	17.28886169	602	687	628	639	18.75	1.084513274
37	**LCK**	748	748	751	749	22.91921665	919	1181	1167	1089	31.95422535	1.394211061
38	**AMHR2**	7	8	12	9	0.275397797	22	32	54	36	1.056338028	3.835680751
39	**CCND3**	457	457	460	458	14.01468788	1251	1498	1583	1444	42.37089202	3.023320417
40	**CSNK1A1L**	557	560	572	563	17.22766218	1514	1402	1482	1466	43.01643192	2.496939601
41	**EPHA10**	419	423	439	427	13.06609547	1038	1011	1101	1050	30.80985915	2.358000462
42	**GTF2H1**	709	698	723	710	21.72582619	760	871	802	811	23.79694836	1.095329961
43	**LTBP4**	378	379	383	380	11.62790698	1264	1036	1147	1149	33.71478873	2.899471831
44	**AURKA**	315	287	346	316	9.669522644	42	38	55	45	1.320422535	0.13655509
45	**CCNE1**	641	646	669	652	19.95104039	1606	1563	2111	1760	51.64319249	2.588496212
46	**CSNK1E**	816	612	1044	824	25.21419829	1641	1489	1844	1658	48.65023474	1.929477757
47	**EPHA8**	860	863	878	867	26.52998776	1760	1426	1773	1653	48.50352113	1.828252676
48	**GTF2H3**	1661	1664	1676	1667	51.00979192	1693	1852	1612	1719	50.44014085	0.988832515
49	**MAP2K7**	1161	1023	1302	1162	35.55691554	1029	895	764	896	26.29107981	0.739408338
50	**AURKAIP1**	355	376	400	377	11.53610771	32	41	71	48	1.408450704	0.122090634
51	**CDK18**	425	431	455	437	13.37209302	1837	1726	1948	1837	53.90258216	4.030975709
52	**CSNK1G3**	751	671	594	672	20.5630355	1646	1602	1612	1620	47.53521127	2.311682596
53	**ERN2**	526	526	529	527	16.12607099	1730	1748	1724	1734	50.88028169	3.155156747
54	**HIPK2**	1396	1554	1715	1555	47.58261934	1932	1963	1835	1910	56.04460094	1.177837658
55	**MAPK1**	1235	1237	1245	1239	37.9130967	1337	1574	1394	1435	42.10680751	1.110613777
56	**AVP**	4	4	7	5	0.152998776	1245	1321	1148	1238	36.32629108	237.4286385
57	**CDK20**	1072	1075	1090	1079	33.01713586	1996	1810	2620	2142	62.85211268	1.903620984
58	**DCLK2**	858	862	878	866	26.499388	2158	1727	2682	2189	64.23122066	2.423875625
59	**ETNK2**	547	552	572	557	17.04406365	1707	1699	1838	1748	51.29107981	3.009322241
60	**HKDC1**	680	650	623	651	19.92044064	2148	1988	1957	2031	59.59507042	2.991654226
61	**MAPK11**	1930	1900	1966	1932	59.11872705	3955	3874	4159	3996	117.2535211	1.983356662
62	**MAPK14**	1427	1433	1457	1439	44.03304774	432	355	533	440	12.91079812	0.293207007
63	**PAK2**	1708	1815	1931	1818	55.63035496	777	689	784	750	22.00704225	0.395594137
64	**PKLR**	1634	1603	1668	1635	50.03059976	1367	1024	906	1099	32.24765258	0.644558585
65	**PTK2B**	2150	2052	2260	2154	65.91187271	1871	1071	1825	1589	46.62558685	0.70739284
66	**SRMS**	2052	2054	2062	2056	62.9130967	1912	2112	1280	1768	51.87793427	0.824596737
67	**TWF2**	1911	2091	2274	2092	64.01468788	1263	1201	1253	1239	36.3556338	0.56792644
68	**MINK1**	2789	2656	2526	2657	81.30354957	589	498	479	522	15.31690141	0.188391546
69	**PAPSS1**	1628	1198	2061	1629	49.84700122	812	759	958	843	24.73591549	0.496236782
70	**PLK1**	1240	1243	1258	1247	38.15789474	632	536	671	613	17.9870892	0.471385786
71	**RIPK1**	2367	2417	2476	2420	74.05140759	1365	1401	1029	1265	37.1185446	0.501253735
72	**STK11**	2469	2488	2453	2470	75.58139535	2073	1074	1884	1677	49.20774648	0.651056338
73	**TXK**	1507	1484	1551	1514	46.32802938	1417	1515	1031	1321	38.76173709	0.836680032
74	**MUSK**	1124	1122	1129	1125	34.4247246	861	575	358	598	17.54694836	0.509719353
75	**PDGFRB**	1342	1339	1348	1343	41.09547124	1048	855	356	753	22.09507042	0.537652198
76	**PLK3**	1534	1522	1552	1536	47.00122399	800	1175	617	864	25.35211268	0.539392606
77	**RIPK2**	1674	1658	1723	1685	51.56058752	1045	1310	864	1073	31.48474178	0.610635823
78	**STK25**	1498	1487	1539	1508	46.14443084	895	1243	991	1043	30.60446009	0.663231934
79	**UCK2**	1267	1263	1289	1273	38.95348837	899	1040	767	902	26.46713615	0.679454838
80	**MVK**	814	807	824	815	24.93880049	784	726	752	754	22.12441315	0.887148247
81	**PHKA2**	1112	1098	1144	1118	34.21052632	964	896	570	810	23.76760563	0.694745395
82	**PLXNB1**	1349	1342	1389	1360	41.61566707	859	1143	923	975	28.60915493	0.687461164
83	**RIPK3**	1684	1681	1690	1685	51.56058752	920	988	930	946	27.75821596	0.538361126
84	**TAF1**	1607	1605	1615	1609	49.23500612	1130	1318	1050	1166	34.21361502	0.69490425
85	**UCKL1**	1914	1902	1929	1915	58.59853121	1695	1606	1127	1476	43.30985915	0.73909462
86	**NADK**	974	959	1022	985	30.14075887	926	870	808	868	25.46948357	0.845017993
87	**PHKG1**	1388	1377	1426	1397	42.74785802	789	741	714	748	21.94835681	0.513437581
88	**PNCK**	1448	1444	1470	1454	44.49204406	963	954	876	931	27.31807512	0.613999102
89	**RIPK4**	1507	1676	1863	1682	51.46878825	1036	1399	1303	1246	36.56103286	0.71035348
90	**TESK1**	1708	1702	1726	1712	52.38678091	985	896	978	953	27.96361502	0.533791436
91	**UHMK1**	1986	1983	2001	1990	60.89351285	1025	1156	1356	1179	34.59507042	0.568124071
92	**NME3**	1166	1148	1187	1167	35.70991432	914	699	373	662	19.42488263	0.543963294
93	**PIK3C3**	1607	1589	1673	1623	49.66340269	982	851	723	852	25	0.503388786
94	**PRKAA1**	1548	1542	1578	1556	47.61321909	888	636	978	834	24.47183099	0.51397136
95	**RPS6KA4**	1992	1990	2000	1994	61.01591187	862	1475	606	981	28.78521127	0.471765649
96	**TGFA**	1969	1955	1986	1970	60.28151775	1036	1538	759	1111	32.59976526	0.540792045
97	**ULK3**	2314	2306	2361	2327	71.20563035	2292	2238	2241	2257	66.22652582	0.930074286
98	**NRP2**	874	983	1101	986	30.17135863	469	702	551	574	16.842723	0.558235485
99	**PIK3CG**	1432	1430	1437	1433	43.8494492	632	567	736	645	18.92605634	0.43161446
100	**PRKCA**	1440	1428	1455	1441	44.09424725	932	1023	835	930	27.28873239	0.618872848
101	**RPS6KA6**	1658	1651	1698	1669	51.07099143	758	865	564	729	21.39084507	0.418845307
102	**TNIK**	1352	1350	1357	1353	41.40146879	1175	897	922	998	29.28403756	0.707318808

103	**WNK3**	2428	2417	2442	2429	74.32680539	2406	2350	2393	2383	69.92370892	0.940760316
104	**NTPCR**	768	762	804	778	23.80660955	879	967	1097	981	28.78521127	1.209126869
105	**PIM2**	977	974	983	978	29.92656059	831	699	522	684	20.07042254	0.670655837
106	**PRKCH**	1797	1792	1808	1799	55.04895961	1524	1298	928	1250	36.67840376	0.666286956
107	**SGK2**	2349	2343	2367	2353	72.00122399	1026	1031	1324	1127	33.06924883	0.459287315
108	**TRAF3IP3**	1635	1627	1655	1639	50.15299878	845	874	798	839	24.6185446	0.490868845
109	**WNK4**	1474	1470	1496	1480	45.2876377	1377	1279	1166	1274	37.38262911	0.825448864
110	**NTRK1**	863	858	871	864	26.43818849	401	631	432	488	14.31924883	0.541612328
111	**PIM3**	1004	996	1024	1008	30.84455324	898	883	583	788	23.12206573	0.749632052
112	**PRKX**	1525	1511	1557	1531	46.84822521	2148	1924	2966	2346	68.83802817	1.469383906
113	**SPEG**	1968	1961	2008	1979	60.55691554	1970	1968	1948	1962	57.57042254	0.950682874
114	**TRIM27**	1666	1664	1671	1667	51.00979192	1482	1356	1053	1297	38.05751174	0.746082474
115	**ZAP70**	2017	2015	2022	2018	61.750306	1870	1865	1434	1723	50.55751174	0.818741072
116	**NUAK1**	893	884	905	894	27.35618115	700	522	299	507	14.87676056	0.543817153
117	**PIP5KL1**	1553	1538	1592	1561	47.76621787	695	744	862	767	22.50586854	0.471167062
118	**PSTK**	1802	1789	1848	1813	55.47735618	1703	1655	1424	1594	46.77230047	0.84308813
119	**SPHK2**	2291	2283	2320	2298	70.31823745	1336	1325	1380	1347	39.52464789	0.56208246
120	**TRPM6**	2103	2100	2118	2107	64.47368421	1232	1207	1350	1263	37.05985915	0.574805979
121	**ABL1**	1257	1252	1265	1258	38.49449204	1969	1511	2226	1902	55.80985915	1.449814147
122	**ACVR2B**	750	746	766	754	23.07221542	290	517	306	371	10.88615023	0.471829429
123	**AKAP8**	1750	1748	1758	1752	53.61077111	785	880	1029	898	26.34976526	0.491501329
124	**CAD**	1395	1388	1405	1396	42.71725826	522	537	519	526	15.4342723	0.361312334
125	**CDC42BPB**	2854	2849	2877	2860	87.51529988	1491	1521	1266	1426	41.842723	0.478118947
126	**CDK7**	1302	1289	1321	1304	39.90208078	544	923	168	545	15.99178404	0.400775692
127	**COL4A3BP**	1469	1462	1512	1481	45.31823745	154	278	333	255	7.482394366	0.165107797
128	**ACVRL1**	832	814	853	833	25.48959608	638	256	435	443	12.99882629	0.509965958
129	**ALK**	1473	1455	1542	1490	45.59363525	1139	687	541	789	23.15140845	0.5077772
130	**CALM3**	1650	1639	1688	1659	50.76499388	987	1035	843	955	28.02230047	0.55200047
131	**CDC42SE2**	2416	2405	2445	2422	74.1126071	748	599	918	755	22.15375587	0.298920207
132	**CDK9**	1481	1473	1513	1489	45.5630355	748	745	769	754	22.12441315	0.485578121
133	**CSF1R**	2524	2514	2546	2528	77.35618115	1278	1100	1276	1218	35.73943662	0.462011388
134	**ADCK4**	1596	1590	1626	1604	49.08200734	625	663	512	600	17.6056338	0.358698325
135	**ALPK1**	1477	1472	1488	1479	45.25703794	878	847	786	837	24.55985915	0.542674914
136	**CAMK2D**	1774	1755	1805	1778	54.40636475	874	758	1032	888	26.05633803	0.478920769
137	**CDK13**	1426	1415	1488	1443	44.15544676	732	700	845	759	22.27112676	0.504380057
138	**CDKL3**	1494	1487	1507	1496	45.77723378	805	793	961	853	25.02934272	0.546763984
139	**CSK**	3047	3031	3081	3053	93.42105263	1000	1146	881	1009	29.60680751	0.31691794
140	**ADCK5**	1363	1355	1410	1376	42.10526316	670	621	815	702	20.59859155	0.489216549
141	**ALPK3**	1701	1692	1713	1702	52.08078335	875	880	606	787	23.092723	0.44340199
142	**CAMK4**	2392	2386	2422	2400	73.43941248	1103	1392	943	1146	33.62676056	0.45788439
143	**CDK14**	2497	2495	2505	2499	76.46878825	1248	1494	1011	1251	36.70774648	0.480035676
144	**CHEK1**	1069	1061	1080	1070	32.74173807	338	276	421	345	10.12323944	0.309184547
145	**DAPK3**	2702	2697	2728	2709	82.89473684	895	874	1051	940	27.58215962	0.332737164
146	**ADK**	1725	1723	1730	1726	52.81517748	801	800	244	615	18.04577465	0.341677819
147	**ATR**	1618	1604	1635	1619	49.54100367	412	459	503	458	13.43896714	0.271269578
148	**CARD11**	1803	1794	1851	1816	55.56915545	998	963	793	918	26.93661972	0.484740491
149	**CDK15**	2388	2386	2396	2390	73.13341493	1036	964	1078	1026	30.1056338	0.411653604
150	**CHKA**	1607	1613	1631	1617	49.47980416	925	933	1058	972	28.52112676	0.576419556
151	**DCK**	1685	1673	1703	1687	51.62178703	1131	1108	1700	1313	38.52699531	0.746332073
152	**AGK**	2896	2886	2939	2907	88.95348837	789	635	910	778	22.8286385	0.256635675
153	**AURKB**	2389	2383	2407	2393	73.2252142	652	616	733	667	19.57159624	0.267279467
154	**CCL5**	1688	1677	1711	1692	51.7747858	894	859	752	835	24.50117371	0.473225979
155	**CDK16**	2647	2641	2680	2656	81.27294982	923	901	1008	944	27.69953052	0.340821031
156	**CKMT1A**	1477	1472	1485	1478	45.22643819	737	725	743	735	21.56690141	0.476864911
157	**DCLK3**	2345	2336	2366	2349	71.87882497	847	828	926	867	25.44014085	0.353930951
158	**AK1**	1778	1767	1810	1785	54.62056304	666	527	649	614	18.01643192	0.329847056
159	**AURKC**	2390	2385	2416	2397	73.34761322	1537	1542	1169	1416	41.54929577	0.566471
160	**CCNB1**	2326	2323	2332	2327	71.20563035	1201	1415	1080	1232	36.15023474	0.507687869
161	**CDK19**	2427	2424	2436	2429	74.32680539	1157	1231	867	1085	31.83685446	0.428336107
162	**CKMT2**	2230	2219	2247	2232	68.29865361	489	443	844	592	17.37089202	0.254337254
163	**DGKZ**	2338	2332	2374	2348	71.84822521	667	521	750	646	18.95539906	0.263825571
164	**AKAP13**	949	942	959	950	29.06976744	752	455	365	524	15.37558685	0.528920188
165	**BCKDK**	2333	2322	2362	2339	71.57282742	1186	1033	1321	1180	34.62441315	0.483764781
166	**CCND1**	2591	2576	2630	2599	79.52876377	1014	966	954	978	28.6971831	0.360840302
167	**CDK3**	2353	2345	2394	2364	72.3378213	647	625	972	748	21.94835681	0.303414679
168	**CLP1**	1421	1419	1429	1423	43.54345165	454	510	341	435	12.76408451	0.293134421
169	**DGUOK**	2426	2419	2436	2427	74.26560588	698	648	604	650	19.07276995	0.256818344
170	**AKAP14**	976	956	1014	982	30.04895961	457	451	346	418	12.26525822	0.408175803
171	**BMPR2**	1320	1305	1386	1337	40.91187271	712	782	1011	835	24.50117371	0.598876856
172	**CCNK**	1646	1641	1669	1652	50.55079559	953	841	735	843	24.73591549	0.489327917
173	**CDK4**	1677	1535	1423	1545	47.27662179	502	983	105	530	15.55164319	0.328949967
174	**CMPK2**	2811	2805	2838	2818	86.23011016	800	783	1105	896	26.29107981	0.304894425
175	**DLG3**	1462	1455	1475	1464	44.79804162	1545	1488	1524	1519	44.57159624	0.994945195
176	**AKAP7**	1032	1027	1055	1038	31.7625459	746	889	675	770	22.59389671	0.711337712
177	**BMX**	1667	1665	1675	1669	51.07099143	1124	1036	1017	1059	31.07394366	0.608446063
178	**CDC42BPA**	2806	2799	2816	2807	85.89351285	1554	2231	1264	1683	49.38380282	0.574942172
179	**CDK5R1**	1458	1454	1480	1464	44.79804162	847	1165	787	933	27.37676056	0.61111512
180	**CNTLN**	2451	2449	2456	2452	75.03059976	453	631	785	623	18.28051643	0.243640814
181	**DOK1**	1350	1345	1358	1351	41.34026928	792	899	958	883	25.90962441	0.626740582
182	**DSTYK**	32	26	50	36	1.101591187	73	69	89	77	2.259389671	2.051023735
183	**EPHB2**	62	59	77	66	2.019583843	1	0	2	1	0.029342723	0.014529094
184	**FGFR2**	66	53	82	67	2.050183599	8	9	10	9	0.264084507	0.128810174
185	**GSK3B**	73	60	122	85	2.600979192	2	8	14	8	0.234741784	0.090251312
186	**IKBKE**	18	13	44	25	0.76499388	19	12	8	13	0.381455399	0.498638498
187	**KSR2**	8	6	13	9	0.275397797	10	5	21	12	0.352112676	1.27856025
188	**DYRK4**	293	291	298	294	8.996328029	221	191	71	161	4.724178404	0.52512296
189	**EPHB4**	607	604	613	608	18.60465116	102	147	129	126	3.697183099	0.198723592
190	**FGFR3**	68	66	76	70	2.141982864	150	320	223	231	6.778169014	3.16443662
191	**GUK1**	137	129	148	138	4.222766218	7	12	2	7	0.205399061	0.048640879
192	**INSRR**	43	29	78	50	1.52998776	14	11	14	13	0.381455399	0.249319249
193	**LRGUK**	4	3	2	3	0.091799266	5	2	14	7	0.205399061	2.237480438
194	**EIF2AK3**	73	66	83	74	2.264381885	247	511	502	420	12.32394366	5.442519985
195	**EPHB6**	351	347	373	357	10.92411261	362	336	406	368	10.79812207	0.988466748
196	**FGR**	844	837	854	845	25.85679315	10	23	15	16	0.469483568	0.018157069
197	**HCK**	560	555	583	566	17.31946144	178	141	170	163	4.78286385	0.27615546
198	**IP6K1**	969	957	987	971	29.7123623	411	412	482	435	12.76408451	0.429588344
199	**LRRK1**	4	17	24	15	0.458996328	485	423	403	437	12.82276995	27.93654147
200	**ENG**	778	769	790	779	23.8372093	440	412	387	413	12.1185446	0.508387725
201	**ERBB3**	87	81	117	95	2.906976744	164	256	282	234	6.866197183	2.361971831
202	**FLT3**	1174	1160	1194	1176	35.98531212	18	26	16	20	0.58685446	0.016308166
203	**HGS**	749	741	796	762	23.31701346	374	584	203	387	11.3556338	0.487010647
204	**IP6K3**	1288	1281	1298	1289	39.44308446	545	632	665	614	18.01643192	0.456770361
205	**LTK**	1536	1532	1558	1542	47.18482252	95	97	96	96	2.816901408	0.059699311
206	**EPGN**	963	961	968	964	29.49816401	471	400	413	428	12.55868545	0.425744648
207	**ERCC3**	1193	1186	1203	1194	36.53610771	415	389	459	421	12.35328638	0.338111725

208	**FLT4**	1556	1552	1578	1562	47.79681763	845	901	870	872	25.58685446	0.535325483
209	**HIPK4**	1119	1116	1125	1120	34.27172583	998	920	710	876	25.70422535	0.750012575
210	**JAK1**	1032	1027	1043	1034	31.64014688	457	473	549	493	14.46596244	0.457202759
211	**MAGI1**	972	959	997	976	29.86536108	199	233	321	251	7.365023474	0.246607548
212	**EPHA1**	827	820	867	838	25.64259486	438	415	407	420	12.32394366	0.48060439
213	**ERN1**	1347	1337	1360	1348	41.24847001	714	901	482	699	20.51056338	0.497244222
214	**FN3K**	3503	3494	3539	3512	107.4663403	623	597	832	684	20.07042254	0.186760082
215	**HK1**	2447	2441	2465	2451	75	1203	1196	1210	1203	35.29929577	0.470657277
216	**JAK2**	1933	1930	1948	1937	59.27172583	909	978	996	961	28.19835681	0.475747187
217	**MAP2K2**	3192	3187	3200	3193	97.70501836	1142	1185	1207	1178	34.5657277	0.35377638
218	**EPHA2**	1541	1538	1556	1545	47.27662179	537	531	492	520	15.25821596	0.322743364
219	**FAM20B**	1512	1504	1523	1513	46.29742962	874	898	793	855	25.08802817	0.541888143
220	**FN3KRP**	2710	2698	2743	2717	83.13953488	1421	1141	978	1180	34.62441315	0.416461473
221	**HK3**	2399	2394	2428	2407	73.65361077	2872	2610	3038	2840	83.33333333	1.131422241
222	**JAK3**	1781	1763	1802	1782	54.52876377	823	799	844	822	24.11971831	0.442330188
223	**MAP3K11**	2278	2263	2344	2295	70.22643819	985	910	1081	992	29.10798122	0.414487506
224	**EPHA5**	1692	1687	1715	1698	51.95838433	455	469	483	469	13.76173709	0.264860759
225	**FASTK**	912	904	926	914	27.96817625	724	809	672	735	21.56690141	0.771122908
226	**GRK6**	1790	1784	1817	1797	54.9877601	1001	892	846	913	26.7899061	0.487197625
227	**HUNK**	1579	1576	1588	1581	48.37821297	1156	1345	988	1163	34.12558685	0.705391637
228	**KALRN**	1504	1502	1512	1506	46.08323133	1287	1045	872	1068	31.33802817	0.68003105
229	**MAP3K12**	3173	3160	3189	3174	97.12362301	1041	991	704	912	26.76056338	0.275530942
230	**EPHA7**	1522	1514	1566	1534	46.94002448	412	478	529	473	13.87910798	0.295677476
231	**FASTKD3**	1796	1789	1809	1798	55.01835985	685	714	935	778	22.8286385	0.414927645
232	**GRK7**	2391	2387	2413	2397	73.34761322	930	656	811	799	23.44483568	0.319640063
233	**IGF2R**	1646	1639	1656	1647	50.39779682	812	668	860	780	22.88732394	0.454133422
234	**KCNH5**	1210	1199	1239	1216	37.20930233	702	952	1319	991	29.0786385	0.78148841
235	**MAP3K15**	3002	2991	3037	3010	92.10526316	1023	1151	1240	1138	33.39201878	0.362541918
236	**EPHB1**	2352	2338	2387	2359	72.18482252	566	970	492	676	19.83568075	0.274790185
237	**FES**	1056	1047	1095	1066	32.61933905	407	547	699	551	16.16784038	0.495651992
238	**GSK3A**	2365	2359	2383	2369	72.49082007	899	1178	1190	1089	31.95422535	0.44080375
239	**IKBKB**	3053	3047	3071	3057	93.54345165	955	1003	892	950	27.87558685	0.297996133
240	**KDR**	1263	1249	1289	1267	38.76988984	642	837	981	820	24.06103286	0.620611329
241	**MAP3K2**	3827	3819	3871	3839	117.4724602	1146	1202	1399	1249	36.64906103	0.311980025
242	**MAP3K5**	43	25	67	45	1.376988984	2	9	4	5	0.146713615	0.106546688
243	**MAPKAPK3**	104	87	172	121	3.702570379	7	0	35	14	0.410798122	0.110949443
244	**MRC2**	14	4	48	22	0.673194614	1	5	12	6	0.176056338	0.261523688
245	**NME6**	25	16	52	31	0.948592411	7	2	0	3	0.088028169	0.092798728
246	**PAK1**	15	10	38	21	0.642594859	6	5	1	4	0.117370892	0.182651464
247	**PI4KA**	9	2	22	11	0.336597307	7	2	3	4	0.117370892	0.34869825
248	**MAP3K7CL**	299	283	333	305	9.332925337	7	8	12	9	0.264084507	0.028296006
249	**MAST1**	297	282	351	310	9.485924113	296	257	296	283	8.30399061	0.875401333
250	**MST1R**	2627	2622	2656	2635	80.63035496	12	10	23	15	0.440140845	0.005458749
251	**NOL9**	2850	2847	2856	2851	87.23990208	28	34	28	30	0.88028169	0.010090356
252	**PAK4**	1749	1747	1757	1751	53.58017136	2	1	3	2	0.058685446	0.001095283
253	**PI4KB**	263	261	268	264	8.078335373	1	1	10	4	0.117370892	0.014529094
254	**MAP3K9**	1541	1538	1547	1542	47.18482252	685	700	802	729	21.39084507	0.453341645
255	**MAST2**	2442	2435	2455	2444	74.78580171	54	25	20	33	0.968309859	0.012947777
256	**MTOR**	637	632	660	643	19.67564259	530	345	376	417	12.23591549	0.621881366
257	**NPR1**	2393	2379	2413	2395	73.28641371	435	411	579	475	13.93779343	0.190182501
258	**PAK6**	1664	1656	1711	1677	51.31578947	215	255	274	248	7.276995305	0.141808114
259	**PICK1**	662	650	677	663	20.2876377	367	210	236	271	7.951877934	0.391956819
260	**MAP4K2**	411	404	451	422	12.9130967	859	810	986	885	25.96830986	2.011005607
261	**MAST3**	1719	1717	1724	1720	52.63157895	1007	1012	990	1003	29.43075117	0.559184272
262	**MYLK3**	2657	2655	2662	2658	81.33414933	765	760	1094	873	25.61619718	0.314950084
263	**NPR2**	2938	2936	2943	2939	89.93268054	1201	1203	1985	1463	42.92840376	0.477339311
264	**PAK7**	2829	2826	2835	2830	86.59730722	1078	1103	873	1018	29.87089202	0.344940195
265	**PIK3R2**	2140	2128	2158	2142	65.54467564	578	600	658	612	17.95774648	0.273977197
266	**MAP4K3**	2393	2383	2436	2404	73.56181151	989	678	1255	974	28.57981221	0.388514252
267	**MERTK**	2929	2926	2944	2933	89.74908201	943	905	1011	953	27.96361502	0.311575499
268	**NADK2**	3602	3592	3615	3603	110.250918	1473	1429	1130	1344	39.43661972	0.357698788
269	**NRBP2**	3296	3287	3332	3305	101.1321909	744	727	878	783	22.97535211	0.227181394
270	**PAPSS2**	2080	2074	2098	2084	63.76988984	797	374	962	711	20.86267606	0.327155592
271	**PIK3R6**	2124	2118	2142	2128	65.11627907	659	730	984	791	23.2100939	0.356440728
272	**MAPK10**	2442	2439	2457	2446	74.84700122	346	338	477	387	11.3556338	0.151717953
273	**MFHAS1**	3392	3384	3403	3393	103.8249694	1309	1259	1110	1226	35.9741784	0.346488697
274	**NEK6**	3467	3459	3496	3474	106.3035496	2249	2193	2230	2224	65.25821596	0.613885578
275	**NTRK2**	3489	3485	3505	3493	106.8849449	2274	2298	2070	2214	64.96478873	0.607801115
276	**PDK4**	3393	3388	3404	3395	103.8861689	2368	2306	2352	2342	68.72065728	0.661499582
277	**PINK1**	2489	2476	2514	2493	76.28518972	2034	2066	1825	1975	57.95187793	0.759674036
278	**MAPK12**	3419	3396	3475	3430	104.9571603	1023	1110	1212	1115	32.71713615	0.311718953
279	**MORN1**	3278	3268	3339	3295	100.8261934	1354	1418	1470	1414	41.49061033	0.411506266
280	**NEK7**	3481	3473	3492	3482	106.5483476	1579	1482	1868	1643	48.2100939	0.45247153
281	**NTRK3**	3440	3435	3466	3447	105.4773562	2237	2489	2276	2334	68.48591549	0.649294957
282	**PDPK1**	3667	3656	3681	3668	112.2399021	2304	2214	2505	2341	68.69131455	0.612004406
283	**PIP4K2C**	3623	3617	3659	3633	111.1689106	1722	1737	1974	1811	53.13967136	0.478008384
284	**MAPK15**	3588	3579	3600	3589	109.8225214	699	756	666	707	20.74530516	0.18889846
285	**MPP2**	3695	3689	3725	3703	113.3108935	1249	1459	1135	1281	37.58802817	0.331724753
286	**NEK9**	3433	3431	3441	3435	105.1101591	3477	3467	3616	3520	103.286385	0.982648926
287	**OBSCN**	3335	3333	3340	3336	102.0807834	2093	2077	1983	2051	60.18192488	0.58955195
288	**PFKFB3**	3388	3385	3394	3389	103.7025704	3195	3156	2652	3001	88.05751174	0.849135286
289	**PIP5K1C**	4016	4002	4036	4018	122.9498164	1341	1436	1495	1424	41.78403756	0.339846279
290	**MAPK3**	3579	3571	3626	3592	109.9143207	591	674	766	677	19.86502347	0.180731895
291	**MPP3**	4172	4165	4182	4173	127.6927785	1156	1362	1040	1186	34.80046948	0.272532792
292	**NLK**	3872	3862	3900	3878	118.6658507	2254	2236	2626	2372	69.60093897	0.5865288
293	**PACSIN1**	4008	3994	4043	4015	122.8580171	1242	1256	1309	1269	37.23591549	0.303080876
294	**PFKP**	3702	3686	3748	3712	113.5862913	2456	2974	1596	2342	68.72065728	0.605008373
295	**PKM**	3299	3285	3346	3310	101.2851897	1456	1448	1857	1587	46.56690141	0.459760223
296	**MAPK7**	2729	2719	2763	2737	83.75152999	1356	2058	858	1424	41.78403756	0.498904767
297	**MPP4**	4015	4011	4037	4021	123.0416157	2845	3336	2078	2753	80.78051643	0.656530037
298	**NME2**	3647	3645	3652	3648	111.627907	1850	2676	754	1760	51.64319249	0.462636933
299	**PACSIN3**	3795	3783	3810	3796	116.1566707	850	1252	364	822	24.11971831	0.207648154
300	**PI4K2A**	3041	3033	3082	3052	93.39045288	2200	2527	1657	2128	62.44131455	0.668604902
301	**PKN1**	3542	3535	3555	3544	108.4455324	1302	1319	1075	1232	36.15023474	0.33334923
302	**PLK2**	1100	1096	1122	1106	33.84332925	957	882	588	809	23.73826291	0.701416304
303	**PRKCE**	1589	1577	1604	1590	48.65361077	1899	1689	1812	1800	52.81690141	1.085570024
304	**RBKS**	1248	1241	1288	1259	38.5250918	1312	1300	1483	1365	40.0528169	1.039655327
305	**RPS6KB2**	1738	1736	1743	1739	53.2129743	1045	1357	631	1011	29.66549296	0.55748609
306	**SPEF2**	1952	1945	1962	1953	59.76132191	1110	1492	578	1060	31.10328638	0.520458474
307	**TAOK2**	1866	1861	1889	1872	57.28274174	780	904	500	728	21.36150235	0.372913406
308	**PLXNA1**	852	849	861	854	26.13219094	850	769	922	847	24.85328638	0.951060186
309	**PRKD1**	1405	1392	1424	1407	43.05385557	1472	1448	1691	1537	45.09976526	1.047519779
310	**RELN**	1854	1844	1900	1866	57.09914321	1741	1711	1396	1616	47.41784038	0.830447494
311	**RPS6KL1**	2362	2354	2382	2366	72.39902081	1599	1698	1266	1521	44.63028169	0.616448692
312	**SPHK1**	1875	1871	1897	1881	57.55813953	1402	1557	959	1306	38.32159624	0.665789349

313	**TAOK3**	1874	1866	1885	1875	57.374541	910	1319	303	844	24.76525822	0.431641941
314	**PLXNC1**	1264	1243	1306	1271	38.89228886	980	1031	854	955	28.02230047	0.720510448
315	**PRKD2**	1037	1020	1105	1054	32.25214198	1074	1048	1163	1095	32.13028169	0.996221637
316	**RET**	2282	2276	2312	2290	70.07343941	998	1376	464	946	27.75821596	0.396130348
317	**SBK1**	2358	2355	2367	2360	72.21542228	1185	1256	919	1120	32.86384977	0.455080767
318	**SQSTM1**	2933	2919	2953	2935	89.81028152	1213	1451	747	1137	33.36267606	0.371479473
319	**TEC**	2414	2406	2461	2427	74.26560588	1371	1783	794	1316	38.61502347	0.519958371
320	**PLXND1**	991	986	999	992	30.35495716	1035	1003	1199	1079	31.66079812	1.043019035
321	**PRKG1**	2293	2287	2311	2297	70.2876377	1295	1427	1040	1254	36.79577465	0.523502793
322	**RIOK2**	1906	1903	1921	1910	58.44553244	1010	1207	1059	1092	32.04225352	0.54824128
323	**SBK2**	2328	2326	2333	2329	71.26682987	980	1095	739	938	27.52347418	0.38620315
324	**SRPK3**	1766	1764	1771	1767	54.06976744	1397	1343	344	1028	30.16431925	0.557877732
325	**TEK**	2821	2819	2826	2822	86.35250918	1150	1949	138	1079	31.66079812	0.366645954
326	**PPIP5K1**	811	809	816	812	24.84700122	928	935	1269	1044	30.63380282	1.232897384
327	**PRKG2**	1893	1890	1899	1894	57.95593635	1543	1499	537	1193	35.00586854	0.604008334
328	**ROCK2**	3036	3033	3045	3038	92.9620563	1206	1405	911	1174	34.44835681	0.370563628
329	**SGK223**	3241	3239	3249	3243	99.23500612	1341	1434	942	1239	36.3556338	0.366358962
330	**STK10**	2616	2603	2632	2617	80.07955936	1482	1598	1561	1547	45.39319249	0.566851177
331	**TESK2**	2383	2371	2431	2395	73.28641371	1456	1495	1234	1395	40.93309859	0.558535976
332	**PPP4C**	823	814	850	829	25.36719706	848	829	924	867	25.44014085	1.002875516
333	**PRPS1L1**	1665	1654	1694	1671	51.13219094	1498	1442	1035	1325	38.87910798	0.760364601
334	**ROR2**	2645	2637	2677	2653	81.18115055	1975	1869	47	1297	38.05751174	0.468797393
335	**SGMS1**	3360	3351	3381	3364	102.9375765	1463	1660	1563	1562	45.83333333	0.445253666
336	**STK16**	2479	2464	2515	2486	76.07099143	810	998	397	735	21.56690141	0.283510192
337	**TEX14**	2652	2645	2692	2663	81.4871481	1487	1686	1360	1511	44.33685446	0.544096284
338	**PRKACA**	352	350	357	353	10.80171359	855	940	1079	958	28.11032864	2.602395297
339	**PRPSAP2**	1612	1604	1623	1613	49.35740514	1490	1372	1239	1367	40.11150235	0.812674455
340	**RP2**	1488	1475	1522	1495	45.74663403	1311	1187	886	1128	33.09859155	0.723519714
341	**SH3BP4**	2477	2470	2511	2486	76.07099143	1184	1254	808	1082	31.74882629	0.417357861
342	**STK32C**	1816	1814	1824	1818	55.63035496	1954	1957	2362	2091	61.3556338	1.102916454
343	**TIE1**	1764	1762	1769	1765	54.00856793	1638	1617	1278	1511	44.33685446	0.820922608
344	**PRKACG**	670	663	680	671	20.53243574	1544	1754	1556	1618	47.47652582	2.312269544
345	**PTCD2**	1431	1427	1453	1437	43.97184823	1272	1247	805	1108	32.51173709	0.739376178
346	**RPS6KA1**	1859	1857	1864	1860	56.91554468	768	837	426	677	19.86502347	0.349026326
347	**SHPK**	2292	2280	2307	2293	70.16523868	2370	2333	2641	2448	71.83098592	1.023740349
348	**STK35**	2376	2360	2425	2387	73.04161567	2372	2329	2370	2357	69.16079812	0.946868405
349	**TJP2**	2517	2511	2553	2527	77.3255814	2811	2784	3690	3095	90.8157277	1.174459035
350	**PRKAG1**	724	721	730	725	22.18482252	883	850	1390	1041	30.54577465	1.376877125
351	**PTK6**	2325	2318	2338	2327	71.20563035	1654	1918	1774	1782	52.28873239	0.734334239
352	**RPS6KA2**	1953	1946	1978	1959	59.94492044	1207	1324	634	1055	30.95657277	0.516416946
353	**SIK1**	1439	1424	1466	1443	44.15544676	2016	2267	2287	2190	64.26056338	1.455325857
354	**STRADB**	1701	1692	1749	1714	52.44798042	2265	2350	2777	2464	72.30046948	1.378517703
355	**TK1**	2311	2309	2319	2313	70.77723378	1460	1650	1684	1598	46.88967136	0.662496524
356	**PRKAG3**	571	569	576	572	17.50305998	867	822	1800	1163	34.12558685	1.94969262
357	**PTK7**	845	832	861	846	25.8873929	833	744	886	821	24.09037559	0.930583303
358	**RPS6KA5**	993	980	1042	1005	30.75275398	1170	1075	1760	1335	39.17253521	1.273789503
359	**SKAP1**	1059	1054	1085	1066	32.61933905	1196	1191	1591	1326	38.9084507	1.19280316
360	**TAF1L**	2499	2497	2504	2500	76.499388	2230	2219	1434	1961	57.54107981	0.752176995
361	**TP53RK**	1365	1363	1370	1366	41.79926561	1099	1097	303	833	24.44248826	0.584758797
362	**TPD52L3**	1	-2	7	2	0.06119951	814	963	1301	1026	30.1056338	491.9260563
363	**TTBK2**	260	258	268	262	8.017135863	711	654	2118	1161	34.06690141	4.249260832
364	**ZC3HC1**	340	338	345	341	10.43451652	503	402	1090	665	19.5129108	1.87003497
365	**AK7**	487	479	498	488	14.93268054	884	967	1389	1080	31.69014085	2.122200416
366	**BMP2K**	977	963	1012	984	30.11015912	992	885	1126	1001	29.37206573	0.975486898
367	**CAMKK1**	844	833	885	854	26.13219094	818	705	826	783	22.97535211	0.879197315
368	**TPR**	81	77	103	87	2.662178703	669	600	1389	886	25.99765258	9.765555016
369	**TTC33**	328	325	334	329	10.06731946	738	890	1816	1148	33.68544601	3.346019379
370	**AAK1**	389	380	404	391	11.96450428	539	531	991	687	20.1584507	1.684854652
371	**AK9**	709	703	739	717	21.94002448	835	704	1323	954	27.99295775	1.275885438
372	**BMPR1B**	765	758	778	767	23.47001224	747	733	701	727	21.33215962	0.908911312
373	**CAMKV**	1002	992	1030	1008	30.84455324	985	848	1053	962	28.22769953	0.915159941
374	**TRIB3**	24	7	62	31	0.948592411	664	783	1106	851	24.97065728	26.3239058
375	**TWF1**	296	288	343	309	9.455324357	1098	938	1606	1214	35.62206573	3.767408116
376	**ACVR1**	288	276	303	289	8.843329253	789	669	1212	890	26.11502347	2.953076011
377	**AKAP8L**	359	352	399	370	11.32190942	677	513	1162	784	23.00469484	2.031874128
378	**BRSK1**	920	911	932	921	28.18237454	991	921	1271	1061	31.13262911	1.104684386
379	**CASK**	877	864	914	885	27.08078335	662	578	80	440	12.91079812	0.47675128
380	**TRIM24**	48	41	82	57	1.744186047	1085	910	1335	1110	32.57042254	18.67370892
381	**TYK2**	272	270	280	274	8.384332925	1758	1564	1580	1634	47.94600939	5.71852404
382	**ACVR1B**	372	364	383	373	11.41370869	729	653	1276	886	25.99765258	2.2777568
383	**AKT2**	318	312	345	325	9.944920441	670	888	890	816	23.94366197	2.407627302
384	**BTK**	907	900	920	909	27.81517748	860	764	809	811	23.79694836	0.855538253
385	**CCL2**	897	893	919	903	27.63157895	1010	978	1363	1117	32.7758216	1.186172591
386	**TRPM7**	137	125	152	138	4.222766218	1750	1577	1512	1613	47.32981221	11.20824828
387	**UCK1**	272	262	315	283	8.659730722	881	891	1078	950	27.87558685	3.21899003
388	**ACVR1C**	511	500	534	515	15.75887393	1046	938	1874	1286	37.73474178	2.394507498
389	**ALPK2**	655	646	691	664	20.31823745	920	806	1802	1176	34.50704225	1.698328525
390	**BUB1**	595	589	613	599	18.32925337	718	689	1107	838	24.58920188	1.341527742
391	**CCL8**	903	900	918	907	27.75397797	928	840	1079	949	27.84624413	1.00332443
392	**TSKS**	254	249	262	255	7.802937576	1073	953	1568	1198	35.15258216	4.505044647
393	**VRK2**	462	456	480	466	14.25948592	1509	1471	2279	1753	51.43779343	3.607268432
394	**ACVR2A**	342	333	363	346	10.5875153	898	854	1398	1050	30.80985915	2.910017911
395	**ANKK1**	757	752	783	764	23.37821297	1379	1285	1818	1494	43.83802817	1.875165917
396	**BUB1B**	1075	1070	1083	1076	32.9253366	1325	1199	1298	1274	37.38262911	1.135375761
397	**CCNH**	1037	1018	1068	1041	31.85434517	1066	1016	1194	1092	32.04225352	1.005898987
398	**TSSK1B**	281	266	347	298	9.11872705	708	608	1138	818	24.00234742	2.632203737
399	**WEE2**	652	647	675	658	20.13463892	984	928	1421	1111	32.59976526	1.619088645
400	**ADCK1**	415	410	426	417	12.76009792	679	547	1600	942	27.64084507	2.166193806
401	**ARAF**	288	285	303	292	8.935128519	813	975	1374	1054	30.92723005	3.461307801
402	**CAMK1**	1631	1618	1647	1632	49.93880049	1328	1271	473	1024	30.04694836	0.601675412
403	**CDC7**	706	698	750	718	21.97062424	649	646	448	581	17.04812207	0.775950737
404	**TSSK2**	286	279	299	288	8.812729498	511	426	1268	735	21.56690141	2.447244229
405	**WNK1**	375	371	397	381	11.65850673	741	678	996	805	23.62089202	2.026064964
406	**ADCK3**	606	598	617	607	18.57405141	1208	1098	1003	1103	32.36502347	1.742485943
407	**ATM**	778	764	813	785	24.02080783	1014	945	1773	1244	36.50234742	1.519613648
408	**CAMK2B**	1044	1038	1080	1054	32.25214198	1023	932	1021	992	29.10798122	0.902513118
409	**CDK10**	880	873	890	881	26.95838433	872	797	920	863	25.32276995	0.939328175
410	**TSSK3**	203	192	232	209	6.395348837	527	446	962	645	18.92605634	2.959346991
411	**YES1**	432	422	466	440	13.46389229	1269	1172	1456	1299	38.11619718	2.830993918
412	**ADPGK**	604	600	626	610	18.66585067	1018	915	1448	1127	33.06924883	1.771644347
413	**ATMIN**	500	498	505	501	15.33047736	941	906	1399	1082	31.74882629	2.070961364
414	**CAMK2G**	1023	1021	1028	1024	31.33414933	979	892	934	935	27.43544601	0.875576539
415	**CDK11B**	1045	1042	1051	1046	32.00734394	909	868	539	772	22.65258216	0.70773077
416	**TSSK4**	110	107	119	112	3.427172583	633	802	830	755	22.15375587	6.46414948
417	**ZAK**	362	350	380	364	11.13831089	1164	1055	1576	1265	37.1185446	3.332511092

418	**AK5**	386	378	427	397	12.14810282	739	697	1210	882	25.88028169	2.130396992
419	**BLK**	384	377	397	386	11.81150551	956	857	1868	1227	36.00352113	3.048173758
420	**CAMK2N1**	799	795	821	805	24.63280294	821	716	977	838	24.58920188	0.998229959
421	**CDK17**	287	277	300	288	8.812729498	377	262	762	467	13.70305164	1.554915721
422	**CDK2**	197	188	233	206	6.303549572	455	334	723	504	14.78873239	2.346095993
423	**CLK4**	1874	1871	1889	1878	57.46634027	1862	1751	1925	1846	54.16666667	0.94258076
424	**DCAKD**	1901	1896	1909	1902	58.20073439	1807	1792	1537	1712	50.23474178	0.863129002
425	**DYRK1A**	854	848	872	858	26.25458996	960	854	1375	1063	31.19131455	1.18803282
426	**FASTKD2**	2979	2973	2997	2983	91.27906977	2271	2144	1762	2059	60.41666667	0.661889597
427	**GLYCTK**	961	955	979	965	29.52876377	916	858	830	868	25.46948357	0.862531319
428	**CDK5**	884	881	899	888	27.17258262	993	860	1444	1099	32.24765258	1.186771719
429	**CNKSR3**	792	790	797	793	24.26560588	912	990	1194	1032	30.28169014	1.247926398
430	**DCLK1**	2496	2494	2501	2497	76.40758874	2087	2001	1897	1995	58.53873239	0.766137675
431	**DYRK1B**	2435	2417	2456	2436	74.54100367	1869	1777	1163	1603	47.03638498	0.631013572
432	**FGFR1**	980	963	1048	997	30.50795594	785	687	247	573	16.81338028	0.551114611
433	**GNE**	1740	1729	1775	1748	53.48837209	1545	1481	1000	1342	39.37793427	0.736196162
434	**CHEK2**	1347	1342	1373	1354	41.43206854	1352	1282	1419	1351	39.64201878	0.956795549
435	**CPNE3**	1516	1513	1522	1517	46.41982864	1522	1446	1613	1527	44.80633803	0.965241349
436	**DDR1**	780	777	789	782	23.92900857	721	617	642	660	19.36619718	0.809318829
437	**DYRK2**	1657	1655	1665	1659	50.76499388	1485	1375	1073	1311	38.46830986	0.757772373
438	**FKBP1A**	3166	3164	3171	3167	96.90942472	2189	2073	1672	1978	58.0399061	0.598908788
439	**GRK4**	1761	1747	1778	1762	53.91676867	1587	1475	1174	1412	41.43192488	0.768442284
440	**CHKB**	2090	2082	2137	2103	64.35128519	1533	1451	1231	1405	41.22652582	0.640648057
441	**CRIM1**	1506	1499	1516	1507	46.11383109	1329	1207	917	1151	33.77347418	0.732393587
442	**DDR2**	2778	2774	2800	2784	85.18971848	2190	2314	1559	2021	59.30164319	0.696112679
443	**EIF2AK4**	2824	2816	2835	2825	86.44430845	2186	2138	2420	2248	65.96244131	0.763062861
444	**FLT1**	2773	2767	2800	2780	85.06731946	2268	2140	1586	1998	58.62676056	0.689180768
445	**GRK5**	2373	2360	2392	2375	72.6744186	2049	1915	1724	1896	55.63380282	0.765521127
446	**CHUK**	1985	1978	2028	1997	61.10771114	1411	1352	1167	1310	38.43896714	0.629036277
447	**CSNK1D**	1990	1979	2004	1991	60.92411261	1839	1774	1451	1688	49.53051643	0.81298708
448	**DGKB**	2280	2274	2316	2290	70.07343941	1842	1702	1838	1794	52.64084507	0.751223938
449	**EPHA6**	2505	2498	2515	2506	76.68298654	1852	1758	2165	1925	56.48474178	0.736600703
450	**FRK**	3156	3145	3185	3162	96.75642595	2380	2293	1894	2189	64.23122066	0.663844494
451	**GTF2H2**	2538	2528	2572	2546	77.90697674	1864	1771	1450	1695	49.73591549	0.638401303
452	**CIB1**	1359	1349	1387	1365	41.76866585	1088	988	360	812	23.82629108	0.570434573
453	**CSNK1G2**	1694	1688	1721	1701	52.0501836	1802	1696	2214	1904	55.8685446	1.073359223
454	**DGKI**	3408	3406	3416	3410	104.3451652	2629	2517	2198	2448	71.83098592	0.688397836
455	**EPHB3**	3537	3535	3542	3538	108.2619339	2971	2872	2269	2704	79.342723	0.732877385
456	**FUK**	3270	3268	3275	3271	100.0917993	2941	2870	2439	2750	80.69248826	0.806184811
457	**GTF2H4**	2772	2760	2787	2773	84.85312118	1956	1851	1410	1739	51.02699531	0.601356728
458	**CIB4**	1152	1144	1193	1163	35.5875153	1077	1018	878	991	29.0786385	0.817102241
459	**CSNK2A1**	1936	1934	1944	1938	59.30232558	1710	1645	1325	1560	45.77464789	0.771886219
460	**DGKK**	2494	2483	2508	2495	76.34638923	1898	1827	1654	1793	52.61150235	0.68911579
461	**ERBB2**	2548	2537	2589	2558	78.27417381	2011	1957	2362	2110	61.91314554	0.79097795
462	**GAK**	2950	2941	2977	2956	90.45287638	2241	2181	1746	2056	60.3286385	0.666962079
463	**GUCY2C**	2286	2277	2313	2292	70.13463892	1913	1802	1490	1735	50.90962441	0.725884174
464	**CKM**	1502	1497	1525	1508	46.14443084	1261	1184	597	1014	29.75352113	0.644791161
465	**DAK**	1572	1570	1580	1574	48.16401469	1404	1327	971	1234	36.20892019	0.75178368
466	**DLG1**	1865	1863	1870	1866	57.09914321	1889	1772	2078	1913	56.13262911	0.983073054
467	**ERBB4**	2697	2695	2702	2698	82.55813953	2199	2116	1385	1900	55.75117371	0.675295907
468	**GALK1**	2328	2323	2336	2329	71.26682987	1830	1707	1359	1632	47.88732394	0.671944073
469	**GUCY2D**	2292	2279	2317	2296	70.25703794	2166	2037	1905	2036	59.74178404	0.850331665
470	**CLK2**	1725	1715	1768	1736	53.12117503	1343	1277	830	1150	33.74413146	0.635229387
471	**DAPK1**	1712	1709	1727	1716	52.50917993	1461	1378	779	1206	35.38732394	0.673926426
472	**DMPK**	2694	2689	2702	2695	82.46634027	2583	2448	2382	2471	72.50586854	0.879217731
473	**ERCC2**	2391	2385	2409	2395	73.28641371	1853	1712	1268	1611	47.27112676	0.645018966
474	**GALK2**	2362	2343	2393	2366	72.39902081	2029	1940	2451	2140	62.79342723	0.867324261
475	**GUCY2F**	2310	2293	2378	2327	71.20563035	1801	1706	2163	1890	55.45774648	0.778839345
476	**CLK3**	1090	1084	1120	1098	33.59853121	1013	871	903	929	27.25938967	0.811326826
477	**DAPK2**	1587	1573	1607	1589	48.62301102	1416	1268	1048	1244	36.50234742	0.750721657
478	**DTYMK**	1069	1058	1119	1082	33.10893513	1006	852	932	930	27.28873239	0.824210513
479	**FASTKD1**	1285	1282	1300	1289	39.44308446	1173	1072	926	1057	31.01525822	0.786329433
480	**GCKR**	1278	1276	1283	1279	39.1370869	1337	1248	1603	1396	40.96244131	1.046640017
481	**HIPK3**	2325	2323	2330	2326	71.1750306	1900	1805	1545	1750	51.34976526	0.721457579
482	**HMGA2**	1309	1304	1317	1310	40.08567931	1117	957	701	925	27.14201878	0.677100133
483	**KCNH4**	2643	2640	2658	2647	80.99755202	2370	2269	1643	2094	61.44366197	0.758586654
484	**MADD**	3156	3148	3167	3157	96.60342717	2549	2560	2214	2441	71.62558685	0.741439398
485	**MAP3K7**	938	933	964	945	28.91676867	1022	915	1363	1100	32.27699531	1.116203393
486	**MARK2**	1622	1621	1626	1623	49.66340269	1676	1510	2001	1729	50.73356808	1.02154837
487	**MOS**	1052	1050	1054	1052	32.19094247	1064	951	1216	1077	31.60211268	0.981708215
488	**IDNK**	1950	1947	1959	1952	59.73072215	1800	1728	1416	1648	48.35680751	0.809580159
489	**KIAA1804**	3127	3119	3141	3129	95.74663403	2655	2483	2392	2510	73.65023474	0.769220093
490	**MAGI2**	3887	3872	3923	3894	119.1554468	3308	3201	2659	3056	89.6713615	0.752557805
491	**MAP4K1**	2536	2529	2576	2547	77.9375765	2390	2330	1982	2234	65.55164319	0.841078798
492	**MARK3**	2591	2579	2606	2592	79.31456548	2207	2029	1932	2056	60.3286385	0.760624964
493	**MPP1**	993	986	1033	1004	30.72215422	1149	1036	1697	1294	37.96948357	1.235899126
494	**IGFN1**	1714	1704	1727	1715	52.47858017	1819	1635	2318	1924	56.45539906	1.075779849
495	**KIT**	2517	2508	2553	2526	77.29498164	2249	2189	1478	1972	57.86384977	0.748610693
496	**MAP2K1**	2876	2872	2892	2880	88.12729498	2619	2429	2656	2568	75.35211268	0.855037167
497	**MAP4K4**	2057	2055	2065	2059	63.00489596	1766	1700	1619	1695	49.73591549	0.789397629
498	**MARK4**	2741	2734	2751	2742	83.90452876	2369	2297	1928	2198	64.49530516	0.768674899
499	**MPP5**	2507	2496	2536	2513	76.89718482	2287	2209	1690	2062	60.50469484	0.786825876
500	**IP6K2**	1990	1984	2020	1998	61.13831089	2249	2253	2128	2210	64.84741784	1.060667475
501	**LATS2**	2975	2970	2986	2977	91.09547124	2719	2738	2466	2641	77.49413146	0.850691373
502	**MAP2K3**	3684	3671	3709	3688	112.8518972	3207	3123	2748	3026	88.79107981	0.786792974
503	**MAP4K5**	2503	2496	2543	2514	76.92778458	2455	2312	2424	2397	70.33450704	0.914292637
504	**MAST4**	2532	2520	2547	2533	77.50917993	2012	1946	1727	1895	55.60446009	0.717391929
505	**MPP7**	2078	2071	2118	2089	63.92288862	1758	1686	1311	1585	46.50821596	0.727567495
506	**IPPK**	1518	1500	1539	1519	46.48102815	2252	2174	2729	2385	69.98239437	1.505612013
507	**LIMK1**	2769	2752	2837	2786	85.25091799	2681	2532	2515	2576	75.58685446	0.886639772
508	**MAP2K5**	2827	2817	2861	2835	86.750306	3205	3121	3802	3376	99.06103286	1.141909896
509	**MAPK13**	2776	2771	2799	2782	85.12851897	2318	2228	1916	2154	63.20422535	0.742456536
510	**MASTL**	2505	2502	2514	2507	76.71358629	2252	2162	1751	2055	60.29929577	0.786031506
511	**MYLK**	2070	2057	2089	2072	63.40269278	1656	1560	1107	1441	42.28286385	0.666893818
512	**IRAK1**	3438	3430	3482	3450	105.5691554	2634	2538	2232	2468	72.41784038	0.685975369
513	**LIMK2**	2679	2666	2698	2681	82.0379437	2550	2472	2238	2420	71.00938967	0.865567644
514	**MAP2K6**	3544	3532	3592	3556	108.8127295	3097	2995	3322	3138	92.07746479	0.846201223
515	**MAPK4**	2568	2564	2590	2574	78.76376989	2244	2089	2012	2115	62.05985915	0.787923931
516	**MATK**	3508	3506	3513	3509	107.374541	2968	2860	2653	2827	82.95187793	0.772546985
517	**MYLK4**	3317	3309	3328	3318	101.5299878	2474	2313	2008	2265	66.46126761	0.654597416
518	**IRAK4**	1373	1363	1404	1380	42.22766218	1380	1266	1494	1380	40.49295775	0.958920188
519	**LMTK2**	2897	2893	2919	2903	88.83108935	2665	2604	2426	2565	75.26408451	0.847271885
520	**MAP3K10**	2548	2543	2556	2549	77.99877601	2599	2556	2792	2649	77.72887324	0.996539654
521	**MAPK6**	2592	2580	2616	2596	79.4369645	2387	2220	1930	2179	63.93779343	0.804887168
522	**MET**	2925	2919	2961	2935	89.81028152	2664	2597	2134	2465	72.32981221	0.80536227

523	**MYO3B**	1744	1742	1749	1745	53.39657283	1910	1837	2481	2076	60.91549296	1.140812785
524	**ITK**	1396	1389	1406	1397	42.74785802	1722	1852	2567	2047	60.06455399	1.405089209
525	**LMTK3**	2489	2479	2517	2495	76.34638923	3072	2899	3479	3150	92.42957746	1.210660758
526	**MAP3K14**	2684	2679	2710	2691	82.34394125	2454	2275	1922	2217	65.0528169	0.790013399
527	**MAPKAPK2**	2288	2286	2293	2289	70.04283966	2290	2206	2380	2292	67.25352113	0.960176964
528	**MKNK1**	1631	1623	1642	1632	49.93880049	1682	1497	2017	1732	50.82159624	1.017677552
529	**N4BP2**	2551	2546	2577	2558	78.27417381	2728	2537	3429	2898	85.03521127	1.08637635
530	**ITPK1**	1310	1299	1324	1311	40.11627907	1953	1904	2317	2058	60.38732394	1.505307206
531	**LRPPRC**	1512	1505	1549	1522	46.57282742	2459	2374	2958	2597	76.20305164	1.636212699
532	**MAP3K3**	2025	2023	2033	2027	62.02570379	2001	1911	2013	1975	57.95187793	0.934320361
533	**MAPKAPK5**	2248	2246	2253	2249	68.81884945	2010	2028	1878	1972	57.86384977	0.840813966
534	**MKNK2**	2643	2640	2649	2644	80.90575275	2930	2786	3338	3018	88.55633803	1.094561697
535	**NAGK**	2536	2534	2544	2538	77.6621787	2864	2714	3392	2990	87.73474178	1.129697148
536	**KCNH3**	1058	1040	1079	1059	32.40514076	1662	1607	2029	1766	51.81924883	1.599105809
537	**LRRK2**	942	932	1003	959	29.34516524	1825	1669	1891	1795	52.67018779	1.794850612
538	**MAP3K4**	2530	2518	2545	2531	77.44798042	2939	2843	3359	3047	89.407277	1.154417152
539	**MARK1**	1844	1832	1889	1855	56.7625459	2268	2106	2769	2381	69.86502347	1.230829632
540	**MOK**	1094	1083	1123	1100	33.65973072	2208	2040	2103	2117	62.1185446	1.845485489
541	**NEK1**	1234	1228	1264	1242	38.00489596	1865	1691	2399	1985	58.24530516	1.53257373
542	**NEK2**	286	277	301	288	8.812729498	273	182	319	258	7.570422535	0.859032668
543	**NRGN**	1337	1331	1367	1345	41.15667075	1523	1426	1857	1602	47.00704225	1.142148804
544	**PCM1**	1764	1750	1784	1766	54.03916769	2197	2095	2392	2228	65.37558685	1.209781528
545	**PGK1**	2674	2666	2721	2687	82.22154223	2382	2202	1647	2077	60.94483568	0.741227105
546	**PIP4K2B**	2063	2055	2074	2064	63.15789474	2699	2591	2015	2435	71.44953052	1.131284233
547	**PRKAB2**	1321	1316	1347	1328	40.63647491	1389	1203	1758	1450	42.54694836	1.047013759
548	**NEK3**	569	567	574	570	17.44186047	784	681	1229	898	26.34976526	1.510719875
549	**NT5C2**	1605	1598	1615	1606	49.14320685	2119	2100	2474	2231	65.46361502	1.332098966
550	**PDGFRA**	1262	1258	1284	1268	38.8004896	1353	1161	1800	1438	42.19483568	1.087482043
551	**PGK2**	3171	3169	3176	3172	97.0624235	3382	3399	3998	3593	105.4284038	1.086191751
552	**PKDCC**	2941	2933	2952	2942	90.0244798	2870	2761	2766	2799	82.13028169	0.912310539
553	**PRKCB**	2501	2493	2530	2508	76.74418605	2404	2259	2237	2300	67.48826291	0.879392517
554	**NEK4**	930	924	948	934	28.58017136	1098	947	1744	1263	37.05985915	1.296698284
555	**OXSR1**	1839	1835	1855	1843	56.39534884	1750	1593	1628	1657	48.62089202	0.862143652
556	**PDGFRL**	2302	2290	2320	2304	70.50183599	2302	2188	2410	2300	67.48826291	0.957255396
557	**PGM2L1**	1897	1882	1945	1908	58.38433293	2629	2568	3053	2750	80.69248826	1.382091466
558	**PKMYT1**	2774	2765	2810	2783	85.15911873	2701	2634	2525	2620	76.87793427	0.902756339
559	**PRKCD**	2517	2506	2540	2521	77.14198286	2629	2466	3119	2738	80.34037559	1.041461116
560	**NEK8**	1210	1201	1246	1219	37.30110159	1263	1148	1510	1307	38.35093897	1.028144943
561	**PACSIN2**	1327	1319	1347	1331	40.72827417	2140	2119	2509	2256	66.1971831	1.625337298
562	**PDK1**	2593	2589	2615	2599	79.52876377	2646	2584	2852	2694	79.04929577	0.993971137
563	**PHKG2**	2522	2519	2528	2523	77.20318237	2368	2300	1974	2214	64.96478873	0.84147812
564	**PKN3**	2821	2809	2839	2823	86.38310894	2448	2374	1940	2254	66.13849765	0.765641553
565	**PRKCDBP**	1726	1714	1771	1737	53.15177479	2379	2210	2779	2456	72.0657277	1.355848003
566	**NME1**	1240	1236	1262	1246	38.12729498	1559	1498	1902	1653	48.50352113	1.272146927
567	**PAK3**	2797	2790	2807	2798	85.61811506	2268	2093	2056	2139	62.76408451	0.733070151
568	**PDK2**	2500	2495	2523	2506	76.68298654	2313	2246	1801	2120	62.20657277	0.811217397
569	**PIK3AP1**	2565	2555	2581	2567	78.5495716	2951	2878	3276	3035	89.05516432	1.133744749
570	**PLXNB2**	3239	3222	3283	3248	99.3880049	3787	3760	4021	3856	113.1455399	1.138422489
571	**PRKCG**	2937	2930	2980	2949	90.23867809	2408	2285	2411	2368	69.48356808	0.769997628
572	**NME4**	1230	1228	1235	1231	37.66829865	1553	1372	2100	1675	49.14906103	1.304785796
573	**PAN3**	1351	1343	1362	1352	41.37086903	1384	1305	1562	1417	41.5786385	1.00502212
574	**PDK3**	3382	3377	3408	3389	103.7025704	2662	2582	2061	2435	71.44953052	0.688985145
575	**PIK3C2B**	3707	3704	3713	3708	113.4638923	2866	2679	2627	2724	79.92957746	0.704449458
576	**PLXNB3**	3324	3316	3338	3326	101.7747858	3375	3302	3595	3424	100.4694836	0.987174601
577	**PRKCI**	2201	2187	2236	2208	67.56425949	2062	1976	1710	1916	56.22065728	0.832106467
578	**NME7**	855	849	891	865	26.46878825	1632	1439	1429	1500	44.01408451	1.662867378
579	**PANK1**	1226	1221	1234	1227	37.54589963	1397	1413	1891	1567	45.98004695	1.224635643
580	**PEAK1**	2629	2623	2647	2633	80.56915545	2145	2060	1669	1958	57.45305164	0.713089908
581	**PIK3CD**	3399	3391	3419	3403	104.130967	2823	2732	2389	2648	77.69953052	0.746171219
582	**PMVK**	3685	3676	3712	3691	112.9436965	3150	3058	2522	2910	85.38732394	0.756016729
583	**PRKCQ**	1832	1821	1861	1838	56.24235006	1860	1781	2005	1882	55.22300469	0.98187584
584	**NME9**	1094	1086	1126	1102	33.72093023	1652	1506	2110	1756	51.5258216	1.528007123
585	**PANK3**	1624	1616	1644	1628	49.81640147	1801	1703	2418	1974	57.92253521	1.162720179
586	**PFKFB2**	2499	2490	2526	2505	76.65238678	2382	2297	2101	2260	66.31455399	0.865133583
587	**PIK3R3**	2620	2600	2658	2626	80.35495716	2261	2170	1863	2098	61.56103286	0.766113692
588	**POMK**	2029	2012	2097	2046	62.60709914	2068	1964	2241	2091	61.3556338	0.980010808
589	**PRKCZ**	1766	1761	1795	1774	54.28396573	1927	1830	2483	2080	61.03286385	1.124325812
590	**NMRK2**	927	925	932	928	28.39657283	1431	1279	1781	1497	43.92605634	1.546878794
591	**PANK4**	1279	1277	1284	1280	39.16768666	1847	1803	2092	1914	56.16197183	1.433885343
592	**PFKL**	2576	2574	2581	2577	78.85556916	2205	2095	2099	2133	62.58802817	0.793704602
593	**PIKFYVE**	2657	2654	2663	2658	81.33414933	2901	2743	2918	2854	83.74413146	1.029630631
594	**PRKAA2**	1864	1859	1875	1866	57.09914321	1711	1595	1650	1652	48.4741784	0.848947562
595	**PRKDC**	1655	1647	1675	1659	50.76499388	1612	1483	1600	1565	45.9213615	0.904587157
596	**NRBP1**	654	643	683	660	20.19583843	682	518	912	704	20.657277	1.0228482
597	**PBK**	1455	1447	1487	1463	44.76744186	1561	1511	1905	1659	48.67957746	1.087387964
598	**PFKM**	1440	1434	1458	1444	44.18604651	1435	1318	1525	1426	41.842723	0.946966889
599	**PIM1**	1728	1725	1743	1732	52.99877601	1576	1520	1167	1421	41.69600939	0.786735327
600	**PRKAB1**	1332	1330	1337	1333	40.78947368	1388	1291	1653	1444	42.37089202	1.038770256
601	**PRPS1**	1685	1682	1691	1686	51.59118727	1549	1446	1244	1413	41.46126761	0.803650193
602	**PRPS2**	37	29	51	39	1.193390453	487	364	835	562	16.49061033	13.81828578
603	**RPS6KC1**	32	17	68	39	1.193390453	70	75	161	102	2.992957746	2.507945107
604	**SLK**	441	434	481	452	13.83108935	256	147	533	312	9.154929577	0.66190951
605	**STK32A**	22	19	28	23	0.70379437	1034	919	1515	1156	33.92018779	48.19616248
606	**TAF9**	102	92	118	104	3.182374541	482	420	781	561	16.46126761	5.172636782
607	**TRIM28**	43	37	76	52	1.591187271	68	102	85	85	2.494131455	1.567465692
608	**PRPSAP1**	554	545	566	555	16.98286414	1164	988	1016	1056	30.98591549	1.824540033
609	**RYK**	570	562	602	578	17.68665851	485	356	335	392	11.50234742	0.650340335
610	**SMG1**	430	426	446	434	13.28029376	917	782	857	852	25	1.882488479
611	**STK32B**	321	316	332	323	9.88372093	1014	832	1469	1105	32.42370892	3.280516432
612	**TBRG4**	370	362	390	374	11.44430845	945	877	1253	1025	30.07629108	2.628056664
613	**TSSK6**	23	18	46	29	0.887392901	582	520	1106	736	21.59624413	24.33673304
614	**PSKH1**	550	536	570	552	16.89106487	1183	995	1464	1214	35.62206573	2.108929543
615	**SCGB2A1**	554	546	601	567	17.3500612	1378	1237	1855	1490	43.72065728	2.519913721
616	**SNRK**	404	401	410	405	12.39290086	1151	1165	1578	1298	38.08685446	3.073280009
617	**STK33**	533	531	541	535	16.37086903	1082	935	1373	1130	33.157277	2.025382827
618	**TGFBR1**	478	473	486	479	14.65728274	788	714	1189	897	26.32042254	1.795723191
619	**TTN**	275	269	293	279	8.537331701	772	578	645	665	19.5129108	2.285598297
620	**PSKH2**	613	607	631	617	18.88004896	1540	1555	1897	1664	48.82629108	2.586131592
621	**SCYL1**	479	476	494	483	14.77968176	1297	1150	1168	1205	35.35798122	2.392337114
622	**SNX16**	521	518	527	522	15.97307222	1114	961	1090	1055	30.95657277	1.938047506
623	**STK36**	556	554	564	558	17.0746634	991	999	1076	1022	29.98826291	1.756301849
624	**TGFBR3**	324	322	329	325	9.944920441	893	734	1268	965	28.3157277	2.847255327
625	**TYRO3**	272	270	277	273	8.35373317	799	634	1252	895	26.26173709	3.143712704
626	**PTK2**	778	775	784	779	23.8372093	1644	1473	1413	1510	44.30751174	1.858754151
627	**SEPHS2**	946	939	959	948	29.00856793	1502	1422	1951	1625	47.68192488	1.643718676

628	**SOX9**	697	693	719	703	21.51162791	1459	1306	1873	1546	45.36384977	2.108805989
629	**STK38**	1029	1027	1034	1030	31.51774786	1619	1460	1445	1508	44.24882629	1.403933634
630	**TK2**	453	445	464	454	13.89228886	914	749	965	876	25.70422535	1.850251287
631	**ULK1**	56	40	93	63	1.927784578	616	498	971	695	20.39319249	10.57856398
632	**PXK**	805	782	864	817	25	1708	1537	1852	1699	49.85328638	1.994131455
633	**SGK1**	1831	1814	1899	1848	56.54834761	2381	2295	1666	2114	62.03051643	1.096946578
634	**SRC**	1199	1193	1229	1207	36.93390453	1626	1449	1563	1546	45.36384977	1.228244085
635	**STK38L**	955	942	974	957	29.28396573	1626	1443	1887	1652	48.4741784	1.655314682
636	**TNK1**	239	227	287	251	7.680538556	826	758	1128	904	26.5258216	3.453640836
637	**VRK1**	319	310	346	325	9.944920441	866	677	1169	904	26.5258216	2.667273384
638	**RIOK3**	1284	1273	1313	1290	39.47368421	2189	2065	2610	2288	67.13615023	1.700782473
639	**SGMS2**	2602	2597	2631	2610	79.86536108	2702	2572	3108	2794	81.98356808	1.026522224
640	**SRPK1**	2412	2410	2417	2413	73.8372093	2657	2583	2863	2701	79.25469484	1.07337067
641	**STK4**	2581	2578	2587	2582	79.00856793	2612	2417	2897	2642	77.52347418	0.981203383
642	**TNNI3K**	1779	1776	1788	1781	54.49816401	1991	1855	2466	2104	61.7370892	1.13282879
643	**WEE1**	330	322	344	332	10.15911873	1037	895	897	943	27.67018779	2.723679931
644	**RNASEL**	1530	1524	1557	1537	47.03182375	2662	2570	3132	2788	81.80751174	1.739407602
645	**SH3BP5**	1912	1910	1920	1914	58.56793146	2910	2830	3278	3006	88.20422535	1.506015718
646	**SRPK2**	2725	2723	2730	2726	83.41493268	2946	2960	3295	3067	89.99413146	1.078873153
647	**STK40**	2525	2514	2539	2526	77.29498164	3108	2960	3508	3192	93.66197183	1.211747126
648	**TPK1**	993	982	1034	1003	30.69155447	2189	2202	2737	2376	69.71830986	2.271579627
649	**XYLB**	1021	1014	1046	1027	31.42594859	1515	1522	1775	1604	47.0657277	1.497670868
650	**ROR1**	1977	1974	1992	1981	60.61811506	2389	2235	2867	2497	73.26877934	1.208694452
651	**SIK3**	2589	2582	2599	2590	79.25336597	3385	3386	3669	3480	102.1126761	1.288433303
652	**STK11IP**	2506	2486	2544	2512	76.86658507	3236	3231	3616	3361	98.62089202	1.283013834
653	**STYK1**	1626	1616	1687	1643	50.2753978	2572	2474	2757	2601	76.32042254	1.518047114
654	**TRIB1**	1216	1204	1231	1217	37.23990208	2242	2082	2180	2168	63.61502347	1.708248946
655	**ZMYND8**	678	671	718	689	21.08323133	1301	1290	1648	1413	41.46126761	1.966551851
656	**ROS1**	1072	1060	1087	1073	32.83353733	2241	2196	2478	2305	67.63497653	2.059935725
657	**SLAMF6**	1640	1628	1685	1651	50.52019584	2286	2120	2557	2321	68.10446009	1.348064056
658	**STK24**	2237	2232	2260	2243	68.63525092	2608	2436	2675	2573	75.49882629	1.100000733
659	**SYK**	2532	2530	2540	2534	77.53977968	2912	2895	3487	3098	90.90375587	1.172349938
660	**TRIB2**	1559	1557	1564	1560	47.73561812	1794	1708	1985	1829	53.66784038	1.124272451
661	**ABL2**	1137	1130	1147	1138	34.82252142	2020	1901	2394	2105	61.76643192	1.773749557
662	**ADRBK1**	417	412	440	423	12.94369645	304	179	75	186	5.457746479	0.421652849
663	**CALM2**	625	615	641	627	19.18604651	1047	916	1406	1123	32.95187793	1.71749182
664	**CIT**	547	538	583	556	17.01346389	397	305	15	239	7.012910798	0.412197707
665	**EFNA4**	95	89	113	99	3.029375765	200	102	601	301	8.832159624	2.915504813
666	**GCK**	681	678	696	685	20.96083231	374	237	748	453	13.29225352	0.634147219
667	**IQCH**	164	157	174	165	5.048959608	88	65	120	91	2.670187793	0.528859013
668	**ADRBK2**	389	369	427	395	12.0869033	1119	1068	1545	1244	36.50234742	3.01999168
669	**CAMK1D**	870	860	931	887	27.14198286	1158	1101	1509	1256	36.85446009	1.357839635
670	**CKMT1B**	557	548	569	558	17.0746634	1297	1234	1537	1356	39.78873239	2.330279166
671	**EGFR**	531	525	561	539	16.49326805	1392	1323	1152	1289	37.82276995	2.293224716
672	**GK2**	965	963	973	967	29.58996328	1195	1166	1413	1258	36.91314554	1.247488724
673	**IRAK2**	320	317	326	321	9.82252142	178	180	245	201	5.897887324	0.600445351
674	**AK2**	336	329	349	338	10.34271726	1264	1121	1785	1390	40.78638498	3.943488346
675	**CAMK2A**	431	416	464	437	13.37209302	837	851	1126	938	27.52347418	2.058277199
676	**CSNK1A1**	374	367	417	386	11.81150551	1085	936	1531	1184	34.74178404	2.941351042
677	**EIF2AK1**	656	654	661	657	20.10403917	1518	1526	1996	1680	49.29577465	2.452033357
678	**HIPK1**	370	356	387	371	11.35250918	825	670	1181	892	26.17370892	2.305543956
679	**IRAK3**	346	338	393	359	10.98531212	378	217	599	398	11.67840376	1.063092576
680	**AK3**	531	528	537	532	16.27906977	1196	1029	1555	1260	36.97183099	2.271126761
681	**CCL3**	1080	1071	1095	1082	33.10893513	1558	1454	2043	1685	49.44248826	1.493327649
682	**CSNK1G1**	1075	1070	1104	1083	33.13953488	1797	1722	2049	1856	54.4600939	1.643357219
683	**EPHA3**	669	667	674	670	20.50183599	1577	1404	1990	1657	48.62089202	2.371538435
684	**HK2**	395	384	409	396	12.11750306	912	831	1332	1025	30.07629108	2.482053516
685	**ITPKB**	470	459	511	480	14.6878825	270	160	473	301	8.832159624	0.601322868
686	**AKAP12**	637	632	660	643	19.67564259	1533	1413	1029	1325	38.87910798	1.976001942
687	**CDC42BPG**	1424	1416	1438	1426	43.63525092	1696	1708	2497	1967	57.71713615	1.322718099
688	**CSNK2A2**	1115	1109	1142	1122	34.33292534	1902	1776	2496	2058	60.38732394	1.758874997
689	**EPHA4**	1752	1745	1765	1754	53.67197062	2286	2292	2529	2369	69.5129108	1.295143629
690	**HMGXB3**	585	581	607	591	18.08445532	1027	895	973	965	28.3157277	1.565749545
691	**KCNH1**	490	488	495	491	15.0244798	1117	979	1069	1055	30.95657277	2.060408957
692	**AXL**	1551	1549	1556	1552	47.49082007	1968	1968	2331	2089	61.29694836	1.290711516
693	**CDK1**	1321	1319	1326	1322	40.45287638	1950	1944	2331	2075	60.88615023	1.505113003
694	**CSNK2B**	1784	1781	1790	1785	54.62056304	2365	2249	2724	2446	71.77230047	1.314016123
695	**ETNK1**	1216	1214	1224	1218	37.27050184	2001	2000	2524	2175	63.82042254	1.712357478
696	**IGF1R**	1599	1591	1610	1600	48.95960832	2177	2114	2561	2284	67.01877934	1.368858568
697	**KCNH2**	587	576	619	594	18.17625459	850	838	1162	950	27.87558685	1.533626563
698	**BAZ1B**	981	966	1017	988	30.23255814	1737	1719	2169	1875	55.01760563	1.819813109
699	**CDK12**	2493	2486	2533	2504	76.62178703	2811	2724	3417	2984	87.55868545	1.142738754
700	**DGKA**	2076	2069	2086	2077	63.55569155	3056	2912	3467	3145	92.28286385	1.451999995
701	**FER**	3101	3096	3124	3107	95.07343941	3124	3055	3247	3142	92.19483568	0.969722314
702	**IGSF22**	2268	2266	2276	2270	69.46144431	2370	2295	2745	2470	72.47652582	1.043406548
703	**KCNH8**	1162	1149	1178	1163	35.5875153	1346	1196	1214	1252	36.7370892	1.03230273
704	**BMPR1A**	2518	2505	2567	2530	77.41738066	2956	2932	3580	3156	92.6056338	1.196186606
705	**CDKL4**	4427	4415	4460	4434	135.6793146	3953	3797	3680	3810	111.7957746	0.823970662
706	**DGKG**	2491	2479	2527	2499	76.46878825	3136	3155	3477	3256	95.5399061	1.249397412
707	**FGFR1OP**	2368	2363	2397	2376	72.70501836	3082	3001	3484	3189	93.57394366	1.287035555
708	**ILK**	2052	2049	2058	2053	62.82129743	2229	2142	2847	2406	70.59859155	1.123800279
709	**KHK**	1167	1153	1187	1169	35.77111383	1564	1402	2002	1656	48.5915493	1.358401908
710	**BRAF**	1543	1534	1591	1556	47.61321909	2508	2340	3126	2658	77.99295775	1.638052609
711	**CDKL5**	2547	2544	2556	2549	77.99877601	2833	2740	3388	2987	87.64671362	1.123693449
712	**DNAJC6**	2495	2481	2515	2497	76.40758874	2757	2770	3143	2890	84.80046948	1.109843549
713	**FGGY**	1889	1881	1936	1902	58.20073439	2162	1988	2618	2256	66.1971831	1.137394292
714	**INSR**	3811	3806	3819	3812	116.6462668	3355	3256	2983	3198	93.83802817	0.804466621
715	**LAMTOR3**	1609	1600	1630	1613	49.35740514	2177	2184	2362	2241	65.75704225	1.332262952
716	**CALM1**	1657	1651	1684	1664	50.91799266	2586	2597	2902	2695	79.0786385	1.553058838
717	**CERK**	2488	2486	2496	2490	76.19339045	2578	2583	2837	2666	78.22769953	1.026699285
718	**DOLK**	2593	2591	2598	2594	79.37576499	2781	2780	3346	2969	87.1185446	1.09754589
719	**FYN**	1974	1971	1980	1975	60.43451652	2215	2208	2942	2455	72.03638498	1.191974208
720	**IPMK**	1532	1527	1543	1534	46.94002448	1978	1965	2528	2157	63.29225352	1.348364306
721	**LATS1**	2212	2203	2233	2216	67.80905753	2243	2122	2448	2271	66.63732394	0.982720102
722	**LTBP1**	1781	1770	1813	1788	54.7123623	1788	1695	1881	1788	52.46478873	0.958920188
723	**MLKL**	2573	2568	2599	2580	78.94736842	2380	2253	1907	2180	63.96713615	0.810250391
724	**NMRK1**	2526	2523	2532	2527	77.3255814	2427	2328	2226	2327	68.28051643	0.883026228
725	**PIK3C2A**	2559	2554	2570	2561	78.36597307	2361	2228	1894	2161	63.40962441	0.809147413
726	**PNKP**	1899	1890	1920	1903	58.23133415	1903	1884	1922	1903	55.83920188	0.958920188
727	**SEPHS1**	2608	2600	2637	2615	80.01835985	2615	2569	2661	2615	76.73122066	0.958920188
728	**LY6G5B**	2317	2311	2335	2321	71.02203182	2171	2146	1746	2021	59.30164319	0.834975312
729	**MNAT1**	2556	2548	2576	2560	78.33537332	2410	2428	1942	2260	66.31455399	0.846546728
730	**NRK**	2700	2696	2722	2706	82.80293758	2506	2367	2045	2306	67.66431925	0.817172932
731	**PIK3C2G**	2538	2536	2543	2539	77.69277846	2439	2334	2244	2339	68.63262911	0.883384923
732	**PRKCSH**	2508	2506	2513	2509	76.7747858	2484	2477	2416	2459	72.15375587	0.939810579

733	**SGK3**	2698	2690	2709	2699	82.58873929	2512	2367	2099	2326	68.25117371	0.826398057
734	**LYN**	2886	2881	2912	2893	88.5250918	2893	2841	2945	2893	84.88849765	0.958920188
735	**MPP6**	3563	3561	3568	3564	109.0575275	3564	3576	3552	3564	104.5774648	0.958920188
736	**NRP1**	2607	2602	2615	2608	79.80416157	2608	2457	2759	2608	76.5258216	0.958920188
737	**PIK3R4**	2588	2584	2604	2592	79.31456548	2497	2493	2216	2402	70.48122066	0.88862897
738	**PRKD3**	2488	2483	2499	2490	76.19339045	2490	2379	2601	2490	73.06338028	0.958920188
739	**SGK494**	2599	2596	2614	2603	79.65116279	2603	2486	2720	2603	76.37910798	0.958920188
740	**MAGI3**	2736	2729	2746	2737	83.75152999	2737	2743	2731	2737	80.31103286	0.958920188
741	**MYLK2**	2538	2528	2566	2544	77.84577723	2544	2486	2602	2544	74.64788732	0.958920188
742	**NUAK2**	3166	3160	3193	3173	97.09302326	3173	3055	3291	3173	93.10446009	0.958920188
743	**PIP4K2A**	2929	2927	2937	2931	89.6878825	2931	2807	3055	2931	86.00352113	0.958920188
744	**PRPF4B**	2545	2542	2551	2546	77.90697674	2396	2406	1936	2246	65.90375587	0.845928807
745	**SH3BP5L**	2483	2481	2491	2485	76.04039168	2485	2489	2481	2485	72.91666667	0.958920188
746	**MAK**	2619	2614	2627	2620	80.17135863	2620	2463	2777	2620	76.87793427	0.958920188
747	**MYO3A**	2796	2793	2811	2800	85.67931457	2800	2798	2802	2800	82.15962441	0.958920188
748	**PANK2**	3068	3063	3076	3069	93.91064871	3069	2906	3232	3069	90.0528169	0.958920188
749	**PIP5K1A**	2545	2532	2570	2549	77.99877601	2549	2541	2557	2549	74.79460094	0.958920188
750	**RAF1**	3332	3320	3377	3343	102.2949816	3343	3329	3357	3343	98.092723	0.958920188
751	**SHC1**	4430	4426	4452	4436	135.7405141	4436	4416	4456	4436	130.1643192	0.958920188
752	**MAP3K1**	2495	2486	2507	2496	76.37698898	2496	2515	2477	2496	73.23943662	0.958920188
753	**NEK10**	2863	2858	2892	2871	87.85189718	2871	2845	2897	2871	84.24295775	0.958920188
754	**PASK**	2720	2717	2726	2721	83.2619339	2721	2738	2704	2721	79.8415493	0.958920188
755	**PIP5K1B**	2858	2851	2871	2860	87.51529988	2860	2873	2847	2860	83.92018779	0.958920188
756	**RFK**	3201	3192	3228	3207	98.13341493	3207	3038	3376	3207	94.10211268	0.958920188
757	**SIK2**	2484	2480	2506	2490	76.19339045	2470	2481	2399	2450	71.88967136	0.943515847
758	**MAP3K13**	2052	2050	2057	2053	62.82129743	2053	2058	2048	2053	60.24061033	0.958920188
759	**NEK11**	3236	3231	3244	3237	99.05140759	3237	3173	3301	3237	94.98239437	0.958920188
760	**PDIK1L**	2588	2584	2604	2592	79.31456548	2592	2417	2767	2592	76.05633803	0.958920188
761	**PLK4**	2603	2598	2614	2605	79.7123623	2605	2614	2596	2605	76.43779343	0.958920188
762	**RIOK1**	2517	2506	2540	2521	77.14198286	2371	2244	2048	2221	65.17018779	0.844808305
763	**SKP2**	2556	2547	2592	2565	78.48837209	2400	2278	2027	2235	65.58098592	0.835550339
764	**MAP3K19**	2180	2174	2198	2184	66.82986536	2034	1906	1712	1884	55.28169014	0.827200382
765	**NEK5**	2668	2665	2683	2672	81.7625459	2672	2679	2665	2672	78.40375587	0.958920188
766	**PDXK**	3104	3102	3109	3105	95.0122399	3105	3106	3104	3105	91.10915493	0.958920188
767	**PLK5**	2616	2611	2624	2617	80.07955936	2617	2620	2614	2617	76.7899061	0.958920188
768	**RPS6KA3**	2604	2601	2619	2608	79.80416157	2608	2474	2742	2608	76.5258216	0.958920188
769	**STAP1**	2858	2856	2863	2859	87.48470012	2859	2719	2999	2859	83.89084507	0.958920188
770	**MAPK9**	2541	2539	2546	2542	77.78457772	2462	2392	2292	2382	69.8943662	0.898563292
771	**NME5**	2760	2755	2768	2761	84.48592411	2761	2615	2907	2761	81.01525822	0.958920188
772	**PFKFB1**	2588	2579	2609	2592	79.31456548	2592	2440	2744	2592	76.05633803	0.958920188
773	**PLXNA3**	3439	3434	3465	3446	105.4467564	3446	3370	3522	3446	101.1150235	0.958920188
774	**RPS6KB1**	2491	2488	2497	2492	76.25458996	2482	2477	2457	2472	72.53521127	0.951224199
775	**STK17B**	3082	3080	3090	3084	94.36964504	3084	3081	3087	3084	90.49295775	0.958920188
776	**MELK**	1371	1369	1376	1372	41.98286414	1372	1361	1383	1372	40.25821596	0.958920188
777	**NME8**	1966	1959	1976	1967	60.18971848	1967	1958	1976	1967	57.71713615	0.958920188
778	**PHKA1**	2517	2510	2542	2523	77.20318237	2373	2215	2081	2223	65.22887324	0.844898762
779	**PLXNA4**	2559	2553	2577	2563	78.42717258	2479	2315	2394	2396	70.30516432	0.896438849
780	**SCYL2**	3447	3439	3467	3451	105.5997552	3451	3281	3621	3451	101.2617371	0.958920188
781	**STK19**	2879	2874	2902	2885	88.28029376	2885	2870	2900	2885	84.65375587	0.958920188
782	**STK3**	284	275	299	286	8.751529988	286	269	303	286	8.392018779	0.958920188
783	**TNK2**	1639	1629	1673	1647	50.39779682	1647	1624	1670	1647	48.32746479	0.958920188
784	**CD2**	1485	1475	1513	1491	45.62423501	1491	1409	1573	1491	43.75	0.958920188
785	**WNK2**	1709	1704	1735	1716	52.50917993	1716	1695	1737	1716	50.35211268	0.958920188
786	**DYRK3**	1562	1549	1578	1563	47.82741738	1563	1444	1682	1563	45.86267606	0.958920188
787	**PIK3CB**	2544	2537	2587	2556	78.2129743	2356	2329	1783	2156	63.2629108	0.808854431
788	**STK31**	1555	1550	1563	1556	47.61321909	1556	1380	1732	1556	45.657277	0.958920188
789	**TTBK1**	2793	2790	2808	2797	85.5875153	2797	2620	2974	2797	82.07159624	0.958920188
790	**CNKSR1**	2866	2864	2871	2867	87.72949816	2867	2883	2851	2867	84.12558685	0.958920188
791	**TAOK1**	1930	1923	1940	1931	59.08812729	1931	1748	2114	1931	56.66079812	0.958920188
792	**GK**	2008	2004	2030	2014	61.62790698	2014	1926	2102	2014	59.09624413	0.958920188
793	**PKN2**	1397	1394	1403	1398	42.77845777	1398	1408	1388	1398	41.02112676	0.958920188
794	**STK39**	1841	1839	1849	1843	56.39534884	1843	1847	1839	1843	54.0786385	0.958920188
795	**TTK**	3419	3417	3424	3420	104.6511628	3420	3231	3609	3420	100.3521127	0.958920188
796	**GSAP**	2901	2887	2918	2902	88.8004896	2902	2707	3097	2902	85.15258216	0.958920188
797	**EIF2AK2**	2490	2479	2540	2503	76.59118727	2403	2309	2197	2303	67.57629108	0.882298519
798	**GK5**	1877	1871	1895	1881	57.55813953	1881	1889	1873	1881	55.19366197	0.958920188
799	**PPIP5K2**	1960	1951	1981	1964	60.09791922	1964	1942	1986	1964	57.62910798	0.958920188
800	**STRADA**	2314	2309	2340	2321	71.02203182	2171	2071	1821	2021	59.30164319	0.834975312
801	**ULK2**	3018	3016	3023	3019	92.38066095	3019	3033	3005	3019	88.58568075	0.958920188
802	**NIM1K**	3104	3096	3115	3105	95.0122399	3105	3092	3118	3105	91.10915493	0.958920188
803	**MAP2K4**	2517	2511	2544	2524	77.23378213	2449	2457	2216	2374	69.65962441	0.901932063
804	**ICK**	2603	2601	2611	2605	79.7123623	2605	2607	2603	2605	76.43779343	0.958920188
805	**PRKACB**	2126	2123	2132	2127	65.08567931	2127	2123	2131	2127	62.41197183	0.958920188
806	**TBCK**	2804	2796	2818	2806	85.8629131	2806	2796	2816	2806	82.33568075	0.958920188
807	**ULK4**	3000	2994	3027	3007	92.01346389	3007	2991	3023	3007	88.23356808	0.958920188
808	**PLXNA2**	3020	3018	3028	3022	92.47246022	3022	3024	3020	3022	88.67370892	0.958920188
809	**NPRL2**	2627	2618	2639	2628	80.41615667	2628	2503	2753	2628	77.11267606	0.958920188
810	**ITPKA**	2524	2519	2553	2532	77.47858017	2532	2354	2710	2532	74.29577465	0.958920188
811	**ROCK1**	2002	2000	2007	2003	61.29130967	2003	1819	2187	2003	58.77347418	0.958920188
812	**TBK1**	3483	3474	3495	3484	106.6095471	3484	3502	3466	3484	102.2300469	0.958920188
813	**XRCC6BP1**	3036	3025	3071	3044	93.14565483	3044	3040	3048	3044	89.31924883	0.958920188
814	**SCYL3**	3038	3032	3065	3045	93.17625459	3045	2855	3235	3045	89.34859155	0.958920188
815	**CDKL1**	2705	2703	2713	2707	82.83353733	2707	2660	2754	2707	79.43075117	0.958920188
816	**MAP3K6**	3320	3318	3325	3321	101.621787	3321	3268	3374	3321	97.4471831	0.958920188
817	**STK17A**	2897	2888	2909	2898	88.67809058	2898	2917	2879	2898	85.03521127	0.958920188
818	**TGFB2**	2810	2805	2839	2818	86.23011016	2818	2808	2828	2818	82.68779343	0.958920188
819	**AKT3**	2829	2818	2843	2830	86.59730722	2830	2843	2817	2830	83.0399061	0.958920188
820	**STAP2**	2481	2470	2522	2491	76.22399021	2441	2382	2350	2391	70.1584507	0.920424797
821	**CDKL2**	2773	2768	2796	2779	85.03671971	2779	2714	2844	2779	81.54342723	0.958920188
822	**MAP3K8**	2944	2942	2952	2946	90.14687882	2946	2875	3017	2946	86.44366197	0.958920188
823	**CDK8**	2651	2649	2656	2652	81.1505508	2652	2636	2668	2652	77.81690141	0.958920188
824	**TGFBR2**	2503	2501	2508	2504	76.62178703	2454	2466	2292	2404	70.5399061	0.920624653
825	**CAB39**	2678	2675	2684	2679	81.97674419	2679	2602	2756	2679	78.60915493	0.958920188
826	**TRRAP**	2508	2501	2521	2510	76.80538556	2495	2501	2444	2480	72.76995305	0.94745899
827	**CERKL**	3294	3287	3319	3300	100.9791922	3300	3278	3322	3300	96.83098592	0.958920188
828	**MAPK8**	2751	2745	2769	2755	84.30232558	2755	2762	2748	2755	80.83920188	0.958920188
829	**GRK1**	2508	2500	2528	2512	76.86658507	2512	2484	2540	2512	73.70892019	0.958920188
830	**TLK1**	2661	2654	2686	2667	81.60954712	2667	2639	2695	2667	78.25704225	0.958920188
831	**CDK11A**	2907	2904	2922	2911	89.07588739	2911	2912	2910	2911	85.41666667	0.958920188
832	**PFKFB4**	2717	2710	2727	2718	83.17013464	2718	2713	2723	2718	79.75352113	0.958920188
833	**DGKD**	3083	3079	3105	3089	94.52264382	3089	2968	3210	3089	90.63967136	0.958920188
834	**PI4K2B**	2980	2978	2985	2981	91.21787026	2981	2898	3064	2981	87.47065728	0.958920188
835	**TRIO**	3132	3127	3140	3133	95.86903305	3133	3044	3222	3133	91.93075117	0.958920188
836	**TLK2**	1533	1527	1551	1537	47.03182375	1537	1537	1537	1537	45.09976526	0.958920188
837	**ASB10**	2586	2583	2601	2590	79.25336597	2440	2455	1975	2290	67.19483568	0.847848351

838	**EFNB3**	2725	2711	2742	2726	83.41493268	2726	2715	2737	2726	79.98826291	0.958920188
839	**DGKH**	2479	2471	2526	2492	76.25458996	2472	2455	2429	2452	71.94835681	0.94352821
840	**PIK3CA**	2499	2494	2507	2500	76.499388	2500	2405	2595	2500	73.35680751	0.958920188
841	**VRK3**	1620	1611	1641	1624	49.69400245	1624	1445	1803	1624	47.65258216	0.958920188

N.B.- % Cell viability = Average number of live cells in Kinase KO treated with vehicle control OR 5FU/ Average number of live cells in -ve control sg RNA treated with vehicle control OR 5FU
Survival fraction = % cell viability of Kinase KO treated with 5FU/ % cell viability of Kinase KO treated with vehicle control

Table S2: Quantification score (raw data) of 60 kinases in secondary screening from high content analyser. This is in reference to figure 1E.

		%Cell viability							
**SL NO.**	**KINASES**	**H357 5FUR UT**	**H357 5FUR 5FU**	**SCC4 5FUR UT**	**SCC4 5FUR 5FU**	**SCC9 5FUR UT**	**SCC9 5FUR 5FU**	**H357 CISR UT**	**H357 CISR CIS**
1	**MINK1**	105.7051575	24.05294386	95.25857263	30.77866437	101.4764556	62.24588263	93.84902144	90.91332712
2	**AGK**	82.79324509	48.4710178	70.18968895	68.85183239	86.52284853	83.57689631	95.99254427	93.34808947
3	**CDC42BPB**	83.20401643	30.94477408	81.7525921	63.92246803	73.06889353	72.41939225	91.33271202	86.416589
4	**CDK16**	91.2825194	45.36741214	72.46723048	64.00210235	73.64880538	63.74391093	95.80615098	89.20083877
5	**CDK19**	80.51118211	35.14376997	78.91761033	68.27050186	89.09765716	56.43702157	76.73578751	84.22646785
6	**CMPK2**	91.19123688	45.73254222	69.73577333	72.70613344	97.12363721	63.65112503	96.73811743	97.84482759
7	**CNTLN**	94.15791876	43.95253309	82.63653304	81.83222642	88.74971004	51.00904662	83.51584343	93.06849953
8	**CSF1R**	96.85075308	44.54586947	87.67738545	63.97024862	79.67988866	65.08930643	93.67427773	88.09412861
9	**CSK**	101.8712916	51.71154724	78.47962158	68.81997866	85.08466713	74.50707492	95.58480895	83.55079217
10	**DAPK3**	58.92286627	47.42126883	71.2010448	71.60717983	61.58663883	60.40361865	89.24743709	85.60111836
11	**DGUOK**	83.93427659	38.24737563	80.47844299	78.39998726	79.93504987	66.96822083	90.15610438	92.96365331
12	**FN3K**	92.83432223	39.02327704	69.33760173	68.31031902	81.37323127	83.60009279	89.49207829	90.31919851
13	**FN3KRP**	88.31583752	44.27202191	93.29160495	86.90493255	100.6263048	80.65414057	96.49347623	89.99301025
14	**IKBKB**	97.94614331	32.63350068	90.59996496	85.71838119	98.86337277	79.0999768	77.12022367	85.84575955
15	**MAP2K2**	86.71839343	44.91099954	88.03573988	76.1144823	98.79378334	65.27487822	84.05172414	92.14818267
16	**MAP3K12**	90.09584665	45.82382474	63.01463679	54.78044818	90.72141035	78.00974252	88.68825722	89.85321528
17	**MAP3K15**	90.3696942	47.92332268	75.23054135	73.6697087	90.44305266	75.92205985	78.76281454	92.96365331
18	**MAP3K2**	105.2031036	43.67868553	79.06891554	65.81776482	101.7165391	87.93783345	77.37651445	101.7124884
19	**MAPK12**	102.1907805	48.42537654	93.17215347	81.47387198	90.83739272	82.16191139	85.06523765	89.3639329
20	**MAPK15**	91.55636696	47.42126883	76.64803224	66.44687595	81.2572489	61.65622825	92.20643057	86.68452936
21	**MAPK3**	97.48973072	61.06800548	102.6088203	63.90654116	89.90953375	74.57666435	83.88863001	81.40726934
22	**MAST2**	97.07895938	45.869466	79.40337968	77.72309555	88.98167479	85.13106008	70.8527493	92.27632805
23	**MERTK**	82.24554998	38.20173437	84.83444025	80.28732062	105.2424032	78.98399443	77.08527493	91.27446412
24	**MFHAS1**	77.08808763	33.95709722	75.21461449	75.23054135	98.46903271	77.52261656	86.00885368	92.60251631
25	**MORN1**	104.2446372	23.32268371	93.01288483	82.94710689	110.1136627	69.7749942	96.01584343	93.17334576
26	**MPP2**	93.01688727	48.9274304	100.5542549	71.2010448	103.1547205	87.03317096	87.12721342	91.65890028
27	**MPP3**	78.8224555	52.67001369	94.70909583	80.09619826	96.07979587	78.63604732	96.38863001	92.68406337
28	**MST1R**	86.71839343	53.58283889	80.58993104	67.17951168	96.84527952	99.44328462	88.72320596	91.51910531
29	**MYLK3**	94.29484254	37.97352807	95.83990316	70.57193368	105.1496173	97.17003016	87.88443616	93.04520037
30	**NADK2**	106.4354176	42.8115016	81.52165257	74.75273544	63.11760612	75.17977267	87.1971109	90.38909599
31	**NEK6**	91.73893199	53.12642629	98.92971475	65.44348352	66.73625609	74.55346787	86.55638397	85.26328052
32	**NEK7**	83.56914651	41.62482885	90.05048816	77.70716868	103.7578288	93.62096961	95.98089469	88.39701771
33	**NLK**	95.52715655	47.42126883	67.54582955	37.10162932	93.50498724	79.03038738	98.63699907	86.28844362
34	**NME2**	90.96303058	39.7535372	71.73459474	66.63003488	83.1361633	60.91394108	99.72041007	90.87837838
35	**NOL9**	81.69785486	46.46280237	81.05181009	80.96421234	107.1213176	57.29529112	98.08946878	90.06290774
36	**NPR2**	84.57325422	50.34230945	74.04399	65.65849619	97.44838785	94.17768499	104.2054986	94.38490214
37	**NRBP2**	79.09630306	47.32998631	111.8782551	89.94696354	96.96126189	82.16191139	97.87977633	94.38490214
38	**NTRK2**	90.8261068	38.06481059	113.2001848	58.40380971	103.2011134	101.1598237	96.6332712	84.57595527
39	**NTRK3**	92.46919215	45.00228206	107.5939287	50.30499944	88.72651357	98.77058687	98.64864865	91.67054986
40	**OBSCN**	86.67275217	43.67868553	73.01670728	72.80169462	74.27511018	73.46323359	93.72087605	87.66309413
41	**PACSIN1**	88.81789137	46.23459607	108.0000637	76.34542182	78.84481559	74.92461146	94.12861137	79.63653308
42	**PACSIN3**	81.60657234	43.67868553	85.36799019	78.32831637	109.3249826	99.48967757	96.08574091	86.13699907
43	**PAK7**	80.41989959	39.5253309	108.8601144	71.4001306	89.8631408	95.89422408	102.7143523	85.11183597
44	**PFKP**	74.44089457	36.2391602	92.32802969	76.61617851	105.5207608	93.48179077	99.74370923	83.88863001
45	**PIP4K2C**	91.46508444	32.08580557	95.68063453	73.18393935	99.69844584	94.94316864	95.09552656	89.93476235
46	**PIP5K1C**	77.49885897	46.32587859	88.25075254	84.7468425	98.79378334	92.18278822	100.139795	91.01817335
47	**PKM**	87.58557736	36.96942036	78.97335436	69.07480848	89.77035491	78.35768963	98.48555452	79.99767008
48	**PKN1**	96.80511182	35.41761753	88.15519136	70.28525013	98.86337277	63.25678497	93.73252563	85.91565704
49	**ROCK2**	113.2359653	32.04016431	87.7410929	73.11226846	98.21387149	93.08745071	106.0344828	91.78704567
50	**ROR2**	95.43587403	27.84116842	107.6974533	52.42741332	89.42240779	77.08188355	101.66589	92.41612302
51	**SBK1**	107.4851666	28.11501597	55.34585185	25.04499339	99.44328462	62.25933658	89.10764212	85.29822926
52	**SGK223**	108.2154267	37.83660429	87.51811681	70.2772867	93.15704013	82.09232197	97.21575023	84.61090401
53	**SGMS1**	107.8046554	41.98995892	115.214933	73.18393935	101.8789144	72.04824867	103.6929171	88.01258155
54	**SH3BP4**	94.3404838	44.63715199	79.84933187	87.58182426	108.0259801	55.20760844	98.36905871	87.36020503
55	**SQSTM1**	94.5686901	43.81560931	103.3016389	61.94753691	95.2447228	81.9763396	103.1686859	85.65936626
56	**STK16**	94.75125513	31.21862163	83.37713221	76.13040916	95.17513338	81.11807005	98.4389562	89.02609506
57	**TEK**	79.64399817	20.35600183	84.22921942	73.97231911	101.8325215	62.97842728	95.74790308	84.71575023
58	**FKBP1A**	83.52350525	24.64628024	60.20354532	27.68088936	105.0336349	65.53003943	95.61975769	90.90167754
59	**GTF2H2**	72.75216796	40.62072113	70.30914043	10.78248682	91.3941081	65.41405706	62.67474371	74.04473439
60	**GTF2H4**	86.53582839	45.32177088	61.95550034	27.48180356	65.36766412	57.10971932	73.26421249	81.29077353

UT- Untreated, **5FU-** 5-Fluorouracil, **CIS-** Cisplatin

N.B.- % Cell viability = Average number of live cells in Kinase KO UT well OR treated with 5FU / Average number of live cells in -ve control sg RNA UT OR treated with 5FU

Table S3a

chemotherapy-responder patient details

Sl No	Tumor samples	Age/Sex	Site of disease	Clinical stage	Chemotherapy (NACT)	Cycle
1	**Patient#1**	42/M	Tongue Rt lateral border	T4aN1M0	Docetaxel + Cisplatin+ 5FU	2
2	**Patient#2**	67/M	Tongue Lt lateral border	T4aN1Mx	Docetaxel + Cisplatin+ 5FU	3
3	**Patient#3**	50/M	Rt- Buccal mucosa	T4aN2bM0	Docetaxel + Cisplatin+ 5FU	3
4	**Patient#4**	75/M	Oral cavity	T3N2bM0	Doceaqualip + Carboplatin	3
5	**Patient#5**	46/M	Tongue	T3N1M0	Docetaxel + Cisplatin+ 5FU	3
6	**Patient#6**	35/M	Tongue	T4aN2eM0	Docetaxel + Cisplatin+ 5FU	3
7	**Patient#7**	38/M	Right Buccal Mucosa	T4bN2bM0	Docetaxel + Cisplatin+ 5FU	3
8	**Patient#8**	34/M	Left Buccal Mucosa	T4aN2bMx	Docetaxel + Cisplatin+ 5FU	3
9	**Patient#9**	40/M	Tongue	T2N2cM0	Docetaxel + Cisplatin+ 5FU	3
10	**Patient#10**	45/M	Tongue	T2N1M0	Docetaxel + Cisplatin+ 5FU	3
11	**Patient#11**	51/M	Tongue Lt. lateral border	T4aN2cM0	Docetaxel + Cisplatin+ 5FU	3

Chemotherapy Doses: **Cisplatin**: 100mg. **Docetaxel**: 100mg, **5FU**:1000mg, **Doceaqualip**: 80mg, **Carboplatin:** Post chemo samples were collected after 2nd or 3rd cycle of chemotherapy regimen.

Table S3b

Chemotherapy-non-responders patient Details

Sl No	Tumor samples	Age /Sex	Site of disease	Clinical stage	Chemotherapy (NACT)	Cycle
1	**Patient# 1**	76/M	Tongue Rt lateral border	T4N0M0	Paclitaxel + Cisplatin	3
2	**Patient#2 (PDC#2)**	51/M	Rt- Buccal mucosa	T2N2bM0	Docetaxel + Cisplatin+ 5FU	2
3	**Patient# 3**	60/M	Tongue Rt lateral border	T3N1M0	Paclitaxel + Cisplatin +5FU	3
4	**Patient#4**	33/M	Rt- Lower Alveolar mucosa	T3N1Mx	Docetaxel + Cisplatin	3
5	**Patient#5**	60/F	Tongue Lt lateral border	T4N0M0	Docetaxel + Cisplatin+ 5FU	3
6	**Patient#6**	59/M	Tongue Rt lateral border	T4aN1M0	Docetaxel + Cisplatin+ 5FU	3
7	**Patient#7**	46/M	Tongue	T4N3M0	Docetaxel + Cisplatin+ 5FU	3
8	**Patient#8**	55/F	Rt- Buccal Mucosa	T4aN2M0	Docetaxel + Cisplatin+ 5FU	2
9	**Patient#9**	37/M	Tongue	T4N3M0	Docetaxel + Cisplatin+ 5FU	2
10	**Patient#10**	27/M	Lt-Buccal Mucosa	T4N2M0	Docetaxel + Cisplatin+ 5FU	2
11	**Patient#11**	46/F	Rt- oral cavity	T4N1M0	Docetaxel + Cisplatin+ 5FU	3
12	**Patient#12**	42/M	Rt- Buccal Mucosa	TxN3bM0	Paclitaxel + Cisplatin+ 5FU	2
13	**Patient#13**	30/M	Tongue Rt lateral border	T2N0Mx	Paclitaxel + Cisplatin+ 5FU	3
14	**Patient#14**	52/M	Rt- Buccal Mucosa	T4N2M0	Docetaxel + Cisplatin+ 5FU	3
15	**Patient#15**	32/M	Tongue	T3N1M0	Docetaxel + Cisplatin+ 5FU	3
16	**Patient#16**	35/M	Tongue	T4aN2aM0	Docetaxel + Cisplatin+ 5FU	3
17	**Patient#17**	36/M	Left Buccal Mucosa	T4aN2aM0	Docetaxel + Cisplatin+ 5FU	3
18	**Patient #18**	38/M		T4bN2bMO	Docetaxel + Cisplatin+ 5FU	2
19	**Patient # 19**	55/M	Tongue, Left lateral border,		Docetaxel + Cisplatin+ 5FU	3
20	**Patient # 20**	35/M	Tongue	T4aN2eM0+	Docetaxel + Cisplatin+ 5FU	3
21	**Patient # 21**	36/M	Left Buccal Mucosa	cT4aN2aM0	Docetaxel + Cisplatin+ 5FU	3
22	**Patient # 22**	39/M	Right mandible	cT4bN0Mx	Docetaxel + Cisplatin+ 5FU	3
23	**Patient # 23**	55/M	Tongue, Left lateral border,	cT4aN2cM0	Docetaxel + Cisplatin+ 5FU	3

Chemotherapy Doses: **Cisplatin**: 100mg. **Paclitaxe**l: 260 mg, **Docetaxel**: 100mg, **5FU**:1000mg **Lt-Left, Rt-Right**

Post chemo samples were collected after 2nd or 3rd cycle of chemotherapy regimen.

PDC2: patient derived cells isolated from indicated OSCC patient.

Table S4

Oligos for SgRNA and ShRNA

Sg and Sh RNA primers	Oligo sequence
MINK1 sg RNA F (SgRNA#1)	CACCCGCAACATCGCCACCTACTA
MINK1 sg RNA R (SgRNA#1)	AAACTAGTAGGTGGCGATGTTGCG
MINK1 sg RNA F (SgRNA#2)	CACCGTGGTCGGCAATGGAACCTA
MINK1 sg RNA R (SgRNA#2)	AAACTAGGTTCCATTGCCGACCAC
MINK1 3' UTR sh RNA F (ShRNA#1)	CCGGAATGTAGTGGCCTTGGATATCCTCGAGGATATCCAAGGCCACTACATTTTTTTG
MINK1 3' UTR sh RNA R (ShRNA#1)	AATTCAAAAAAATGTAGTGGCCTTGGATATCCTCGAGGATATCCAAGGCCACTACATT

Oligos for qRT-PCR

qRT PCR Primers	Primer sequence
18S qRT F	GTAACCCGTTGAACCCCATT
18S qRT R	CCATCCAATCGGTAGTAGCG
GAPDH qRT F	TCGGAGTCAACGGATTTGGT
GAPDH qRT R	TTGCCATGGGTGGAATCATA
OCT4 qRT F	CGACCATCTGCCGCTTTGAG
OCT4 qRT R	CCCCCTGTCCCCCATTCCTA
SOX2 qRT F	CACCTACAGCATGTCCTACTC
SOX2 qRT R	CATGCTGTTTCTTACTCTCCTC
Nanog qRT F	CAACTGGCCGAAGAATAGCA
Nanog qRT R	GCAGGAGAATTTGGCTGGAA
ABCC1 qRT F	AGTGGAACCCCTCTCTGTTTAAG
ABCC1 qRT R	CCTGATACGTCTTGGTCTTCATC
ABCC2 qRT F	AATCAGAGTCAAAGCCAAGATGCC
ABCC2 qRT R	TAGCTTCAGTAGGAATGATTTCAGGAGCAC
ABCC3 qRT F	TCCTTTGCCAACTTTCTCTGCAACTAT
ABCC3 qRT R	CTGGATCATTGTCTGTCAGATCCGT
ABCC4 qRT F	TGATGAGCCGTATGTTTTGC
ABCC4 qRT R	CTTCGGAACGGACTTGACAT
ABCC5 qRT F	AGAGGTGACCTTTGAGAACGCA
ABCC5 qRT R	CTCCAGATAACTCCACCAGACGG
ABCG2 qRT F	CCGCGACAGTTTCCAATGACCT
ABCG2 qRT R	GCCGAAGAGCTGCTGAGAACTGTA
MDR1 qRT F	AGGAAGCCAATGCCTATGACTTTA
MDR1 qRT R	CAACTGGGCCCCTCTCTCTC

Oligos to detect genomic cleavage detection assays

HPRT1 GCD F	TACACGTGTGAACCAACCCG
HPRT1 GCD R	GTAAGGCCCTCCTCTTTTATTT
